# Factors impacting antimicrobial resistance in the South East Asian food system and potential places to intervene: A participatory, one health study

**DOI:** 10.3389/fmicb.2022.992507

**Published:** 2023-01-05

**Authors:** Irene Anna Lambraki, Mohan Vishnumurthy Chadag, Melanie Cousins, Tiscar Graells, Anaïs Léger, Patrik John Gustav Henriksson, Max Fredrik Troell, Stephan Harbarth, Didier Wernli, Peter Søgaard Jørgensen, Carolee Anne Carson, Elizabeth Jane Parmley, Shannon E. Majowicz

**Affiliations:** ^1^School of Public Health Sciences, University of Waterloo, Waterloo, ON, Canada; ^2^WorldFish, Penang, Malaysia; ^3^Global Economic Dynamics and the Biosphere, Royal Swedish Academy of Sciences, Stockholm, Sweden; ^4^Stockholm Resilience Center, Stockholm University, Stockholm, Sweden; ^5^Global Studies Institute, University of Geneva, Geneva, Switzerland; ^6^Beijer Institute of Ecological Economics, Royal Swedish Academy of Sciences, Stockholm, Sweden; ^7^Infection Control Program and WHO Collaborating Center on Patient Safety, Geneva University Hospitals and Faculty of Medicine, Geneva, Switzerland; ^8^Foodborne Disease and Antimicrobial Resistance Surveillance Division, Public Health Agency of Canada, Guelph, ON, Canada; ^9^Department of Population Medicine, Ontario Veterinary College, University of Guelph, Guelph, ON, Canada

**Keywords:** antibiotics, antimicrobial resistance, antimicrobial use, one health, food system, participatory approach, causal loop diagram, South East Asia

## Abstract

**Background:**

With AMU projected to increase, South East Asia (SEA) is at high risk of experiencing disproportionate health, social, and economic burdens due to antimicrobial resistance (AMR). Our objective was to identify factors influencing AMR in SEA’s food system and places for intervention by integrating the perspectives of experts from the region to inform policy and management decisions.

**Materials and methods:**

We conducted two 6.5 h workshops and two 90-min interviews involving 18 AMR and other disciplinary experts from human, animal, and environment sectors who brainstormed the factors influencing AMR and identified leverage points (places) for intervention. Transcripts and workshop materials were coded for factors and their connections and transcribed into a causal loop diagram (CLD). Thematic analysis described AMR dynamics in SEA’s food system and leverage points for intervention. The CLD and themes were confirmed *via* participant feedback.

**Results:**

Participants constructed a CLD of AMR in the SEA food system that contained 98 factors interlinked by 362 connections. CLD factors reflected eight sub-areas of the SEA food system (e.g., government). Seven themes [e.g., antimicrobial and pesticide use and AMR spread (*n* = 40 quotes)], six “overarching factors” that impact the entire AMR system [e.g., the drive to survive (*n* = 12 quotes)], and 10 places for intervention that target CLD factors (*n* = 5) and overarching factors (*n* = 2) emerged from workshop discussions.

**Conclusion:**

The participant derived CLD of factors influencing AMR in the SEA food system demonstrates that AMR is a product of numerous interlinked actions taken across the One Health spectrum and that finding solutions is no simple task. Developing the model enabled the identification of potentially promising leverage points across human, animal, and environment sectors that, if comprehensively targeted using multi-pronged interventions, could evoke system wide changes that mitigate AMR. Even targeting some leverage points for intervention, such as increasing investments in research and capacity building, and setting and enforcing regulations to control antimicrobial supply, demand, and use could, in turn, shift mindsets that lead to changes in more difficult to alter leverage points, such as redefining the profit-driven intent that drives system behavior in ways that transform AMU and sustainably mitigate AMR.

## Background

Antimicrobial resistance (AMR) claimed an estimated 1.27 million lives in 2019 alone (Murray et al., 2002) and is projected to cause serious adverse health, social, and economic impacts long-term if not properly addressed (O’Neill, 2016; Council of Canadian Academies, 2019). While AMR occurs naturally, it is behavior in human medicine and animal health/food production that cause AMR to develop and spread. This makes AMR a social ecological problem (Jørgensen et al., 2016; Lambraki et al., 2022). Antimicrobials modernized medicine (Laximinaryan et al., 2013) and have been instrumental in augmenting agricultural food production systems (Durso and Cook, 2014; Grace, 2015), but inappropriate antimicrobial use (AMU) in humans, animals, and crops (Finley et al., 2013; Shallcross and Davies, 2014; Van Boeckel et al., 2015; O’Neill, 2016) and the spread of AMR (Collignon et al., 2018) are causes of rising AMR across human, animal, and agricultural and environmental systems. Conditions, such as trade (Hanefeld et al., 2017), international travel (Frost et al., 2019), and poverty and poor sanitation (Alividza et al., 2018; Collignon and McEwen, 2019) are some factors that have accelerated AMR spread along the food chain, into the environment, and across geographic borders.

While AMR is found across the world, South East Asia (SEA) has been described as at high risk for infectious diseases and AMR (Chereau et al., 2017). AMU, including inappropriate AMU, is reportedly high in SEA (Holloway et al., 2017). Population growth and economic development have translated into increased demand for animal protein and the intensification of pig, poultry, and aquaculture production systems. All these production systems rely on AMU to prevent and treat diseases and increase growth rates, and as such, AMU is projected to increase significantly over time (Richter et al., 2015; Van Boeckel et al., 2015; Cabello et al., 2016; Zellweger et al., 2017; Garza et al., 2022). Poverty, increasing urbanization, and poor sanitation are some added factors that contribute to infectious diseases and AMR development and spread in the region (Coker et al., 2011; Zellweger et al., 2017).

While effective actions in high-income regions are commonly applied in low-middle income regions, SEA is a hotspot for AMR (Chereau et al., 2017; Zellweger et al., 2017), suggesting a need to understand the SEA context to determine how to effectively intervene using tailored approaches (Kakkar et al., 2018). Calls for a One Health approach have been made, which emphasizes engaging actors from human, animal, and environment sectors to understand the web of connections that give rise to AMR and find sustainable solutions [World Health Organization (WHO), 2015; Robinson et al., 2016]. A systems lens and systems thinking tools, such as causal loop diagrams (CLD), can be used to integrate and visually illustrate diverse stakeholders’ perspectives about how actions from their respective sector interlink with other components of the SEA system and contribute to the AMR problem (Kim, 1994; Williams and Hummelbrunner, 2009). This can be helpful to build a shared mental model of this complex issue. CLDs in turn enable stakeholders to identify leverage points, or “places” for intervention that have potential to desirably change system behavior to address the problem of interest (Meadows, 1999). CLDs therefore can help to understand how a particular system operates so that better decisions can be made about where and how to intervene (e.g., to reduce AMU or contain AMR levels) while minimizing impacts on other parts of the system (e.g., economics), and help to identify more tailored, effective, and sustainable actions. To this end, our study objectives were to engage diverse stakeholders to identify the system of factors that impact AMR in SEA.

This paper begins with an overview of the participatory approach used to engage diverse stakeholders in discussions at workshops and our approach to the interviews. The results section follows with an overview of the CLD of AMR in the SEA food system constructed based on participants input, themes describing key AMR dynamics in the CLD that emerged from participant discussions, and a table listing leverage points for intervention that participants identified as potentially promising to address AMR in SEA, which the research team further classified as having less or greater potential to mitigate AMR. The paper concludes with a discussion of the findings and conclusion.

## Materials and methods

This qualitative study used a One Health approach (Robinson et al., 2016) and brought together stakeholders representing different sectors in two workshops to develop a CLD (Kim, 1994; Williams and Hummelbrunner, 2009) relevant to AMR in the SEA food system. We defined the SEA food system as including all actors and actions involved in producing, collecting, processing, distributing, consuming, and disposing food products that originate from agriculture, fisheries, forestry, and parts of the broader natural, social, and economic environments in which they are embedded (FAO, 2018). Our definition also includes other systems (trade, environment, and health) that connect with and may take actions that change the food system (FAO, 2018). We focused on SEA because it is a hotspot for AMR (Chereau et al., 2017), and it is an area of population and economic growth (Coker et al., 2011; Zellweger et al., 2017) comprised of different SEA countries with potentially varying regulations and ways of operating, which offer a relevant context to explore the system of factors that may impact or be impacted by AMR.

The study was designed and conducted by a core Canadian team who consulted with a larger interdisciplinary and international project team of academic researchers and collaborators with specialties in human and veterinary medicine, aquaculture, clinical microbiology, and evolutionary biology, during the design, data collection, and analysis steps. The core team has disciplinary backgrounds in public health, epidemiology, and veterinary medicine and brings expertise in participatory and qualitative methods, systems thinking, food safety, one health, and AMR.

We followed the Consolidated Criteria for Reporting Qualitative Research (COREQ) checklist for reporting qualitative research (Tong et al., 2007). The study received ethics approval from the University of Waterloo’s Research Ethics Committee (ORE# 40519). All participants provided written consent to participate in the study and permission for anonymous quotations to be used, which are provided in the results section and Supplementary materials A–C.

### Participant recruitment and characteristics

We purposively selected AMR experts (e.g., aquaculture sciences and veterinary medicine) and experts in other content areas who may not usually be engaged in AMR discussions but work in sectors whose actions may indirectly impact AMR (e.g., pest risk management and food industry). Efforts to select participants from different SEA countries were made. Participants were identified through: Google, LinkedIn, and Twitter searches; websites of professional organizations that may impact AMR in human, animal, agricultural, and environment sectors; and through the professional networks of our interdisciplinary team of researchers and collaborators. Thirty-seven participants were approached *via* email with a maximum of two follow-up contacts as per the University of Waterloo Ethics Committee approved protocols. Seventeen individuals did not respond, and two individuals declined due to work conflicts. Eighteen participants agreed to participate. Fifteen participants (80%) were male. Participants represented the following areas of expertise: environmental technologies and water quality; physician; clinical microbiology; veterinarian; aquaculture; animal health policy and economics; animal welfare; pharmacology; medicine use and safety; health systems and economics; food aid; food safety; nutrition; and tropical pest management in agricultural plants. These participants came from research and academic, healthcare, not-for-profit and governmental organizations, industry, and private consultancy organizations. Nine participants (50%) were from Malaysia and the remaining were from Thailand, Indonesia, Laos, Singapore, Sri Lanka, India, and Ethiopia. To our knowledge, most participants had experience living in the SEA region and all participants had experience working in the region. One participant had a professional connection with a member of our research team and four had a professional connection with a collaborator.

### Data collection and analysis

Our approach to data collection and analysis were intentionally comparable to a similar study conducted in Sweden (Lambraki et al., 2022). Two 6.5 h in-person workshops were conducted on October 9 and 10, 2019 at WorldFish (Penang, Malaysia). Two 30–60-min online interviews were conducted with participants who could not attend the workshops. Workshops and interviews were audio-recorded, guided by a semi-structured interview guide, and facilitated by two team members. Two additional team members attended the workshops to provide expert input if requested by participants. Each workshop and interview started with a welcome and a brief presentation on AMR to provide common understanding and terminology. The purpose of the workshop or interview was then described, which was to have participants brainstorm the factors that influence AMR in the SEA food system and then identify leverage points (places) to target interventions. A systems thinking activity followed in the workshops to prepare participants for the modeling process. Interviewees opted to skip the system thinking activity and dove into the model building exercise.

To initiate conversation, workshop participants were shown a large, laminated poster, and given handouts of an existing initial CLD of AMR in the Canadian food system (Majowicz et al., 2018) and tasked to adapt it to reflect the SEA food system. Interviewees were also tasked to adapt the CLD and were sent the CLD of AMR in the Canadian food system (Majowicz et al., 2018) *via* email in advance. After defining AMR, we defined the factors influencing AMR as any factors associated with AMU, AMR, or AMR impacts, either proximally or distally. Facilitators added or removed factors and connections and changed the names of factors directly on the laminated poster as directed by individual participants and group discussion. Prior to adding a given factor or connection to the CLD, workshop participants were asked if they had anything to add to the point made by another participant, or to question, counter, or revise it. For interviewees, we reflected what we heard and asked participants to confirm if our understanding was correct. Each factor was written as a short textual phrase. Participants were prompted to frame factors as “measurable” (e.g., “amount of exported food products” vs. “exported food products”) for clarity and to enable future simulation modeling. Facilitators made efforts to elicit the direction, and where possible, the nature of the connections between factors and implicitly inferred them when missing based on the verbatim transcripts. The direction of connections between factors was depicted by an arrow (➔), and where participants identified it, a positive (+) or negative (−) sign on the arrow was used to identify the nature of the connection. A positive connection indicated that two factors moved in the same direction (i.e., more “good farming practices” led to increased “animal welfare”). A negative connection indicated that two factors moved in the opposite direction [an increase in “non-antimicrobial infection control on farms of food producing animals” (e.g., vaccination) led to a decrease in “food producing animal illness”]. If participants did not identify the nature of the relationships between factors or our team could not discern it from the transcripts, no sign was added to the arrow.

Then, through small group discussions, workshop participants identified leverage points or places in the CLD to target interventions with potential of changing the behavior of the system in ways that could desirably mitigate AMR and they also suggested associated actions, which were discussed with the larger group. Each interviewee also identified leverage points and provided suggested associated actions. Revisions and discussions continued until workshop participants and interviewees had no new information to share and indicated the CLD and identification of leverage points were complete.

The workshops produced the following data sources, which were analyzed: the facilitator-revised laminated CLDs; each participant’s own marked-up handout of the starting CLD; and verbatim workshop and interview transcripts. Data sources were open coded, triangulated, and thematically analysed using NVivo 12 (QSR International, United States), and all factors and connections were entered into Vensim Professional 8.0.4 Double Precision (Ventana Systems, Inc., United States) to yield two CLDs of AMR in the SEA food system, one for each workshop. Five co-authors then met at key points during the analysis process to discuss the workshop findings, combine the two CLDs because of their similarities, and to finalize any areas of uncertainty about factors and connections (e.g., their placement in the CLDs). Participants were sent a summary report of workshop discussions and the draft combined CLD for feedback to ensure both items reflected their understanding of workshop discussions. Feedback was incorporated to produce the final CLD of AMR in the SEA context. Our research team then classified the participant-identified leverage points for intervention (Meadows, 1999) as “shallow” (places where interventions are easier to implement but may have less potential to transform the behavior of the whole system and create sustainable change; Abson et al., 2017), or “deep” (places in the system that are more difficult to alter yet have greater potential to change the behavior of the whole system that are sustainable; Abson et al., 2017). We used work of Meadows (1999) and Abson et al. (2017) on leverage points, participant discussions of leverage points coupled with their suggested associated interventions and our team’s expertise in AMR to make the classifications.

## Results

**Figure 1:**
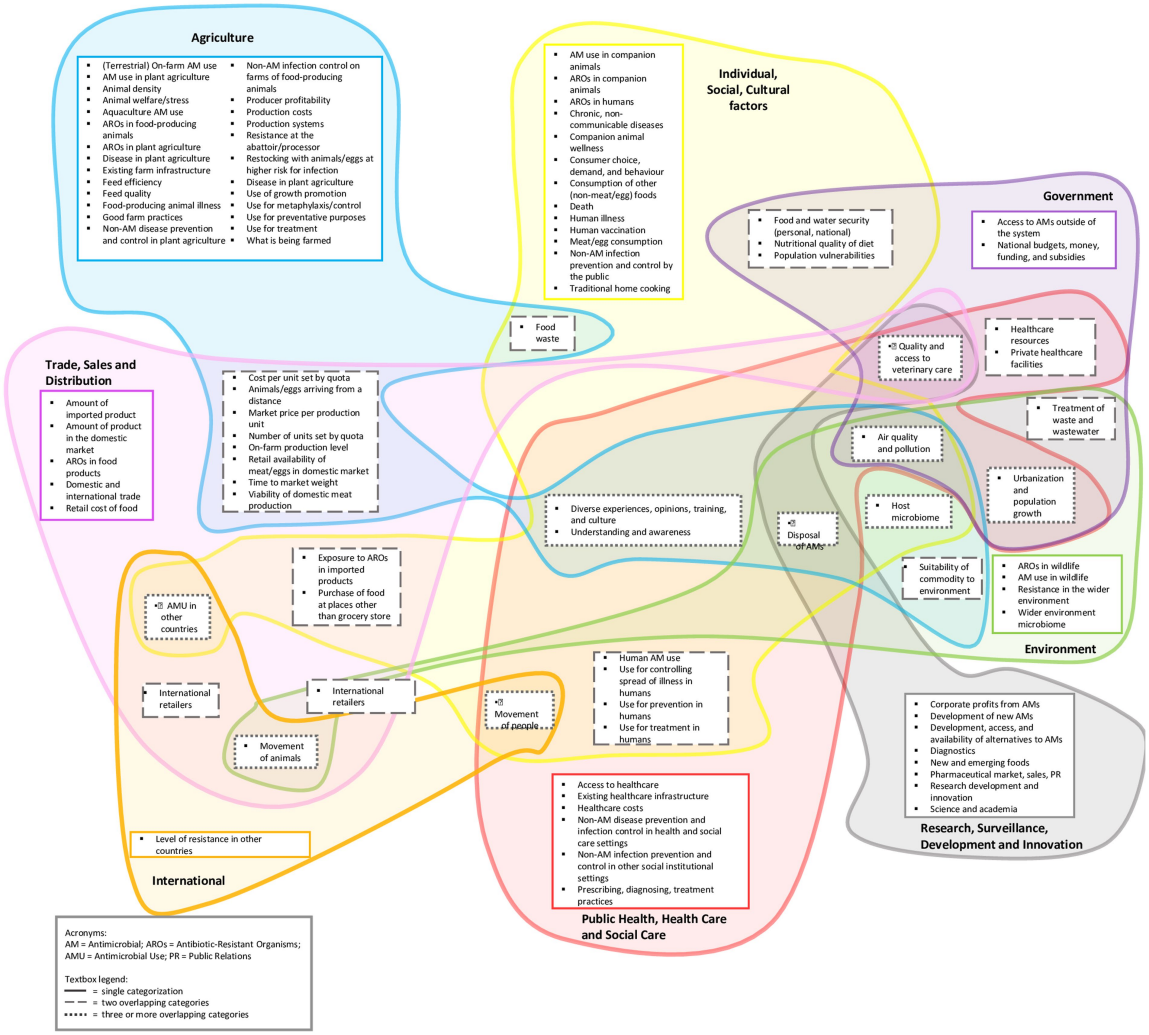
Ninety eight CLD factors grouped into eight One Health sub-areas.

The CLD of AMR in the SEA food system contained 98 factors and 362 connecting arrows (Supplementary material D); for ease of presentation, we grouped the CLD factors into eight sub-areas of the One Health spectrum (Figure 1) and defined them (Supplementary material E). Seven themes emerged from workshop discussions and interviews that described the key dynamics in the CLD: antimicrobial and pesticide use and AMR spread; agricultural food production systems; consumer demands; access to antimicrobials, diagnostics and alternatives; food safety; population growth and migration; and awareness and understanding of AMR (Table 1). Participants also identified six “overarching factors” not included in the CLD because they exert broad impacts on the entire system depicted in the CLD: drive for survival; leadership priorities; governance, regulations, and enforcement; changing socioeconomic structures; climate change; and the intent driving the system (Table 2).

**Table 1:** Themes based on participant discussions.

Theme	Description of Theme
Antimicrobial and pesticide use and AMR spread	The role of human behavior in AMR development and spread in animals, people, and the environment, such as inappropriate antimicrobial, pesticide and/or chemical use; poor infection prevention and control measures; poor sanitation and waste management; and the transport of people and animals across porous borders. Example quote: “…*you get antimicrobials getting into water and then again people getting exposed to antimicrobials through drinking water is one thing*. *Then you have also people getting exposed to antimicrobial resistant bacteria through water*. *So there is another arrow that connects water…to resistant human infection*…” (day 1 workshop).
Agricultural food production systems	How regulated and unregulated food production systems use antimicrobials, pesticides, and chemicals to produce food quickly to meet market demands, and how low animal welfare standards can contribute to AMR, and the challenges farmers face in implementing good farm practices. Example quote: “*Ideally we want and know ‘high’ welfare is best associated with reduced AMU but ‘higher’ might be more realistic in South East Asia*” (Interview A).
Consumer demand	How consumer demand for food and antibiotics influences the use of antibiotics, fungicides, pesticides and chemicals and the factors (e.g., religious and cultural practices, finances) that shape these demands. Example quote: “*Some people go straight to the pharmacy shop*. *They do not want to go to the doctor*. *They do not have time to wait at OPD (outpatient department) or they do not have the money*” (day 1 workshop).
Access to antimicrobials, diagnostics and alternatives	How public and privatized healthcare, over the counter and online sales of antimicrobials, and access to alternative health providers enable access to different types of antimicrobials (including counterfeit antimicrobials) depending on what people can access or afford, and how a lack of availability of appropriate diagnostics or alternatives to antimicrobials impact clinical diagnosis and/or antibiotic prescribing practices. Example quote: “*It is certainly a challenge*. *The access issue you talk about*, *the lack of access to antibiotics and especially in very rural areas*, *compared to in big cities where there is all private medicine and drivers toward using more expensive antibiotics*, *more broad-spectrum antibiotics*” (day 1 workshop).
Food safety	The influence of cultural, religious and food safety practices in SEA on potential food borne illnesses, and how improper use of antimicrobial compounds along the food chain can contribute to AMR. Example quote: “*And you may well have covered it but it is food preparation practices…It is whether people for cultural reasons eat cooked food*, *or whether they eat raw food*. *It is a large culture particularly in [name of country] and probably in parts of [name of country] as well of eating raw food*. *Raw meat*. *Blood*. *Raw fish*, *which increases transmission dynamics for chances…that maybe affect to the human health…that leads to the hospital*” (day 1 workshop).
Population growth and migration	How increased population growth and migration of people from rural to urban areas for employment opportunities and animals for cross-border trade contribute to infectious disease and AMR spread. Also highlights how migration of people impacts resources (e.g., access to clean water) and the food chain based on changing lifestyles and consumption patterns. Example quote: “…*population growth*. *We are more people with more and our consumption patterns*, *our diets that are also changing you know to the worst*, *consumption of more meat for instance*, *so there is much more demand for meat*, *and it is forecasted to be growing*, *and these will lead to expansion and intensification of livestock production with the consequence of using more and more antibiotics if we follow the same pattern*” (day 2 workshop).
Awareness and understanding of AMR	How awareness of issues relevant to AMR (e.g., what antimicrobials to use, when, and how; food safety; and alternatives to antimicrobials) have on AMU practices and AMR spread. Example quote: “…*I see from the authority, all the way to the farmers, they do not understand the consequences of that*. …*They do not understand it*. *They do not even know what is an antibiotic…They do not know why they use it to treat disease*” (day 2 workshop).

**Table 2:** Overarching factors based on participant discussions.

Overarching factor	Description
Drive to survive	How food insecurity, poverty and environmental conditions (e.g., pollution, pests, bacterial, viral, and zoonotic diseases) can impact human and animal health and alter markets and trade and drive people, businesses and leaders to do what they need to survive even if it leads to unintended consequences that can impact AMR. Example quote: “*You have got huge pollution issues leading to respiratory infections*. *That leads to health seeking behavior*. *That leads to antibiotic use*. *Then you have got water scarcity issues*. *That leads to drinking, whether it is in a village or a city*. *That leads drinking difficult water*. *Let us put it that way*. *That leads to diarrhea*. *That leads to again health seeking behavior*” (day 1 workshop).
Leadership priorities	How leadership, particularly national government’ decisions to spur economic development and improve food security have ripple effects on antibiotic, pesticide, or chemical use, the microbiome, economic security, and livelihoods and nutritional intake of citizens. Example quote: “…*rice, government invests money*. *Not for the sake of making money*. *They invest in rice because they need to have rice, because people need rice*. *So it is an uneconomical crop…but I think there are not getting anywhere economically*, *and the countries are not getting anywhere nutritionally*, *because there is too much rice*” (day 2 workshop).
Changing socio-economicstructures	How economic development in the SEA region is increasing wealth and changing consumer lifestyles and demands that can impact nutritional intake, health, and immunity. How changing socioeconomic structures leads to fewer people choosing careers in agricultural food production, creating increased labor costs to produce foods in systems that already rely on antimicrobials, pesticides, and chemicals to meet market demands. Example quote: “*Socioeconomic status has increased… Changing food behavior actually…consumption…and I think it might change nutritional composition of that…*. *there is right now a change that is happening in the structure of the socioeconomic status*” (day 1 workshop).
Governance, regulationsand enforcement	Governance mechanisms that exist to address AMR in SEA and challenges with implementation which may contribute to mass, potentially illegal and inappropriate AMU and AMR development and spread. Example quote: “…*It is not just about inappropriate prescribing practices*, *but rather unethical prescribing practices where there are kickbacks for pharmacists and physician if they prescribe drugs - kickbacks from pharmaceutical industry*…” (Interview B).
Climate change	How global warming will increase heat stress in animals, increase pests and insects and potentially impact the acidity of oceans that in turn could impact food production and supply, change food consumption patterns, cause disease, and impact AMR. Example quote: “…*if you want to wash your vegetables or you cook*, *you do not have the proper water*. *Okay*. *Rivers are drying up*. *Temperatures are varying nowadays…So and the other thing there is I have a feeling*, *I mean I was*, *I am not sure about it*, *global warming is changing the acidity of oceans*. *Therefore the so called microbiome in oceans is changing*. *So therefore I do not know how it affects fish*, *but that is another issue altogether and that is what is going up into the clouds and then causing rain*. *So acid rain and what not*. *Okay coming down into the soil and that is where we are growing our vegetables and getting water*. *The links within climate change and the environment are …*.*the water cycle is influenced by climate change*” (day 1 workshop).
Underlying intent driving system behavior	How the underlying values and goals of economic prosperity that drive system behavior and in turn global problems like AMR and climate change and a lack of systems thinking contribute to short-term solutions that may not effectively mitigate AMR over time. Example quote: “*So it is a total mindset change*. *The economy is a very consumptive base*, *but it is big business*. *You cannot run this world without a business*, *because the business guys are the third force yea*. *The invisible force*” (day 2 workshop).

### Themes

#### Antimicrobial and pesticide use and AMR spread

Participants described a range of actions in diverse settings that influenced AMR development or spread across the One Health spectrum. Some AMR-promoting practices relevant to food production that participants described were the use of antibiotic-containing feed from manufacturers; antibiotic use, including banned antibiotics, in food animals as insurance to protect against illness; high, mass, or inappropriate antibiotic use; use for growth promotion, metaphylactic and treatment purposes; lack of antibiotic rotation; use of inexpensive pesticides instead of expensive antibiotics; and high use fungicides in some areas to grow conventional crops or prevent post-harvest loss. Inappropriate use of antimicrobial cleaning agents on-farm and in industry (e.g., hospitals and food establishments) were additional identified contributing factors. Critically important human antibiotics (e.g., ciprofloxacin and colistin in poultry farms), counterfeit drugs and transgenic species were said to add to selection pressures and the accumulation of antimicrobial-resistant organisms (AROs), and AMR genes in agricultural settings. AMR genes were also said to have been found in some genetically engineered animals. AMR was also said to be linked to some probiotics. In humans, certain diseases such as tuberculosis which requires long term treatment with antibiotics that can also be used for other infections were also noted to potentially generate resistance to those agents and become a focus for dissemination.

Effluent from farms (e.g., high density agricultural industry areas), the pharmaceutical industry, and human waste were said to seep antibiotics into the environment, spurring the development of AROs, which have the potential to spread. Water was identified as an important medium for AMR spread. Participants described the recycling of untreated wastewater and manure in agriculture as spreading antimicrobials and AROs into waterways and drinking water that reach terrestrial livestock, aquaculture, and humans. Food waste and poorly contained waste management systems were identified as pathways for AMR spread to wildlife. Although described as a relatively small contributor and not quantified in the SEA context, AMR was also said to potentially spread from companion animals to people. AMR was also noted to spread to veterinarians, farm workers, slaughterhouse workers, and their respective families through exposure to farm animals and the slaughtering process of farm animals; relating to a citation from Interview A: “*and then through products being contaminated through that whole system …into wet markets …or through retailers like supermarkets*” (Interview A). The inhumane animal slaughter in formal and informal settings was also noted to create a “*hotbed of AMR risk for workers and food systems*” (Interview A). Wet markets where people access fresh food and live animals were described as high-risk areas for stressed animals shedding high levels of fecal bacteria and AROs. Poor transport conditions of animals across porous domestic and regional borders were identified as increasing infectious, endemic, or exotic disease outbreaks that compromise herd/flock immunity, increase disease vulnerability, AMU, and AMR spread, with impacts on markets and trade. International trade and human travel were also said to contribute to the spread of infections with resistant bacteria across geographic borders. SEA countries’ capacity to deal with health tourism, hospitals with insufficient beds and overcrowded outpatient departments and poor infection prevention and control measures were all said to contribute to AMR and “*infected people infecting each other and then of course [people can acquire] hospital associated infections*” (day 1 workshop).

#### Agricultural food production systems

Both regulated and less regulated farming systems in SEA were described as influencing the use of antimicrobials. Vertically integrated farming systems, led by a few large-scale companies, were described as regulated with government agreements to uphold antimicrobial stewardship practices and proper care for their animals. These entities were described as owning farms, processing plants, and delivery channels that transport food to wet markets and supermarkets, with some also offering ‘raised antibiotic-free’ market certifications. Participants noted that while these systems have greater control over AMU practices, inappropriate AMU may still occur to control endemic diseases or on contracted farms with less oversight. Smallholder farms that are unregulated and thus have less oversight of AMU practices were said to be widespread across SEA. To ensure cost-effectiveness, many of these farms were said to be integrated farm systems where crops, livestock, and aquaculture are raised on the same plot of land and contribute to resistance due to run-off of antibiotics, pesticides, fungicides, or chemicals into the environment. While government officers and technical representatives from the private sector that provide technical assistance and advice on farm management to farmers exist, participants noted that they do not necessarily reach all farmers, creating missed opportunities to influence change. While participants stressed that “*everybody wants their animals to be healthy and live in a good way*” (day 1 workshop) because live animals are a key product in SEA, low animal welfare, and a lack of biosecurity in food production systems were identified by participants as contributing to the AMR problem. High animal densities in barren environments and confinement; short weaning times; and practices such as castration, dehorning, tail docking, and teeth clipping, were identified as reducing animal welfare, increasing behavioral stress in livestock, lowering immunity, predisposing animals to sub-clinical or clinical disease, and potentially increasing AMU for metaphylactic, prophylactic, or treatment purposes. The food industry’s growing use of fast growth genetics to accelerate food production, add to welfare concerns, such as an “*excessive number of piglets to the sow teat*...*[that] add stress on the system and stress on animals and therefore predispose [them] to illness and use of antimicrobials*” (Interview A). The risk of ineffective antibiotics for treatment and low animal welfare were also said to potentially increase morbidity and mortality in food animals, potentially risking future food security. Good farm practices and biosecurity measures were deemed critical in food production, but non-commercial producers were identified as lacking the finances needed to make the costly investment in biosecurity, which “*forces them a little bit to turn…to antibiotics*” (day 1 workshop). In crop production, it was noted that the high cost of antibiotics compared to pesticides has led crop producers to use antibiotics to grow premium products, such as citrus, and pesticides to grow inexpensive crops such as spinach, which has led to the “*pesticide tsunami*” (day 2 workshop) that can contribute to AMR spread (see theme: Antimicrobial and pesticide use and AMR spread). Government price caps on livestock commodities in some countries and consumer demand were said to also pressure food producers to use antibiotics, fungicides, pesticides, or chemicals to produce inexpensive foods quickly and remain viable in the marketplace.

#### Consumer demands

Participants described consumer demand as strongly influencing AMU in food production. Cultural and religious practices were said to influence the food system. For instance, high consumption of live food-animals and fresh produce, especially during cultural holidays and festivals, were said to amplify the mass and inappropriate use of antimicrobials and pesticides to meet consumer demands. Consumer demand for inexpensive and attractive food was also identified as driving the “*intensive use of pesticides…antimicrobials*” (day 2 workshop) in food production to reduce production costs and grow food quickly. However, growing consumer concern about the safety of the food supply was noted in some SEA countries, creating a demand for hormone-, chemical-, pesticide-, or antibiotic-free foods, and emerging markets for non-meat proteins (e.g., beyond meat). These demands in turn were said to drive market- and industry-led food certifications that could improve antimicrobial and pesticide use practices over time. However, consumer (e.g., individual and farmer) demand for antibiotics was another factor contributing to AMR. A desire to find a quick fix when ill, lack of time to see a doctor, and ability to access or afford health services were some factors identified as influencing consumer demand for antibiotics and inappropriate use.

#### Access to antimicrobials, diagnostics, and alternatives

Ensuring equitable access to antimicrobials was identified as a concern in SEA, and different channels were available for people to access them. Changing health systems featuring increased privatization of hospitals and clinics providing human medical care were said to impact what types of antibiotics people accessed. One participant noted a study where private hospitals and clinics were found to stock and profit from selling expensive, critically important broad-spectrum antibiotics [World Health Organization (WHO), 2019], such as third-generation cephalosporin or a clinic providing IV drips of meropenem to outpatients because “*that is where the profit is*” (Day 1 workshop). Government-funded healthcare facilities, in contrast, were found to stock only cotrimoxazole, an antimicrobial of high (not critical) importance to human medicine [World Health Organization (WHO), 2019]. Over the counter, online and pharmacy sales were said to enable self-medication, access to antibiotics for human use that are used in food animals, and access to legal, illegal, counterfeit, or “*fake*” (interview B) antimicrobials. Access to health services and antibiotic supplies particularly in rural settings were identified as limited and contributing to people’s reliance on “*quacks*” (day 1 workshop) who also may dispense medications of unknown quality or content. All the above was said to enable differential access to quality care and antimicrobials based on ability to access or afford them and inappropriate AMU. Inadequate access to, or availability of, appropriate diagnostics and alternatives to antimicrobials (e.g., vaccines, immunobiotics, and antiseptics) were also said to challenge the accurate diagnosis of clinical symptoms and prescribing practices in the human and animal sectors.

#### Food safety

Food safety practices were said to influence infectious disease, AMU, and resistance. A cultural and religious preference for live animals in SEA and raw food or blood among some populations coupled with potentially unsafe food preparation practices were identified as channels for foodborne illness and hospital visits. Participants also discussed the links between chemicals and AMR in bacterial populations. Antimicrobial compounds (e.g., triclosan, chlorine-based, and ammonia-based agents), “*used a lot and used improperly*” (day 2 workshop) for disinfection on-farms and in the food industry were described to activate resistance genes that, in turn, could potentially transfer to other bacteria and contribute to AMR and also create situations where the chemicals and AROs spread to food, so “*you are not just eating the meat*, *you are eating the detergents*, *the chemicals that come with [it]*” (Day 2 workshop). Increasing automation in food manufacturing was said to decrease the need for human contact with food and use of chemicals to sanitize but has implications for job loss. Less regulatory control over street food stalls and the wet markets that supply them compared to larger food retailers coupled with governments pushing food safety responsibilities downstream through self-regulation rather than investing in labor and structures needed to internalize the “*cost for cleanliness*, *environment*, *health*” (day 2 workshop) in the food production and supply process, were described to negatively impact food safety and responsibility for food safety at all levels (i.e., government to individual), contributing to AMR and its spread. Participants also stressed “*we are still in the dark ages*” (day 2 workshop) regarding food preservation and need to further develop technologies that can produce foods and extend shelf life as a potential direction to reduce AMU, improve food safety and potentially food security.

#### Population growth and migration

Participants described population growth and migration of people from rural to urban areas in search of greater employment opportunities, as creating increased population density, and “*increased demands*, *decreased resources*, *crowding*…” (day 1 workshop), pollution, poor air quality, and waste. These factors, in turn, were said to impact hygiene and increase transmission of infections and bacteria that can enhance the potential for AMU and AMR spread, especially where proper infrastructure and waste management systems are weak or lacking. Movement of animals for cross-border trade was also said to impact infectious disease and AMR spread (see theme: Antimicrobial and pesticide use and AMR spread). Migration was also described to impact the food chain as people moving from rural to urban areas will increase demand for wet market products (see theme: Food safety) and may adopt an “*urban lifestyle*” (day 1 workshop) that changes food demand and consumption patterns and, in turn, nutritional intake and associated impacts (see Overarching factors: Changing socioeconomic structures).

#### Awareness and understanding of AMR

A range of actors’ understanding of AMR was identified as influencing the AMR problem. Authorities, government officers down to producers were said to often lack knowledge of AMR, antibiotics, and which to use when. Participants felt these knowledge gaps contributed to antibiotic use when not indicated and to inappropriate antibiotic use, such as the use of gentamicin for foot and mouth disease by farmers. Small farm producers were noted to lack understanding of good farm management practices. Farmers were also said to trust other farmers and thus the sharing of successes with using an antibiotic was said to motivate others to do the same, potentially creating additional problems with inappropriate AMU. Actors along the food chain, including consumers, were also said to lack understanding about AMR and the importance of animal welfare and food safety practices in the food system. Consumers were also noted to lack understanding about the impact of their food demands on AMU and food waste and their associated negative impacts. Policy makers were described as lacking understanding of vaccine safety (e.g., vaccines for fish), limiting their availability for on-farm use. Prescriber understanding of how to diagnose the type of infection and determine antimicrobial susceptibility, as well as professional norms of physicians, were also said to potentially lead to inappropriate prescriptions such as antibiotics for viral infections in humans as a preventative measure against secondary bacterial infections. These factors were said to influence the risk for infectious diseases, inappropriate AMU, and AMR.

### Overarching factors

#### The drive to survive

The drive to survive underpinned many of the themes and overarching factors identified in this study, creating system-wide impacts that affect demand, supply, and use of antimicrobials and AMR. Participants described a context where countries and people “*resort to desperate measures*” (day 2 workshop) to deal with environmental conditions and poverty while striving toward food security and economic growth. Respiratory infections due to air pollution and diarrhea due to water scarcity in some areas were said to lead to health-seeking behaviors that may lead to inappropriate AMU. Pests, insects, and bacterial diseases that wipe out crops, infectious endemic or zoonotic diseases in food animals and potential pandemics were identified as reducing flock immunity, increasing susceptibility to bacterial infections, AMU, and altering lucrative markets and trade that in turn could impact livelihoods and food security. All these factors were said to drive the “s*urvival of the patient*, *survival of the animal*, *survival of the farmer*, *which can drive you to do whatever you need to put our policies there*” (day 1 workshop). These conditions were also said to drive “*greed*” (day 1 workshop) in pursuit of profit that impacts AMU in food production, such as feed, chemical, and procuring companies and lenders promoting the sale of antimicrobials and chemicals to producers, and government officers “*who sometimes also sell feed*, *chemicals and drugs themselves because that is often their only source of income [as] they cannot charge for their service like… in the West[ern] world [where] your…expertise has a price*” (day 1 workshop).

#### Leadership priorities

Leadership priorities for economic growth and food security in SEA were said to have broad impacts that also affect AMR. In some SEA countries governments were said to increasingly be cutting back on education or health leading to privatization of these and other sectors. This shift from public to private was said to create ripple effects that “*affect other factors*, *making … poor people…poorer*” (day 1 workshop), particularly in rural areas where insufficient opportunities for education or inadequate access to healthcare already exist. Investments in profitable exports that involve the raising of non-native crops and fish in unsuitable environments were said to potentially increase use of limited resources, antibiotics, pesticides, or chemicals to raise the commodity and maximize yields and reduce micronutrients due to lower species diversity or poor soil nutrition. These practices were also said to allow AROs to accumulate in the environment that change its microflora and can spread to people through consumption. Participants also described challenges with investments in food security crops which are often prone to bacterial diseases that may require antibiotic or pesticide use. While ensuring proper micronutrient intakes was identified as important, participants also noted that certain food security crops, such as rice have low nutrient density that can lead to “*hidden hunger*” (day2 workshop), poorer health, and immunity with increased risk of infection in populations where rice comprises most of the diet.

#### Changing socio-economic structures

Although poverty was identified as widespread, participants also described economic growth and increasing wealth in SEA as impacting AMR. Changing socioeconomic structures were said to lead to affluence among some of the population with associated changing lifestyles that change food demands and consumption patterns (e.g., toward Western diets or lab-made non-meat alternatives) that in turn can intensify or lessen pressures for AMU in food production depending on consumer preferences with potential impacts on nutritional intake, the gut microbiome and associated immunity. Increasing affluence and willingness to pay more for farm animal products was also noted to provide an opportunity for the real costs of producing farm animal products to be included in the price, which could facilitate producers to improve their practices (e.g., *via* animal welfare and biosecurity measures) creating indirect impacts that could impact the system. Other changes identified included eating outside the home and overconsumption, leading to foodborne illness risks, rising chronic diseases (i.e., obesity) and corresponding immunity and health, and food waste that can increase resistance and climate-impacting methane production. Changing socioeconomic structures in SEA were also described to drive younger generations away from agriculture to more profitable career paths, leading to higher labor costs to produce food in systems that already rely on antibiotics, chemicals, and pesticides to meet market demands (see theme: Agricultural food production systems).

#### Governance, regulations, and enforcement

Participants described SEA as a region that has concern about AMR, evidenced by governance mechanisms to support AMR mitigation efforts. AMR National Action Plans, international bodies that set AMU standards and trade requirements with national representatives to coordinate implementation, regional platforms that facilitate information sharing and regulatory harmonization between countries, knowledge brokering structures (e.g., regional plant clinics and government officers) that provide advice and technical assistance to farmers, and industry policies and voluntary industry certification programs (e.g., food safety or antibiotic-free food certifications) were said to shape or impact the implementation of policies in SEA, but not without challenges. Inadequately specified policy objectives or implementation plans; insufficient surveillance and monitoring of AMU (e.g., for growth promotion or metaphylaxis treatments), capacity for AMR policy implementation; insufficient numbers of knowledge brokers (e.g., government officers and crop experts) to reach all small farmers; and the presence of siloed government departments (e.g., pharmaceuticals, livestock, and aquaculture) and fragmented engagement of key local stakeholders in some sectors (e.g., aquaculture) were said to limit knowledge exchange and coordination of efforts to achieve targets. Insufficient regulations or enforcement along the pharmaceutical cycle (antimicrobial production, distribution, prescription, dispensing, access, and use) also challenged effective AMR governance. For instance, requirements to establish proof that antibiotics for veterinary use are efficacious were described to be “*relatively less controlled*” (day 2 workshop) than for human use, potentially contributing to AMR selection and spread. In effort to address AMR, some SEA governments were said to have stopped registering antibiotics for use in agriculture (i.e., crops), contributing to black market access to the illegal drugs. Lack of regulations that decouple prescriptions from sales were said to enable “*pervert incentives”* (day 1 workshop) and *“unethical prescribing practices”* (Interview B). *“Relatively unregulated*” (Interview A) distances between land-based farming and watercourses were also described, with implications for AMR spread into the environment. Participants also highlighted how inconsistent regulations and standards and their enforcement between SEA countries, where *“some countries have them*, *some do not*, *some of course a bit*, *some not at all”* (Interview A), add to the problem. These inconsistencies were said to create scenarios where farmers apply good farm practices to meet import requirements of other countries but not for producing domestic foods where enforcement is lacking. Consequently, countries with weaker domestic regulations, surveillance, and enforcement were more likely to have rejected export foods (e.g., due to *E*. *coli* contamination) sold in domestic local markets and use antibiotics, fungicides, pesticides and/or chemicals inappropriately and potentially illegally.

#### Climate change

The changing climate was described as “*affecting everything*” (day 1 workshop). Global warming was said to increase “…*heat stress on animals and farms—pigs and poultry being the most vulnerable—and that will*, *of course*, *increase their susceptibility and sickness and mortality… and then might change the dynamics of endemic disease and other things*” (Interview A). Participants also highlighted how increasing temperatures could increase the occurrence of pests and insects that affect food production. Acid rain due to rising temperatures was also said to become more likely, potentially affecting well water, the growing of crops, and the “*acidity of oceans [and] therefore*, *the so-called microbiome in oceans*” (day 2 workshop) that could impact aquatic life. These forces were said to bolster the drive for survival, increase migration, exacerbate existing food and water security issues, alter the types of foods available and thus food consumption patterns and gut flora, and increase infections, population vulnerabilities and potentially the need for AMU and in turn development of AMR. Participants noted that a greater understanding of how climate change will shape AMR is needed.

#### Underlying intent driving system behavior

Participants described society as a “*very consumptive economy*” (day 1 workshop) due to big business*—*an invisible force that drives current and looming global problems such as AMR and climate change. Participants also identified a lack of systems thinking as exacerbating the AMR problem. “*People aren’t thinking about how to improve the underlying risk factors that drive endemic disease*” (Interview A), which leads to actions that may not address AMR long-term.

### Leverage points

After describing the factors influencing AMR represented as the CLD and overarching factors, participants identified eight CLD factors and two overarching factors as leverage points, places in the system for intervention, along with suggested associated actions. The research team classified each participant-identified leverage point as per Meadows (1999) and then by type (parameters, feedback, design, and intent), which represent “shallow” (*n* = 2) to deep (*n* = 8) leverage points as per Abson et al. (2017). The research team further classified and characterized each leverage point as “shallow” or “deep” in the context of AMR by considering the interventions or actions that participants suggested relevant to a leverage point plus our own understanding of AMR and policy actions to address it (see Tables 3, 4 for a simplified version, Supplementary material F for a detailed version with findings elaborated upon in the discussion section). Overall, participants highlighted that tackling AMR requires targeting interventions at different places that span the One Health spectrum and the need to act quickly, or AMR will be a “*lost cause*” (day 1 workshop).

**Table 3:** Shallow leverage points and suggested associated actions.

*SHALLOW LEVERAGE POINTS: Places for intervention that have less potential to change the entire system’s behavior to mitigate AMR*			
Leverage point for intervention identified by participants	Example suggested intervention or action identified by participants	Type of leverage point and “shallow” or “deep” as per Abson et al. (2017) and leverage point targeted as per Meadows (1999)	Research team’s classification of leverage points as “shallow” or “deep”
National budget, money, funding, and subsidies (*CLD factor*)	Invest in research and development (e.g., developing alternatives to antimicrobials, such as vaccines).	Type: Parameters (Abson et al., 2017); “Shallow” (Abson et al., 2017) Leverage point targeted: *Changing constants*, *parameters*, *and numbers in the system* (Meadows, 1999).	“Shallow”
Resistance in the wider environment (*CLD factor*)	Install green buffers around farms and water bodies to reduce diffuse pollution, accumulation of antimicrobial residues, and AMR spread.	Type: Feedback (Abson et al., 2017); “Shallow” (Abson et al., 2017) Leverage point targeted: *Negative feedbacks* (Meadows, 1999)	“Shallow”

**Table 4:** Deep leverage points and suggested associated actions.

*DEEP LEVERAGE POINTS: Places for intervention that have potential to change system behavior to mitigate AMR*			
Leverage point for intervention identified by participants	Example of an intervention or action identified by participants	Type of leverage point classified as “shallow” or “deep” per Abson et al. (2017) and leverage point targeted per Meadows (1999)	Research team’s classification of leverage point as “shallow” or “deep”
Governance, regulations and enforcement (*Overarching factor*)	• Enforce ban on over the counter antibiotic sales while ensuring equitable access to antibiotics and antibiotic alternatives.	Type: Feedback (Abson et al., 2017); “Shallow” (Abson et al., 2017) Leverage point targeted: *Negative feedbacks* (Meadows, 1999)	“Deep”
Prescribing, diagnosing, treatment practices (*CLD factor*)	• Implement hospital stewardship policies, health professional trainings and audits to ensure quality care and appropriate prescribing practices.	Type: Feedback (Abson et al., 2017); “Shallow” (Abson et al., 2017) Leverage point targeted: *Negative feedbacks* (Meadows, 1999)	“Deep”
Treatment of waste and wastewater (*CLD factor*)	• Improve wastewater management to reduce the accumulation of antimicrobials in city wastewater.	Type: Feedback (Abson et al., 2017); “Shallow” (Abson et al., 2017) Leverage point targeted: *Negative feedbacks* (Meadows, 1999)	“Deep”
Understanding and awareness (*CLD factor*)	• Educate and train food chain actors to implement higher animal welfare systems and biosecurity measures.	Type: Design (Abson et al., 2017); “Deep” (Abson et al., 2017) Leverage point targeted: *The structure of information flows* (Meadows, 1999)	“Deep”
Good farm practices (*CLD factor*)	• Foster multisectoral collaborations to share knowledge and resources that improve farm practices.	Type: Design (Abson et al., 2017); “Deep” (Abson et al., 2017) Leverage point targeted: *Add*, *change*, *evolve or self-organize system structure* (Meadows, 1999)	“Deeper”
Development, access, and availability of alternatives to antimicrobials (*CLD factor*)	• Disseminate and use autogenous vaccines (e.g., for aquatic animals) made with local pathogens.	Type: Design (Abson et al., 2017); “Deep” (Abson et al., 2017) Leverage point targeted: *Add*, *change*, *evolve*, *or self-organize system structure* (Meadows, 1999)	“Deeper”
Research, development and innovation (*CLD factor*)	• Research crop ecosystems and microflora and develop narrow spectrum antibiotics for crops.	Type: Design (Abson et al., 2017); “Deep” (Abson et al., 2017) Leverage point targeted: *Add*, *change*, *evolve*, *or self-organize system structure* (Meadows, 1999)	“Deeper”
Underlying intent driving the system (*Overarching factor*)	• Ensure global collaboration to change the underlying “*consumptive economy*” (day 2 workshop) that drives system behavior and AMR.	Type: Intent (Abson et al., 2017); “One of the deepest” (Abson et al., 2017) Leverage point targeted: *The paradigm out of which the system arises* (Meadows, 1999)	One of the “deepest”

## Discussion

To our knowledge, this is the first study to identify and visually illustrate in a CLD a broad range of factors that may impact AMR in the SEA food system and identify places for intervention with potential to mitigate AMR. By applying a participatory One Health approach and systems lens, we were able to integrate the perspectives of stakeholders relevant to human, animal, agricultural food, and the environment sectors into two CLDs that were ultimately combined into one due to similarities. While workshop and interview discussions touched on similar themes, some topics were emphasized more depending on workshop day or interview. Day two workshop participants were more likely to discuss in greater detail issues relating the food insecurity, nutrition, and the microbiome, consumer demand, and food waste, with a Malaysian lens as most participants lived and worked there. Day 1 workshop participants and interviewees were more likely to focus on livestock and aquaculture production systems, good farm practices, animal welfare, and healthcare with a SEA regional lens as most participants lived or had experience working in different SEA countries or at the regional level. Thus, by bringing together diverse stakeholders, we were able to identify a broad range of factors and deepen understanding of how actions in particular sectors may influence AMR.

Many factors identified by participants in our study echo what has been found in the literature. Inappropriate AMU in agricultural food production and in healthcare and the community was identified as the major driver of AMR. Antibiotic and AMR spread through the food chain, into the environment, to wildlife and humans were also identified as driving AMR as others have found (Bondad-Reantaso et al., 2012; Holmes et al., 2016; Stålsby Lundborg and Tamhankar, 2017; Zellweger et al., 2017; Ng et al., 2018). Consistent with others (Gupta, 2012; Laohaudomchok et al., 2021), our study found pesticide use in food production in SEA, which can contribute to AMR *via* the natural environment (e.g., soil; Malagón-Rojas et al., 2020; Miller et al., 2022), reinforcing calls to better understand how soil, water, and pesticides interrelate to generate and spread AMR (Miller et al., 2022). Other participants identified social factors influencing AMR in SEA which are also found in the literature, and included: lack of awareness about AMR, antimicrobials and their proper use among the public, food service industry, prescribers, drug sellers, farmers, and knowledge brokers, such as government officers; inappropriate prescribing practices; and access to antibiotics or antimicrobials through public, private, and unregulated supply chains (Puspitasari et al., 2011; Islahudin et al., 2014; Nga et al., 2014; Om et al., 2017; Zellweger et al., 2017). Our SEA participants also articulated how culture and religion influence food demands that drive AMU and prescribing practices. The need to better understand and account for the role of dynamic cultural contexts when addressing AMR has been previously raised (Ledingham et al., 2019). Links between food waste and AMR were also identified, an area that has previously been recognized as warranting quantification of risks to human health (Furukawa et al., 2018; He et al., 2019). Many of the participant identified factors have also been found in other low- and middle-income, as well as high-income, contexts (Cabello et al., 2016; Iskandar et al., 2020). Six additional overarching factors that can potentially exert broad impacts on the SEA system were highlighted. Four of these overarching factors have been discussed to some extent in the literature and warrant continued examination in terms of their impacts relevant to AMR: leadership priorities; governance, regulations, and enforcement (Collignon et al., 2015; Chereau et al., 2017; Goutard et al., 2017); socio-economic forces (Collignon et al., 2018), and climate change, such as the impact of environmental stress or changes on bacteria and AMR (Burnham, 2021). Furthermore, there is a need to better understand and account for the impacts of the remaining two overarching factors on AMR: the drive for survival due to poverty and challenging environmental conditions; and the underlying intent that drives how society and businesses operate (i.e., accruing profit and wealth for big business), which can, for instance, create scenarios where small profit margins and limited investment interest disallow many farmers in low- and middle-income countries access to technological advancements that might help mitigate AMU, such as vaccines, biosecurity, and genetic improvements.

Our study combined the participant-identified factors in one CLD to illustrate how they interact and broaden our understanding of AMR emergence, spread, and impact in SEA. This model and participants associated discussions highlight that AMR emerges through actions taken in different parts of the One Health spectrum in different SEA countries and that AMR exerts health, environmental, and economic impacts. Model complexity points to a need for multi-level governance mechanisms and a whole of government and One Health approach to enable cross-sector collaboration and the development and implementation of coordinated actions tailored to the SEA context to mitigate AMR [Kickbusch and Gleicher, 2012; Kakkar et al., 2018; Interagency Coordination Group (IACG) on Antimicrobial Resistance, 2019]. SEA has governance mechanisms to support AMR. Regional level mechanisms relevant to AMR exist [World Organisation for Animal Health (OIE), 2022], and despite challenges with implementation, many SEA countries have AMR National Action Plans (Qijia Chua et al., 2021). The region has also invested decades in fostering better regulation reforms that have been identified as important to leverage knowledge and identify leverage points for intervention that can improve country responses to complex problems such as the COVID-19 pandemic (OECD, 2021) and likely AMR.

### Leverage points for intervention

Our study found two “shallow” and eight “deep” leverage points in the system. These places for intervention suggested by participants targeted different parts of the system in effort to transform AMU practices, and limit AMR development or spread. Shallow leverage points (Abson et al., 2017) do not change the behavior of the system and yet are often targets for policy intervention (Meadows, 1999; Abson et al., 2017). Shallow leverage points in our study focused on changing constants, parameters, and numbers (Meadows, 1999) by directing “national budgets money, funding, and subsidies” to different areas (e.g., training of health professionals and other prescribers on antimicrobial stewardship and into research and development). Another shallow leverage point focused on introducing negative feedback into the system to keep a system state in balance. Here, participants identified a need to limit “resistance in the wider environment” *via* the installation of green buffers to reduce the spread of pollution, antibiotics, and resistant organisms in waterways. While directing money and instituting actions such as green buffers to help limit AMR are important, money flows may be time limited and green buffers may only protect the areas in which they are embedded. Moreover, these leverage points are typically considered less likely to change the behavior of the whole system unless accompanied by changes in deeper leverage points (Meadows, 1999; Abson et al., 2017).

Different deep leverage points were found in our study. Deep leverage points (Abson et al., 2017) are often harder to implement but have greater potential to change the behavior of the whole system (Meadows, 1999; Abson et al., 2017). One deep leverage point focused on introducing negative feedback loops (Meadows, 1999) to control or keep system states within safe bounds through changes in: “governance, regulations and enforcement”; and “prescribing, diagnosing and treatment practices” (e.g., by setting or strengthening and enforcing regulations to control the production, supply, demand, and use of antimicrobials relevant to the food system). Negative feedbacks are typically considered a shallow leverage point (Abson et al., 2017). However, we categorized these as a deep leverage point because the interventions or actions participants identified as needing regulations and enforcement targeted different parts relevant to the food and healthcare systems that could change practices embedded in organizations, institutions, and the public, and if all participant-suggested interventions were implemented, it could potentially catalyze a change in system behavior in ways that may help mitigate AMR. Another deep leverage point focused on changing the structure of material stocks and flow (Meadows, 1999) by focusing on the “treatment of waste and wastewater” (e.g., improving infrastructure standards in treatment plants to reduce antimicrobial residues and AMR accumulation and spread). While considered a shallow leverage point (Abson et al., 2017), we assert that interventions that seek to change the structure of material stocks and flows *via* targeting the treatment of waste, wastewater, and water represents a deep leverage point due to its potential to impact the whole system by improving drinking water, sanitation, and hygiene and, in turn, significantly reducing infectious diseases and strengthening health and well-being. These actions are also deemed a key action area for addressing AMR and the Sustainable Development Goals [World Health Organization, 2020a,b; World Health Organization (WHO), 2021]. The challenge in creating these changes, however, may be the cost and time it takes to bring water and sanitation treatment service to needed levels including in areas where resources and access to adequate clean water is lacking. Another deep leverage point focused on changing the structure of information flows in the system (Meadows, 1999; Abson et al., 2017) to increase “understanding and awareness” about issues relevant to AMR in SEA. This leverage point focused on implementing new or using existing channels to deliver information about the consequences of actions to places in the system where it is missing as it could cause people to behave differently (Meadows, 1999). While participants recognized that delivering information is insufficient on its own to change behavior, they also stressed that improvements in current levels of awareness in SEA would be highly beneficial. Conducting campaigns that aim to persuade consumers to see AMR as a social responsibility was one intervention participants identified to increase consumer understanding and potentially change their demands and behaviors. Using existing channels by training, for instance, government officers on AMR, good farm practices, and AMU, who then go on to educate and train farmers was another means participants identified for delivering information to places where needed. Delivering information through the media was another channel for delivering information across the system and thus, participants identified a need to engage and train the media on AMR issues. Another even deeper leverage point that aimed to change how the system is designed (Abson et al., 2017) focused on the power to add, change, or self-organize system structure (Meadows, 1999) by targeting changes in: “good farm practices”; “research, development, and innovation”; and “development, access and availability of alternatives to antimicrobials.” Here, participants identified the need for multisectoral collaborations and research and experimentation to enable the sharing and integration of diverse perspectives, knowledge, and resources that can inform the development of policies, actions, and technologies that, in turn, could help build SEA’s capacity or resilience to address AMR. The deepest leverage point identified, however, targeted changes in the paradigm or mindset out of which the system arises (Meadows, 1999; Abson et al., 2017) by shifting the “underlying intent of the system” away from the profit-driven mindset that drives system behavior and was deemed by participants to be a key driver of AMU, to one that prioritizes health and relies on global collaboration and systems thinking to determine and address root causes of disease and AMR, such as poverty, sanitation, and food insecurity. Targeting deep leverage points for intervention is important because they influence what interventions are put in place at less deep and shallower leverage points (Abson et al., 2017). Changing the intent of the system is considered the most challenging to achieve, yet success would catalyze aligned changes in previously discussed leverage points, altering the behavior of the entire system (Meadows, 1999) in ways that could sustainably mitigate AMR. At the same time, shallow leverage points for intervention, such as those that aim to change constants and parameters in the system may help increase attention to, and understanding of, the AMR problem. These changes could, in turn, help shift mindsets, and catalyze changes in deeper leverage points. Greater understanding of how deep and shallow leverage points influence one another has been identified as needed to deepen our understanding of how to sustainably address complex problems (Abson et al., 2017) like AMR. Understanding that there may be perceptions or real differences or trade-offs between the actionability of shallow leverage points and broader less actionable deeper leverage points for which outcomes or impact might be more difficult to evaluate is also important when examining where to target interventions.

### Determining feedback mechanisms

The success of the above leverage points in achieving sustainable change depends on whether they are part of feedback loops that help or hinder AMR mitigation. Our CLD revealed 98 factors interlinked by 362 connections and six overarching factors that generate and spread AMR. Given the complexity of this densely interconnected structure, it is highly likely that identified leverage points fall on multiple feedback loops, increasing the potential for interventions to create unpredictable consequences (positive or negative) that can affect the sustainability and effectiveness of interventions to tackle AMR. For instance, implementing negative feedbacks such as taxing red meat could limit access to nutritious and affordable food among vulnerable population groups who cannot afford them, alter what other consumers purchase, and potentially push some producers out of business. Future research to elucidate feedback mechanisms in our CLD is necessary to determine which leverage points have the potential to desirably change system behavior without creating chaos and major unintended consequences. This underlines the need for a learning system that documents interventions, their implementation, and impacts in specific contexts (Wernli et al., 2020).

### Strengths and limitations

Our study successfully obtained representation of actors (researchers and implementers of policies and programs) across the One Health spectrum from primarily Malaysia and also Thailand, Indonesia, Singapore, Laos, Sri Lanka, Ethiopia, and India, all with experience conducting work relevant to the SEA context. However, we did not secure representation from all SEA countries, and lacked certain perspectives at the table (e.g., national governments and consumer advocates), thus factors unique to other parts of the SEA and particular sectors may not have been fully captured even though our participants did discuss factors impacting the region. Also, most of our participants were male and native to SEA, yet we had few female participants, and they were not native to SEA. Since gender differences in AMR knowledge and practices exist (Pham-Duc and Sriparamananthan, 2021), future research would benefit to include more females native to SEA to further broaden understanding of gender-related influences on AMR and determine tailored intervention needs.

Another study strength is that our participatory approach successfully yielded a CLD that illustrates the underlying causal system of factors relevant to the SEA food system, a tool that interventionists and decision-makers can use to understand AMR complexities, explore how interventions might impact system behavior, and determine how to address potentially undesirable consequences. While our participants validated our CLD model *via* feedback, we did not verify statements made by participants against existing literature nor did participants identify the relative importance of factors in the SEA context, which warrant future investigation.

## Conclusion

Utilizing a social-ecological systems lens, our study created a CLD that illustrates numerous interconnecting factors that influence AMR spanning the One Health spectrum relevant to the SEA food system, plus overarching factors with broad impacts on this system. This captures the complexity of the AMR problem and challenges with determining how best to intervene. Our study identified several leverage points for intervention across human, animal and environment sectors that if comprehensively targeted with multi-pronged interventions may have potential to change how the system behaviors in ways that help to mitigate AMR. Even targeting some of the identified leverage points for intervention, such as increasing national budgets toward research and AMR-related capacity building, and setting and enforcing regulations to control the supply, demand and use of antimicrobials may help shift mindsets. These actions, in turn, may support actions that enact more difficult to change leverage points, such as redefining the profit-driven intent that drives how society and businesses operate toward one that values health, and in turn transform AMU and limit AMR, building SEA’s resilience to address AMR.

## Data availability statement

The original contributions presented in the study are included in the article/Supplementary material; further inquiries can be directed to the corresponding author.

## Ethics statement

This study involving human participants was reviewed an approved by a University of Waterloo Ethics Committee, Office of Research Ethics, University of Waterloo (# 40159). The patients/participants provided their written informed consent to participate in this study.

## Author contributions

SEM, EJP, SH, PSJ, and DW: conceptualization of the study. IAL, MC, EJP, CAC, SEM, TG, AL, DW, PSJ, PJGH, MT: method development. IAL, MVC, MC, EJP, CAC, SEM, TG, AL, DW, PSJ, PJGH, MT: data collection and analysis. SEM, EJP, CAC, SH, PSJ, DW, TG, AL, PJGH, SH: funding acquisition. IL: manuscript writing. All authors contributed to the article and approved the submitted version.

## Funding

This study is funded through an operating grant of the fifth Joint Programming Initiative on Antimicrobial Resistance (JPIAMR 2017). Funding was provided by an operating grant from the Canadian Institutes for Health Research (Institute of Infection and Immunity, Institute of Population and Public Health, grant number 155210, PI SEM), a Swedish Research Council grant (grant number 2017-05981, PI and Project Coordinator PSJ); and an operating grant from the Swiss National Science Foundation (grant number 40AR40_180189, PI DW). The funders had no role in the design analysis or writing of this article. MVC was supported by the CGIAR Research Program on Fish Agri-Food Systems (FISH) led by WorldFish and the One CGIAR Initiative “Protecting human health through a One Health approach.” PJGH and MT are partially funded by the FORMAS SeaWin project (2016-00227), and PJGH is also partially funded by the FORMAS Inequality and the Biosphere project (2020-00454). MT and PJGH undertook this work as part of the CGIAR Research Programs on Fish Agri-Food Systems (FISH) led by WorldFish and on Climate Change, Agriculture and Food Security (CCAFS) led by CIAT.

## Conflict of interest

The authors declare that the research was conducted in the absence of any commercial or financial relationships that could be construed as a potential conflict of interest.

## Publisher’s note

All claims expressed in this article are solely those of the authors and do not necessarily represent those of their affiliated organizations, or those of the publisher, the editors and the reviewers. Any product that may be evaluated in this article, or claim that may be made by its manufacturer, is not guaranteed or endorsed by the publisher.

## Abbreviations

SEA: South East Asia/South East Asian
ARO(s): Antimicrobial resistance organism(s)
AMR: Antimicrobial resistance
AMU: Antimicrobial use
CLD: Causal loop diagram(s)
