# Supplementary material for: Factors impacting antimicrobial resistance in the South East Asian food system and potential places to intervene: A participatory, one health study
Source: Front Microbiol. 2023 Jan 5;13:992507. doi: 10.3389/fmicb.2022.992507 (PMC9849958; doi:10.3389/fmicb.2022.992507)
Supplement: Supplementary file 1 [file Data_Sheet_1.zip › Supplementary File A.pdf]

SUPPLEMENTARY FILE A: QUOTES PER THEME

Acronyms:

P: Participant
R: Researchers that facilitated discussions
SEA = South East Asia
AMU = Antimicrobial use
AMR = Antimicrobial resistance

Please note:

1. Some quotes were modified in instances to protect the identity of participants or the organizations they represent.
2. P: represent a participant. Some quotes contain multiple P's. This means the quote reflects a discussion thread involving a participant and the researcher or multiple participants.
3. A given quote may be coded in more than one theme if it applies to each.

THEME: Antimicrobial and pesticide use and AMR spread

**Antimicrobial and pesticide use and AMR spread:**<br><br>How antibiotics, pesticides, fungicides and chemicals/detergents are used (and contribute to AMR and/or AMR spread).	Day 1 workshop:<br><br>P: One more thing which is missing on this list is the use of agriculture by [inaudible] for feed manufacture<br><br>P: As a free resource used.<br><br>R: in production… [some discussion inaudible]<br><br>P: using agriculture products and by-products [R: agricultural by-products] yea, by-products, especially by-products.<br><br>R: yea, these by-products from agriculture, you put it in the feed…which then get fed to the animals…
	Day 1 workshop:<br><br>P: Well the question is the quality control of feed, because a lot of antibiotics are being mixed into the feed. Some on purpose and some people that know they are in there, but they just makes them feed. Or some say there are all sorts of things in there, and it's not, it could also be that [inaudible] trenches …
	Interview A:<br><br>P: …smallholder / contract farmers may use cheap antibiotics as a form of 'insurance'.

	Day 1 workshop:<br><br>P: Regarding antibiotics should be using for the livestock. I will give you an example. Since last year [name of a SEA country] ban for ATP, but I just attended [inaudible] the private sector company last month, even though we ban ATP in [name of the SEA country that banned the antibiotic] but the antibiotics still high, they still put it in livestock. Because the farmer worry, because they worry… how to protect the farm.
	Day 2 workshop:<br><br>P: Prudent antibiotic use on poultry farms: double dose antibiotics use, no antibiotic rotation, no correct dose, no correct application.
	Day 2 workshop:<br>P: Critically Important Antimicrobials (CIA) for human medicine used for poultry farms such us: colistin and ciprofloxacin
	Day 2 workshop:<br><br>P: …So because based on my experience that when we are collecting the samples, okay, from a farm level, so we always heard about where from the farmers they say they tend to use a lot of, drugs or antimicrobials instead of just for preventive purpose. So even they use prohibited drug, okay, even they use a prohibited drug…for a number of agriculture…<br><br>R: Okay<br><br>P: Yea, because we don't have a good regulatory framework to control.
	Interview A:<br><br>R: … and you mentioned metaphylaxis…Is metaphylaxis its own node?<br><br>P: Yes, I think so.<br><br>R: Okay.<br><br>P: So perhaps you know having two that, the prevention of you know clinical and confirmed infections and useful metaphylaxis, and we know that that is happening in a wide scale in many industries.<br><br>R: And is metaphylaxis…are the regulations around, like what is the sort of rule generally?<br><br>P: Yea, I know [name of SEA country] does, but I can also talk a little bit to [the names of 3 other SEA countries]. At this stage as you probably know, there are attempts or you know use or in some countries regulations around metaphylaxis in relation to antibiotics I might add. Not everything, but antibiotics used in feed and water. So in [name of SEA country] that

	exists, so they can be used under veterinary prescription, but it may not exist for every country. It does in theory exist I believe in [name of another SEA country], but it is still used. So there is a lot of undercurrents you know, illegal use also, because either it is broadly done under a veterinary provision, or it is just done illegally with mass use, and I know that because I was speaking to a [person from name of a SEA country] vet just the other day. So because it is not necessarily good enforcement in many of these markets, but the other area is - now in terms of general metaphylaxis, there is beyond feed and water use, which is usually the routes to metaphylaxis obviously, but there is not necessarily and the metaphylaxis and the feed and water use is usually restricted to antibiotics. It is not necessarily for anti-toxicity stats, or those antibiotics that are not considered critically important. So …other types of antibiotics, tetracycline obviously, amoxicillin and others.
	Interview A:<br><br>P: And it is a big, still a big, large remaining use of antimicrobials into the system, and so for example in [name of SEA country] they can't use in theory legally certain antibiotics in feed or water, and unless prescribed for a vet. Now that would mean that they can't, okay just use them routinely off the [inaudible] promotion. However, if the vet agrees that there is a high risk perhaps of a certain disease, whether it be viral or bacterial, then they might prescribe that it can be used to prevent sort of a rise of say a bacterial related you know respiratory disease or gastrointestinal disease for example, and so I think splitting those two out is quite important because there is a lot of nuances around on the farm use in the system. So I perhaps consider yet parallel and they both come back to animal welfare. They both come back. They might link to growth promotion, so they might get a by-product benefit certainly. I think in using it for metaphylaxis, but actually also inherently provides a growth promotion aspect as well.
	Day 2 workshop:<br><br>P: The second part I wanted to make is in terms of you are not treating, and especially in that aquaculture, let's say we are not using the antibiotics, so we have a situation where we don't know the entire population is sick or only a few are sick. [R: yea] Right, so we have those based on the mass, per KG or something like that, the dose is given, but there is a possibility that maybe 10% are sick. You know 90% are healthy like for example. So, if you see the mechanical direction of antibiotics, antibiotics are used by the organisms which are infecting organisms, thinking that they are nutrient, you know nutrients, and it gets used them and then end up in not having the necessary nutrition or probably might end up in some metabolite which is detrimental to their you know multiplication. So supposing you have only 10% or 20% of them are really sick. The remaining are not sick, but all of them will eat the antibiotic for example. So what will happen with the antibiotic, whether you know it is

	consumed or it remains unconsumed, for example? So that also you know would lead to probably some of the antibiotic residue getting into the humans possibly.<br><br>R: So would we phrase that as decreasing some of this prophylactic use? Now… [P started talking, inaudible]<br><br>P: Yes, that is, preventive action is different, but even in the case that you have a situation, that you know you have a disease that is already, you know, there is a disease, so we really don't whether all of them are affected or some of them not affected, some of them are affected, but it is difficult to make up.<br><br>R: Yea. In Europe they call that metaphylactic use where part of the herd is infected and part is not.<br><br>P: Yea.<br><br>R: So maybe that decreases this particular use, where it is not known if all the population are sick.<br><br>P: Yea, that is right, but as in the case of maybe in the case of piggery, or in the case of chicken, poultries, probably we could isolate those which are sick and then you know we could, you could treat them, whether those which are not you know.<br><br>R: So if we improve isolation of sick animals, we will have less disease and less resistance, and less use.<br><br>P: Yea.<br><br>R: Yea, and this use practice often happens in herds or like flocks where are multiple animals and yea, or in aquaculture where there is a pen.<br><br>P: Yes.<br><br>R: …Is this how it works in Southeast Asia? …on the animal side?<br><br>P: I am not sure. Generally speaking, I am not sure. [R: that's fine] But basically it ends up you know what happens to whether we can have an antibiotic that you know those have been consumed, or it has not been consumed. If at all it still remained unconsumed, so what is the impact of all that. If you stay, you can be done by, you know, some other… so you might end up having some resistance.
	Day 2 workshop:<br><br>P: … now I was in the pesticides point for many years. So I look at this original data, the distribute data, I would say about 30% of our vegetables

	get rejected on a monthly basis, but that is because of fungicides, you know, because fungicides have very low PR intervals, and so therefore you know your PPM levels are very low. So therefore, it gets rejected very fast. The reason being many vegetables because by the time you harvest and you bring them to market, it takes some time and fungicides prevent post-harvest, you know, harvest, fungicides prevent deterioration, otherwise farmers lose a lot. So that is the reason why they spray…
	Day 2 workshop:<br><br>P: Basically the issue is not so much the antibiotic use. It is more on the fungicides and insecticides.<br><br>P: Because fungicide also in the end, okay, they also will affect the AMR as well, yea, [another P: we seldom analyze] because actually it is quite high in… because we always collect samples from the retail level, we found actually there is a trend. Okay, this is a trend of the AMR, because in the past few years, we found that actually tetracycline is no longer to treat the listeriosis, which cause by listeria monocytogenes. but this is a change [inaudible] today, okay, but after a few years back, okay, so we collect the samples again, we found, "oh, tetracycline is effective against", so we believe there is always a cycle, okay. So… what am I going to say… because we found there is normally, okay, because if the agriculture practices especially like the commissioner okay, commission and produce, we found that especially in [name of place] or especially in the industrial scale of the agriculture productions, definitely the AMR of the trace very high. Okay. Apparently this is caused by environmental [inaudible], spreading of the bacteria from the other farms. So and also because of the increased use of pesticide and also fungicide perhaps that is one of them, since that cause the AMR problems.
	Interview A:<br><br>The only other thing perhaps and I guess you have got it here under eggs is with poultry and as you say, you have got restocking animals, high risk for infection, but obviously routine use of antibiotics and other antimicrobials for eggs to prevent salmonella.
	Day 1 workshop:<br><br>P5: Yea, several other things we should talk about. So we were talking about at lunch time, I was touching on was cross over management of human infections and the fact that we have a limited number of antibiotics and a lot of infections to treat. So antibiotics get used for different infections and something like TB [tuberculosis] is a good example, to treat an infection you need to treat for a long time and you will be using antibiotics which can be used for other common infections as well, like and inevitably within the human that you are treating for TB you are potentially generating resistance to those agents and they become focus for dissemination. Now that is just inevitable. It is very

	difficult to do much about that. We accept that that changes the flora, one of the nodes you have got there are already changes the gut flora in those patients, not just gut flora but that is an important example.
	Day 2 workshop:<br><br>P: The other thing is of course now extended use of soft approaches, chemicals like [inaudible] which is now coming into, it had been used for quite some time, but people are bringing transgenic species, [inaudible] canola in [name of a non-SEA country]. All these things are also causing problems in terms of genes floating over across systems… I think it is a big issue now, because a lot of crisper-based, you know this conjugated, this gene modification going on, slicing and splicing and changing efficacy. Yea.
**Antimicrobial and pesticide use and AMR spread:**<br><br>Influence of effluence, water, wastewater, recycling of waste and wastewater in agriculture, trade, transport, travel, health care settings on AMR.	Interview A:<br><br>P: …and where it needs more research or not is arguably but there is always a focus on alternatives, including probiotics, which as you know probably there is antimicrobial resistance now being linked to probiotics, there is antimicrobials resistance being linked to disinfections, exiting iodine and others, and even antimicrobial genes or things found in genetically engineered animals, as well as, yea, so you know the ability for these plasmid to move through the systems, extremely efficiently and effectively as you know is extraordinary, so that relates definitely to the environment…
	Interview A:<br><br>P: It is interesting to note (perhaps with the OIE focal point mentions) the OIE veterinary list of antibiotics is almost an inversion of the WHO CIA list – yet transference of resistance is clearly shown via plasmids, DNA etc and even to probiotics, some disinfectants.
	Day 1 workshop:<br><br>P: Basically it's effluent pollution.<br><br>R: The industry, the pharmaceutical industry point that you said, we haven't captured what that was. Yea, I am wondering how to call it. So run off? Waste?<br><br>R: Contaminated effluence.<br><br>P: Effluence, pollution and waste are the three key words there. [R: what is it?] effluence, pollution in the broader term.<br><br>R: Okay.<br><br>P: So a lot of this is captured somehow here in resistance entering the wider environment through manure run off or waste water, but it aggregates so much that maybe it deserve disaggregation.

	Interview A:<br><br>P: So thermal comfort, aspects of concentrated diets and feeding, competition, and all aspects of stress, etc. etc. So that is really the key for us and that also definitely relates to aquaculture, of course as well, and with a number of factors there, and of course relates to the environmental, so obviously prophylactic, any use of antibiotics as well, because they know whether through illness or predominately for I guess you have got on farm use there as that node, terrestrial node that relates to, directly to run off…
	Day 1 workshop:<br><br>P: But I think again here, the thing to do is the [inaudible]. From a systems thinking perspective, [inaudible] treat sectors different than other programs end up with problems. Take it, for example, [name of a SEA country], [inaudible] take a landscape approach, [inaudible] technical [inaudible] performing area, you have rice, you have fish, you have livestock, you have good meat, you also have a connected food system. So there everything is happening. Excess use of pesticides and chemicals in the fields going into the water, human waste mixed gets into the water, water is to fish, so there is the constant movement, so it is not only the AMR going from fish to humans, it could be AMR going from humans to fish. [Inaudible] we could have an endemic disease, lots of treatment and all of humans are affected and low purification, seaweed contamination and poor farming and fish. Fish could be getting left from the humans. [Another P spoke but inaudible.] So I mean… Water is important, water is the medium. Whoever is using the water can be contributed to the list.<br><br>[Another P: yea]<br><br>P: Can be exposed to the list.
	Day 1 workshop:<br><br>P: This is where I think you need to consider [inaudible] industrial farming… that I can [inaudible] in …other [non SEA] places…how a single landscape is used. [inaudible] Landscape is exclusive for tree, or exclusive for [inaudible]…where [in name of three different SEA countries], the landscape is used for multiple purposes.<br><br>P2: This farming is quite low cost.
	Interview A:<br><br>P: In some ways this increasing use of certainly by the big companies, so can afford to, to use biogas units on their, particularly on their peak units, but the effluent ultimately the manure must go somewhere. Location of farms is a classic thing that probably isn't yet, is a high risk factor. For example, obviously in dairy, but certainly with pig farms, probably poultry as well, proximity to water courses, the water table, lakes, etc. etc. is

	increasingly important, and that is relatively unregulated I believe in Southeast Asia. So regulation in relation to farm … and the environment. I don't know if it is already captured in non-farm environmental areas, but…<br><br>… as you probably know run off from dairy farms is increasingly controversial into the waterways, and dairy and particularly young dairy of course is a high risk industry for antimicrobial use in AMR. I don't know as much about, I know a little bit about dairy in this region but not a huge amount, but it is not, in [name of two SEA countries], it is formalizing as effective, but not nearly as much as say pigs and poultry, but antimicrobials in calves, this is obviously a high use and even still in [name of a region of the world], so yea I do think that the environment one might need a bit more teasing out.
	Day 1 workshop:<br><br>P: Not only these, not only the all the forms being [inaudible]… [background chattering continued]… there are other connections. Probably many of them is routinely used as a fertilizer in aquatic systems to increase production of [inaudible]. But when it was a major source risk factor for entry of point-source [inaudible] could be residue of antibiotics use. There is a direct connection.
	Day 2 workshop:<br><br>P: …And farmer markets, and a lot of them are contaminated with e-coli and that kind of stuff, because people use all kinds of organic manure.
	Interview A:<br><br>P: Related and not explicitly covered – the importance of direct contamination of the environment – in excreted antimicrobials, especially where up to 90% of antibiotics may be excreted on or from farms to the environment, directly or after crop spreading, effluent digesters etc….
	Day 1 workshop:<br><br>P: In the centre we have here resistance entering the wider environment.<br><br>R: Yea.<br><br>P: So I would say that we have on one hand antibiotic resistant bacteria entering the environment and antibiotics themselves entering the environment and then the environment could be mainly water and soils, and then antibiotics can get into water and then again on the top you have human antimicrobial use, and this is typically you know the prescriptions you get from your doctor, etc. but you can also get exposed, you can get exposed to antibiotics through drinking water.

	R: Okay.<br><br>P: So you see the connection.<br><br>R: Yea.<br><br>P: So it is water with antibiotics and then drinking water and then exposure to humans. Then we have I believe antimicrobial system bacteria excreted by humans that end up in water and then there should be also an arrow linking this to resistant human infections. So because you know there is another exposure pathway for humans to get exposed to antibiotic resistant bacteria through water. Not only<br>…<br><br>P: But the same with …the AMR.<br><br>R: Should we break it apart from like water and soil?<br><br>P: So to me I would say, divide environment in water and soil and then for water you get antimicrobials getting into water and then again people getting exposed to antimicrobials through drinking water is one thing. Then you have also people getting exposed to antimicrobial resistant bacteria through water. So there is another arrow that connects water through resistant human infection to resistant human infections.<br><br>R: I did already do that one.<br><br>P: Okay and the same with the rest. The same with all water users as she said, but it is already linked and fish so aquaculture…to antimicrobial use.<br><br>P: Connect environment back to humans.
	Day 2 workshop:<br><br>P: If we assume that the water is one of the vehicles, of the channel, to a spread of antibiotics and antibiotic resistant bacteria, then what can we do to prevent water pollution by antibiotics and antimicrobials, so where is water treatment would be one of the things to consider and the ones, water is already polluted with antibiotics, and antibiotics drinking water treatment,
	Day 2 workshop:<br><br>P: Yea, so the way that the wastewater comes from farms is manure, the wastewater.<br><br>R: So we have, this is entering the water environment, I do have water treatment here. Should I be extending that to waste treatment, like just general treatment, waste treatment or do a separate node for other kinds of waste?

	P: So when we talk about resistance entering the wider environment…<br><br>R: I have on farm pollution control going in here. Is that true?<br><br>R: Yea.<br><br>R: Did you get that one? [group laughter] Is that good I captured that part? I think you have got another part for the system too.<br><br>P: Well so by environment we can have water or soil, so the same will happen that applies to soils, but typically wastewater is discharged to water, not to soil, but manure is many times reused in soils, so treating the manure before being reused in soils will be important for preventing this manure to be polluted with antibiotics or antibiotics resistant bacteria, no but that would be because we have runoff here, wastewater, you know these are the pathways by which antibiotics get into water and the soil.
	Interview A:<br><br>P: I am just trying to see what the relationship between on farm use and entering the environment, when you have run off wastewater. Obviously I think you had that linking to resistance in food products and crops, yea so that makes sense.
	Day 2 workshop:<br><br>P: Yea, well in developing countries wastewater treatment you know it is very, very rare, effective water treatment is very rare. So all these wastes get into waters untreated.
	Day 1 workshop:<br><br>R: Because we actually in our rabies vaccination, raccoons, we use antimicrobial [inaudible]so it is …<br><br>P: Actually in countries like [name of country], we have [inaudible] about the [inaudible] sometimes our area is exposed to [inaudible] some people<br><br>R: That's an important thing we don't have. So would it be [R: human waste?] uncontrolled access to, I don't have the right words to cover it, because I am going to… resistance in water, but also sometimes it is livestock too.<br><br>P: Yea like livestock, and even animals like monkeys.<br><br>R: Right. What would be a good way to phrase that then? It is almost like, so what would be a good way to describe situations where livestock, wildlife have access to waste? In Canada our waste facilities are quite contained. It doesn't mean that birds, raccoons, skunks, etc. don't get in,

	but I mean have seen situations in [name of country] where there were pigs eating in the garbage dumps. So how do we say that?<br><br>P: Waste management in general? Waste management? Yea.<br><br>P: By livestock and… accessing the waste. Sometimes it's not just waste, it's also…[inaudible]<br><br>P: Like little sanitary [waste] …<br>…<br>P: Because then that is an important connecting point of human, animals, especially in livestock exposed to risk, resistance
	Interview A:<br><br>P: … but is there a link with on farm use, and farm workers, who are then exposed? So farm workers, because obviously run off I think that is accounted to the environment, which is a significant and increasing focus in relation to AMR, but farm workers, veterinarians, and their families, interrelated, there is a direct relationship obviously with carriage of AMR bacteria and other micro-organisms.<br>…<br>There are plenty of studies to show that farm and slaughter house workers I might add are sometimes up to ten times more or have ten times the prevalence of AMR bacteria. Streptococcus Suis is a classic, but there is many others. So yes I would definitely encourage that, but that exists, it is verified in studies.
	Interview A:<br><br>I do note that companion animals as a source of AMU and transfer of resistance is not included. While this is relatively small, and I'm not sure if quantified at all for SE Asia, it may be another factor worth considering, given the trend for increased pets, AMU and close human contact.
	Interview A:<br><br>P: I think the other aspect is slaughterhouses. So it is not just on farm, it would be through the slaughtering processing of animals, slaughterhouse workers, and then through products being contaminated through that whole system literally into wet markets, where they are directly or through retailers like supermarkets. So we have also done direct study where we tested products in Southeast Asia and poor other markets globally from supermarket retailers and we found AMR bacteria including bacteria resistant to critically important antibiotics. So I think that needs to be accommodated, the slaughter processing aspect of that, of maybe production in particular.<br><br>R: Okay, and so that spreads from the slaughterhouse through all the way to retail sort of end of things, where they sell.<br>P: Yea.

	Interview A:<br><br>Transport and slaughter mentioned – but perhaps not wet markets for animals – a high risk area for stressed animals shedding high levels of faecal bacteria and AMR. I can't stress enough the major issue of informal and inhumane slaughter, even in 'formal' settings, in SEA – it is truly a hot bed of AMR risk for workers and food systems - aside from cruelty, which is documented for various zoonoses also. A lack of knowledge, training, standards and regulation persists….
	Day 1 workshop:<br><br>P: No. Okay, because and I think it is probably linked to what you mentioned with the macroeconomics that there is more trade.<br><br>P: Yes.<br><br>P4: In the country, so it is that it matters in your country and what you do, but it is this, if there is bad practice in one country and it is not picked up by import, export control, then otherwise, provide and I think that is what, I think it is not only with the livestock products, but we also see it with humans travelling, acquiring infections with resistant bacteria abroad and bringing them back to their own country to other people there, so I think the whole resistant problem is covered.<br><br>R: So is trade linked to your speaking just of animals, trade linked to your hygiene practices and all that, because someone knows your country doesn't follow, trade with you. Is that what you are ?<br><br>P4: Yea. Probably. I think it sort of captured in here, but at the international level it is probably a little bit…<br>…<br><br>P: Again those can international trade, for somebody else is suffering same with water shed somebody else is abusing, that is proven in some other countries, countries that share water sheds lately is responsible for antibacterials but in other countries – a very good example [name of SEA country] shut down for a few days…
	Interview A:<br><br>I guess the one thing is you have got the international trade, but domestic trade and transport perhaps is another node that may need to be in there. There are two aspects that come to mind on that. First of all there is a lot of long distance trade of cattle in particular in Southeast Asia, particularly between China, [and name of SEA countries] …And you know there is still live pigs going across from [name of country] to [a city in name of other SEA country] every day to the sorter, and there have been cattle going from [name of a SEA country] all the way across [name of another SEA country] into [another SEA country] etc. That may have

	changed a little bit and that is market responsive, and to some degree disease, but you have to consider all those borders are extremely porous. They might have tightened up certainly with ASF, but they are very porous, also connecting with China…<br><br>So and often these animals are transported on long distances under poor conditions. Sometimes carrying viral diseases like foot and mouth disease, which I have seen. Definitely they will try and treat for using both antibiotics and you know even lime juice or a combination thereof. So and then of course they might get other infections due to the stress of transport. So the domestic or regional trade of animals, particularly on that sort of peninsula is quite significant, and would predispose to not only the spread of disease, but needing for treatment and outbreaks of endemic sort of diseases as well as infectious or exotic diseases.<br><br>R: …Is that connected at all to the human side, to or other parts of the system?<br><br>P: I mean international trade agreements, I mean really I see is a block and you have to consider virtually the trade of its open block. They haven't got standards that they implement really for that trade. International trade beyond near out of Southeast Asia is another thing again. Domestic standards and targets, you know some countries have them, some don't, some of course a bit, some not at all, and so there is a difference between obviously the green and standards targets and the reality, and then does it link to any other parts. Well we know the, I am just trying to think, looking at your systems. It may link to predispose individuals if related to the agriculture. So that probably comes back to that node or connection generally with livestock illness directly to humans that are involved, either handling on farm or processing them, and usually traders and transporters are independent to farms and even processing. So they are another sort of well if it is a human thing and then it does I guess you could say it relates to spreading disease, potentially in the environment and obviously has an economic aspect to it. So yes I think it feels a little bit more again around that bottom right hand corner around trade and animal movement.
	Interview A:<br><br>P: I think the environment one, I am just having a good look at that. I think you have got, you have got resistance to food products, including obviously vegetable crops. You have got, I think you have got human transport within the regions. So human transport. There is obviously then you know ASF found in many products coming from this region in [name of Asian country] at various airport. So I mean that sort of transporting or transmitting exotic disease…
	Day 1 workshop:<br>…

	P: But the… the complexities of the health system is now changing the dynamics…So you have insurance coming in on one side. So the insurance it is pushing. Right. Then on the other side you have the affordability angle. Okay. On the other side, you have lack of resources of beds or doctors or nurses. All affects this actually. Not enough, not enough ICUs. Right. If you don't have a bed and all you have are very busy, crowded OPD, outpatient department. You have infectious disease people. So, infected people, infecting each other, and then of course [they can acquire hospital] associated infections…<br><br>P: Hospital acquired infections, the role of health facilities in acquisition of infection and antimicrobial resistance.<br>…<br>P: Yes, so you could argue that it is under that, but as long as you have got it specifically written down somewhere underneath. The problem, the problem is that antibiotics are much more likely to be used in hospitals. This applies to the first one, even more than it does in LMIC (low and middle income countries). Hospitals are the ones using the most antibiotics if it is a well-regulated environment. Therefore, inevitably there is just organisms in the environment in that hospital are much more likely to be antibiotic resistant, and they are not necessarily the same organisms that people are being treated for so they acquire infections with organisms which are already antibiotic resistant. It is just that the environment has changed by the amount of antibiotic use that is used and people can acquire infections in hospitals, even though they haven't got an infection themselves, and it is more likely they are antibiotic resistant that would be in that they acquired outside.
	Interview B:<br><br>There is also travelling of tourists for medical care. The quality of care provided in terms of whether the drugs are being prescribed appropriately will influence the AMR problem.

THEME: Agricultural Food Production Systems

**Agricultural food production systems:**<br><br>Different types of food production systems (e.g., vertically integrated, multi-purpose landscape approach) and how they operate.	Interview A:<br><br>P: I think, I guess so. I think that is sort of small… and unregulated, pretty much unregulated trade, and I mentioned, I attached a little bit on either "regulated trade" or trade within vertically integrated systems. So as you would have probably gleaned, the country is very hugely in the role and predominance of vertically integrated companies. So [name of SEA country] probably trumps that and then [names of three other SEA countries], but they would be, I guess though, vertically integrated companies have more control to some degree over antibiotic use generally, but they have contract farmers and usually they specify and control the drugs they have, but we have also found that you know there are things that go beyond those bounds as well. So there is an aspect of industry compliance and long compliance that perhaps is another node, and sort of underneath if you like, the level of national, international agreements and standards, etc. etc.<br><br>R: Okay, and by integrated, trade vertically integrated systems, it sounds like it is linking, if you can just explain that for me.<br><br>P: Yea sure. So I guess for example, in [name of a SEA country], 89% of [type of animal] and [type of animal] production is by vertically integrated companies, dominated by six or seven companies…and they will have their own farms. They will have contract farms. They will have their own sort of houses, and processing factories, and then there will be others that go to other slaughterhouses and processing factories usually some of the contract farms, and they may have their own retail outlets, or they may certainly supply B2B business or to wet markets, or to large supermarkets, etc., or they have as I say, their own retail outlets.<br><br>Now they will aim to control and they have [an agreement] with the government to restrict the use of certain antibiotics and they have an interest in doing that, and some of them have even gone to certification, of raised without antibiotics through the NSF or NFA certification in America. So they will have an interest in trying to reduce antibiotic and antimicrobial use and that is following an expectation to their contract farms as well. However, there will still be endemic disease on those farms. There will be endemic PRRS, porcine respiratory and reproduction syndrome, which works in a triangle of bacterial and management factors, to have clinical disease or not.<br><br>So there will still be inherent use of antibiotics and antimicrobials and they have more control on their own farms obviously than the contract farms. In relation then to trade and movement of animals, there is a large predominance as I say goes towards slaughterhouses and goes to their own pathways, but there is for some companies, like [name of a

	company], and [name of a company], which is another company. They will send and sell [type of animal], live [type of animal] for example, or breeder animals than breeder [type of animal] to other farmers within the country and traded to neighbouring countries. So that again comes to this domestic regulated and unregulated trade of live animals as well, as products.
	Interview A:<br><br>P: …but salmonella is another one. So for example, [name of a SEA country] has a high, stipulates a higher climate salmonella free eggs and chicken, etc., and that drives you know, produces to fill that market, but it might mean that they focus on salmonella, but and they trade for that, driven by trade and markets, but what industries tend to do and a classic example is in [name of a SEA country], is they are trying then to go to very hygienic barren, even slatted [inaudible] systems. So [name of the SEA country] has I believe predominately slatted [inaudible] systems for their broiler production on the basis of arranging things in theory hygiene, but also probably a lack of access to resources for deep [inaudible] bedding in [name of a SEA country], and so I guess that can lead to different types of infections arising but they might control more with coccidia stats perhaps versus antibiotics. The other one that comes to mind is that in the poultry production system and again this can affect workers directly, thinning is definitely still done. It is still done in [name of region of the world]. So thinning is where you probably know where you might go in at thirty days, and harvest a proportion of the birds and let the rest go out, say to forty-two days, and that process of course is a classic breach of our security, and risk factor for introducing campylobacter. So of course whether that can be you know linked potentially directly to humans or the products or introduce it to the remainder of the flock, and campylobacter is probably the number one food borne disease. So practices like that relate to the systems.
	Day 1 workshop:<br><br>P: But I think again here, the thing to do is the [inaudible]. From a systems thinking perspective, [inaudible] treat sectors different than other programs end up with problems. Take it, for example, [name of a SEA country], [inaudible] take a landscape approach, [inaudible] technical [inaudible] performing area, you have rice, you have fish, you have livestock, you have good meat, you also have a connected food system. So there everything is happening. Excess use of pesticides and chemicals in the fields going into the water, human waste mixed gets into the water, water is to fish, so there is the constant movement, so it is not only the AMR going from fish to humans, it could be AMR going from humans to fish. [Inaudible] we could have an endemic disease, lots of treatment and all of humans are affected and low purification, seaweed contamination and poor farming and fish. Fish could be getting left from the humans. [Another P spoke but inaudible.] So I mean… Water is important, water is

	the median. Whoever is using the water can be contributed to the list. [Another P: yea] Can be exposed to the list.
	Day 1 workshop:<br><br>P: This is where I think you need to consider [inaudible] industrial farming… that I can [inaudible] in …other [non SEA] places…how a single landscape is used. [inaudible] Landscape is exclusive for tree, or exclusive for [inaudible]…where [in name of three different SEA countries], the landscape is used for multiple purposes.<br><br>P: This farming is quite low cost.
**Agricultural food production systems:**<br><br>Government service officers	Day 1 workshop:<br><br>P: Technical services from private sector technical representative do not reach small farmers, while services from the government are also limited.
	Day 1 workshop:<br><br>P: Regarding antibiotics should be using for the livestock. I will give you an example. Since last year [name of a SEA country] ban for ATP, but I just attended [inaudible] the private sector company last month, even though we ban ATP in [name of the SEA country]… but the antibiotics still high, they still put it in livestock. Because the farmer worry, because they worry… how to protect the farm.<br>…<br>R: So in what way… is it, is it it's expensive? It is a change in management practices? It's a change in behavior? Is that what you are…<br><br>P: Yes.<br><br>R: It requires changes in management systems and their behaviours.<br><br>P: Yea, and also try to involve the government officer.<br>…<br>R: … Can you explain that a little bit more? They involve the government officers?<br><br>P: Sometimes farmers afraid with the officer. [R: ah] Ask if the officer… say to the farmers that should be in different farm, they sometimes will … will following the suggestion.<br><br>R: So if there is a government officer, telling them that they need to change.<br><br>P: Yes.<br><br>R: They will follow it.<br><br>P: Yea.

	R: But that doesn't always happen?<br><br>P: Yes of course.<br><br>R: Meaning the government officer is not always present. So are these government officers, I don't understand the context. So they go onto the farm?<br><br>P: Yea. For veterinary service.
**Agricultural food production systems:**<br><br>Animal welfare	Interview A:<br><br>P: The relationship link between animal welfare towards growth promotion AMU is missing – this is key, higher AMU is associated with low animal welfare, used to prop up conventional systems.
	Interview A:<br><br>P: And I would say there are a couple of perspectives where I would say have relevance to the nodes for a start. Obviously, one is clearly animal welfare, but also livestock disease and systems, agriculture, livestock systems…
	Interview A:<br><br>P: Ideally we want and know 'high' welfare is best associated with reduced AMU but 'higher' might be more realistic in South East Asia.
	Interview A:<br><br>P: I guess focusing on animal welfare, one of the things that became very apparent to me and it is not uncommon, because it is still not well considered in this core system is the common discourse…about livestock illness affecting animal welfare, and farm hygiene practices affecting animal welfare, but I guess we bring the perspective… that low animal welfare contributes to low immunity, contributes to livestock illness and contributes to aspects of farm management, and a key example, so I see the directions of these relationships need to be both ways, and then the polarity considered.
	Interview A:<br><br>P: So while it is like illness, yes of course with animals that are ill, it impacts their welfare, but if their welfare is low in the beginning, it predisposes them and lowers their immunity and increases their susceptibility to livestock illness and obviously conventional production systems need a direct link back to welfare as well with a double arrow.
	Interview A:<br><br>P: …painful procedures, in the map already… are routinely associated with AMU plus additionally with complications. As is early weaning.

	Interview A:<br><br>P: So you have got here for example treatment of post procedural, post castration dehorning, tail docking, teeth clipping. The reality is, it is not usually, it is partly for treatment, yes, but it is also antimicrobials that are given routinely at the time of these procedures, and it is a blanket use usually in most industries it is equally those that are not well regulated for antimicrobials. So that is a key area, where we see and there have been clear reductions in antibiotic use when farming systems move away and avoid painful procedures for example.<br><br>The other very concrete example is waning times.<br><br>So in systems of course the high efficiency and economic and probability reasons, they will reduce the waning time, and yet there has been good work shown in Europe where waning, for example in pigs, when waning is at a minimum of twenty-eight days, ideally even later, then you can significantly reduce post waning illness, and use of medications. There are other examples related to conventional production systems in relation to overstocking, barren environments, poor mixing practices, which lead to biting and behavioural stress, etc., and then again with immunity endemic diseases and so what we are very much talking about now are the underlining aspects of a low welfare conventional system that predisposes to sub-clinical or clinical disease, and metaphylaxis or prophylaxis or treatment of animals and that is a huge aspect that is directly linked to animal welfare.<br><br>P: So I don't see any connection between animal welfare and conventional production systems given I guess it is important, yea and I don't see anything between animal welfare and producer profitability that may or may not affect directly…
	Interview A:<br><br>P: ... So and that is a key one and it means something around waning times. You have got conventional systems and animal density, but there is nothing around waning and waning or brooding for poultry, or similar for the aquaculture is obviously a high, it is a time of peak use of antimicrobials. So I think that is an important aspect…
	Interview A:<br><br>P: So tail docking is another classic example, where a low welfare system can lead to significant use, outbreaks of tail biting that is related to many underlying factors, not just welfare, but other aspects of the conventional system. So thermal comfort, aspects of concentrated diets and feeding, competition, and all aspects of stress, etc. etc. So that is really the key for us and that also definitely relates to aquaculture, of course as well, and with a number of factors there…

	Interview A:<br><br>P: So one thing that perhaps is not on there is conventional production systems, I don't know if you, genetics, so fast growth genetics, hypo prolific sow genetics where they have got excessive numbers of piglets to the sow teat. All those things add stress on the system and stress on animals and therefore predisposed to illness and use of antimicrobials. So there is an element that makes me think of in terms of genetics that is perhaps not there.<br><br>R: Right. So its own node possibly or something. Yea.<br><br>P: Yea, it is sort of part of, initial production systems but the genetic companies and the demand of course from the industries really drives that and it is, it is happening faster in some Southeast Asian countries than others. It is sort of reached near max in [name of a SEA country]….but in [name of another SEA country] broiler genetics, there is still a gap in growth rate potential, and that may correlate with endemic illness and lead to antimicrobials. So it definitely can impact…
	Interview A:<br><br>P: Where animal density is mentioned – it should include also ….confinement, fast growth genetics – this relates to the above concerns also, but there is clear scientific evidence that increased confinement leads to increased AMU eg. tethered dairy, crated sows – mastitis, metritis, vaginitis, urinary infections. While also recent evidence to show the converse is associated with reduced AMU.
	Interview A:<br><br>P: Also – the risk of ineffective antibiotics for treatment and the potential for animal welfare plus morbidity and mortality risking food security in future.
**Agricultural food production systems:**<br><br>Cost of pesticides versus antimicrobials and how these are used in food production.	Day 2 workshop:<br><br>P: Yea, of course. If you find good letters in your home market that means you know that it has been a rejected sample. Yea. Don't worry you can go and eat in [name of SEA city]. You should worry about, you should worry about salads, you know, because …<br><br>R: Wait why?<br><br>P: No, no because they said, there was a quick test by these … scientists from University of [name], even around randomly picking up samples you know from markets.<br><br>P: Supermarkets or wet markets.<br><br>P: Supermarkets are fine, but the wet markets.

	P: Okay.<br><br>P: And many of these stalls, many of these shops, they buy from wet markets. Yea.<br><br>P: Yea.<br><br>P: And farmer markets, and a lot of them are contaminated with e-coli and that kind of stuff, because people use all kinds of organic manure. The thing about one thing maybe you need to capture is the cost of using antibiotics is probably more expensive than the conventional pesticide. So farmers will not resort to using antibiotics if they have a choice. They don't have a choice. So it is a cost factor. So it negates unless your investment is high. Like citrus is expensive. So therefore you can afford to use antibiotics, but if you are using, if it is no cost, costly, and it offsets your market value, then …<br>P: Like the local spinach.<br><br>P: Yea, they wouldn't want to use it on a very cheap crop. They might as well use any pesticide or grow another crop you know. So it is a cost factor, and costs of pesticides is so low now, it is about 5% or less of the total cost of production, because China is now dumping so much pesticides. It is called the pesticide tsunami, basically. Oh yea. Every country in Southeast Asia is facing this problem, because [name of a country] produces a dirt cheap price and so farmers can spray any amount of pesticide, and antibiotics are only coming as a last resort when they can't do anything, and that is costly, yea, because you can't simply go to a market and say, I want a bottle of antibiotic, you know no way. No way. It has got to be very, very precise. It is going to be very, very expensive. Yea.<br><br>P: So if we change the cost differential between the antibiotics and the other therapy that could change antibiotic use practices. So it would make them more expensive.<br><br>P: Exactly. So this is a regulatory thing. You have got to put our differential and it has got to be for a very premium sort of… premium sort of problems I would say.<br><br>P: Situations that exist.<br><br>P: So human [health] is a premium you know.<br><br>P: Yea.<br>…<br>P: Oh I had a big problem. I studied in, I was in [name of country], yea for a number of years. I studied there, and the first thing the doctor did was

	prescribe antibiotics. I said what the heck you are doing you know. This is wrong. Yea, but oh yea as you say, patients, yes sometimes. So the prize factor, differential I say negates the use. So if you put a premium on availability, in terms of cost, price, then I think it sort of negates the… how would I say the… impact of you know use in terms of extended use whatever.<br><br>R: So there are two different costings happening there. If you have a really profitable crop, then you can afford to use antibiotics.<br><br>P: Exactly.<br><br>R: But then also the amount, the price of, was it the alternatives, change …<br><br>R: Yea, other options, because I think it is not even another product. We have a situation in [name of non SEA country] where it is cheaper to give an antibiotic than to increase ventilation in the farm, so if it is just a cost thing.<br><br>P: Exactly. Exactly.
**Agricultural food production systems:**<br><br>Use of antimicrobials to reduce food production costs and meet consumer/market demands.	Day 2 workshop:<br><br>P: Okay, let me continue with the nutrition and [inaudible]. Okay. So I talk about food security first okay, because when we talk food security then we will talk about the affordability as well, okay. So what will affect the affordability is that the demand and supply. Of course, if you want all our citizens to be able to afford to buy…okay the food okay, definitely we need to increase supply. Okay, so that we can reduce cost. So how to increase supply. So definitely we want to ensure we have a good production system. Okay. We want to reduce [inaudible], we need to reduce this. So what normally the farmer or the grower, okay, in this country they use antimicrobials. So because based on my experience that when we are collecting the samples, okay, from a farm level, so we always heard about where from the farmers they say they tend to use a lot of, drugs or antimicrobials instead of just for preventive purpose. So even they use prohibited drug, okay, even they use a prohibited drug…for a number of agriculture…<br><br>R: Okay.<br><br>P: Yea, because we don't have a good regulatory framework to control.
	Day 1 workshop:<br><br>P: And the third one is the producer, like a farm itself…because talking about the nature, this is quite natural for the people who are doing the farm animals. Everybody wants their animals to be healthy and live in a good way, because that product is live animals. Right…So that is their own

	nature. So if you talk to the farmers, usually the farmer didn't want to use an antibiotic anyway, because it is cost for them.<br>R: Okay.<br><br>P: No but because the farm, they might lack of good security system.<br><br>R: They might lack it.<br><br>P: Lack, because that is why the animal gets some sickness or illness.<br><br>R: Yea and they don't have the money to spend on antibiotics.<br><br>P: Yes. They don't want, usually they try not to spend any kinds of costs in that production anyway, because they want to keep low costs as possible. Right.<br>…<br>P: But because of lack of the good biosecurity, that is why it is forcing them a little bit to either use the antibiotics or alternative for treatment. Right, but the most difficult for them is biosecurity is not only management, it is an investment. So many of them, they don't have financial enough to invest, investment in good biosecurity on the farms…So that is why I think these three multiple areas are kind of linked together. The consumer know, the public want the producer to do good job, but they need good financial to implement the good farm biosecurity, but when they implemented they are selling to the market, the consumer didn't aware and not respond to the product, so they want cheap, cheap, cheap, but don't want to pay, so at the end it kind of obstruct, the circle doesn't move, it kind of obstruct everywhere, not go nowhere.
	Day 2 workshop:<br><br>P: We have all this argument about producing wholesome food. I agree with it, but we always, I am very much going to organic agriculture. I work with organic agriculture, and I always emphasize the fact that we don't mainstream organic agriculture. It is for niche markets. It is only for certain people who want it, because you have got to pay a premium for it, and you can't really solve the food security issue in a country, you know. You can't mainstream organic agriculture, because your production is not enough to really meet you know the global demand yea. So but the idea is when you mainstream agriculture, then you have to use certain conventional approaches, because it is indispensable otherwise, because the way we have actually sort of structured our agro eco systems. You look at chickens. You know previously chickens were all roaming around during our time. I would say, our time, meaning '60s, '70s, but then people wanted more chicken so the battery system came.<br><br>So when you talk about self-sufficiency in [name of country], we are 120% self-sufficient in chickens, which means you know people, the price can come down to dirt cheap. Yea, but in a battery system, it is really highly

	industrial system, which people want, so that they want quick turnover. They want fast. So therefore, you need to keep, because of the high-pressure system. You need to keep them really healthy, and this is where the antibiotics come in, and so. So it is very difficult to stop that business part of it running…
	Day 1 workshop:<br><br>P: First is the customers. So we have customers. We have customers from the experience when we actually both agree when we do the business right. So usually it is very hard for the kind of chicken and eggs, no. People want to push the producer to do the right things, but the producer usually says if you do the right thing, we cannot compete into the market, because the consumer doesn't have economies to buy the products that have to invest. So kind of pro<br><br>R: That is right.<br><br>P: But the thing that we do agree if the customer, the customer mean between consumer and the producer said this is what we want and that is the market. Most of the producers you say okay we can do it.<br><br>R: Okay. So it is consumer demand.<br><br>P: Yes.<br><br>R: Is influenced Can be influenced<br><br>P: Influenced. also most of the time become like demand, demanding.<br><br>R: Demands. Products, the producers will comply.<br><br>P: Yes. I think actually, when we did cut this level is very quick.<br><br>R: So easy to do or fast to do?<br><br>P: Fast to do, because the producer can shift in the next day. Very quick.
	Day 2 workshop:<br><br>P: …Okay. I shared with [P] this morning, because our consumer always demand for beautiful produce. Okay, beautiful produce, and bigger produce with a cheaper price.<br><br>It is impossible, it's a demand from the consumer. Okay, so we have to, okay, like how, okay we have to take all the costs away because of our demand.<br><br>R: So how do we map this? So I heard economics. I just heard population demand.

	P: Okay.<br><br>R: Right?<br><br>P: Yes.<br><br>R: Is that what you are saying?<br><br>P: Yes.<br><br>R: Will drive supply and demand?<br><br>P: Yes.<br><br>R: And people want to produce the higher quantities to lower the cost?<br><br>P: Yes.<br><br>R: And that leads to increased use?<br><br>P: Yes.<br><br>P: What he said it should look good, should look good.<br><br>P: Yes, look good as well.<br><br>R: Go directly to preventative use.<br><br>R: Okay.<br><br>P: Yea.<br><br>R: In order to lower cost, they use preventative…
	Day 2 workshop:<br><br>P: Right. Right, but I think the whole thing is driven by market. Like for example like what you were was saying about if the consumers put pressure on them, it's very simple. People don't want to look at alternatives, because they are, well we are not investing enough money in alternatives, simply because we have the convenient things to do. It is something cheap. Something, you know, but the moment the market demands, market meaning consumer, right up to suppliers, then you realize that people will try and find alternatives. So it all goes back to the regulatory process, the …

	Day 2 workshop:<br><br>P: I think the key thing to sort of point, is what are the factors that drive antimicrobial resistance. There are major driving forces, you know. Pull and push factors, yeah? markets, security, food, affluence, you know, changes in lifestyles…
	Interview A:<br><br>P: you know there is price capping, there is government price capping in this region on commodities and products, livestock commodities and products and so that again adds a pressure to the system to be able to produce fast enough, the profitability. So actually I don't see that there in terms of you know government subsidies, government price regulation, that sort of thing. It does have, definitely has a relationship to profitability in conventional systems.

THEME: CONSUMER DEMAND

**Consumer demand:**<br><br>Cultural, religious and lifestyle factors influencing consumer demand	Day 2 workshop:<br>…<br>P: … Actually this is our fault as well. Okay. I shared with [P] this morning, because our consumer always demand for beautiful produce. Okay, beautiful produce, and bigger produce with a cheaper price.<br><br>P: It is impossible, it's a demand from the consumer. Okay, so we have to, okay, like how, okay we have to take all the costs away because of our demand.<br><br>R: So how do we map this? So I heard economics. I just heard population demand.<br><br>P: Yes.<br><br>R: Is that what you are saying?<br><br>P: Yes.<br><br>R: Will drive supply and demand?<br><br>P: Yes.<br><br>R: And people want to produce the higher quantities to lower the cost?<br><br>P: Yes.<br><br>R: And that leads to increased use?<br><br>P: Yes.<br><br>P: What he said it should look good, should look good.<br><br>P: Yes, look good as well.<br><br>R: Go directly to preventative use.<br><br>R: Okay.<br><br>P: Yea.<br><br>R: In order to lower cost, they use preventative<br><br>P: Yes, especially during the festive seasons, okay, during our festive season, like Chinese New Year, like Hari Raya okay, because have a lot, we have a high demand. So in order to fulfill the demand of consumers, so

	they tend to use a growth hormone. Okay, they tend to use the antibiotic growth hormones. So this is so, okay, actually under the population demand for products. Okay. So in order to fulfill the demand, okay,
	Day 2 workshop:<br><br>P: Certainly these two approaches are like complementary, so what drives the intensive use of pesticides but also the [anti]microbials is the demand for lower food, for certain types of food, like more meat. So if we act on the drivers, our food consumption patterns, and so on, we can also reduce the amount of meat that needs to be produced, and therefore the amount of antibiotics that are used to produce this meat, and of course meat will be produced anyway. It is a matter of producing it with the minimum or the most cost effective or effective better use of antimicrobials. So this prevention is used.
	Day 1 workshop:<br>…<br>R: But people, so if we try to link it to maybe like consumer demand, the people go there for different reasons than they would go to retail.<br><br>P: Because of the people [from country] still believe that even you buy, we call chill and and warm meat. When they go to the market, it is kinds of warm meat. Right<br>…<br>P: Warm, warm meat, but if you go to the retail, all will be chill or frozen.<br><br>R: Oh I got you…<br><br>P: So the culture of the probably Asian they don't buy the concept of the frozen meat yet.<br><br>R: Okay. Yea. The population demand for a particular product.<br><br>P: And sometimes they prefer for example like chickens, sometimes they prefer to buy live chickens than just the meat chicken. That is the culture part.<br><br>R: Okay…<br><br>P: And you may well have covered it but it is food preparation practices. Talking about food production, it is more than just that. It is whether people for cultural reasons eat cooked food, or whether they eat raw food. It is a large culture particularly in [name of country] and probably in parts of [name of country] as well of eating raw food. Raw meat. Blood. Raw fish, which increases transmission dynamics for chances.<br><br>P: A lot of street food.<br><br>P: Yes.

	P: That maybe affect to the human health.<br><br>P5: Yes that is right.<br><br>P: That leads to the hospital.<br><br>P: Yes. It all relates to the excess right.<br><br>R: So did you get them moving to human health.<br><br>R: So the cultural factor is going to help, culture preference is the food and how you prepare food and that typically affects hygiene.<br><br>P: Yes.<br><br>R: And then from there goes to food safety<br><br>P5: It will go to food safety. It will increase burden to illness potentially and if there is antibody resistance in pathogens that are in that food that will contribute.<br>…
	Interview A:<br><br>P: Yea, because some of them [alternative proteins] are actually even produced here, and then there are two types. There are these new trendy ones beyond meat and those kind of things, which are definitely infiltrating into the region, and there are platforms that are actively doing that, and there actually, there are existing low cost often Buddhist orientated ones, and so you know and there is a whole lot of you know social culture and dynamics around all of that, and meat eating generally, which you know we are exploring, but you know it does interrelate into essentially into the food system, which might interrelate into, certainly would relate to some degree, conventional production systems. So I guess, yes you know as knowledge about AMR as well increases and the linkages of all these things, in awareness and dietary preferences may shift or diversify, or be more flexible, etc.
**Consumer demand:**<br><br>Demand for inexpensive, aesthetically pleasing, and convenient food.	Day 2 workshop:<br><br>P: I am looking at my perspective as educating food waste in this country, like what I know is we actually are practicing a very rotten food system locally, you know. And I think starting all are quite man made from people like us. Like what the (name of participant) was mentioning. We demand perfect food, and want it cheap and so on. And on the lifestyle, the fast food, the convenience store frozen food and all these things we have created convenience is all come from this people, and the population is growing. I mean whatever you are trying to do is fine, but the way I look at it, it is just one small portion of what is here on the production of food,

	right? I look at it from my perspective as a layman when I sit down here, is another shocking thing for me, that I can put into my [inaudible] whatever I meet in the future, you know. What I know is it is all created from evolution and so on, the lifestyle, the population, and that creates, things become very commercial. We need to produce at the speed and everything commercialized, packaging material and so forth, and we abandoned organic farming you know. In that sense, that to speed up, because our rejection of imperfect food we need to, farmer has got to plant more to sort of offset the rejection. So from the farmer, there is a lot of losses and so forth. It is all in the name of profitability. This is what is happening, and with this, when it comes to production, to do it we need to administer like more antibiotics, herbicide, pesticide, whatever, so that you can get a better yield, you know, and this component is basically here what we are looking at.
	Day 2 workshop:<br><br>P: Our consumer behaviour lifestyle change and we are willing to pay this convenience frozen food and so on. So the producer, put more things out there. I mean look at our dietary requirement. We, most of us has surpassed our dietary requirement, you know. We don't eat to live anymore. We live to eat. You know, we are twenty-four hour convenience store. In [name of country] we are twenty-four hour MacDonald, KFC…Yes. So we are changing. When you do that, open more time to eat more likely to eat, you cause food waste…….
	Day 2 workshop:<br><br>P: … and everybody wants cheap food and therefore they don't want to pay for a lot of you know from the cost of labour to setting up systems which you know sort of underscores cleanliness and so on. So if you really look at you know developing countries, developing economies, basically we want cheap food, so that Malaysia is very cheap relatively compared to a lot of other countries, and so people can get eat twenty-four hours a day everything is so convenient…
	Day 2 workshop:<br><br>P: … I want to go for a very cheap meal, and so the price keeps going up, but the sad thing is where people really care for good food production, like maybe some of these organic farmers, then you really internalize the cost.
	Interview A:<br><br>P: Similarly, in a positive way – on the consumer choice etc node – increased plant-based products could be added - traditional / religious use [is] quite high in SEA plus recent newly introduced formats involve far less to no antibiotic use and are trending positively and projected to significantly increase.

**Consumer demand:**<br><br>Demand for alternatives to meat and safer food supply.	Interview A:<br><br>P: …but I guess alternative proteins and now are starting to increase slowly, but in this region and might shift things a little bit. I suspect they won't take the pressure greatly off livestock systems in the short term, but they may over the longer term, but I think they will just probably you know add to the bundle of things that provide food security…
	Interview A:<br><br>P: I mean food safety is a key concern in this region, and that so we know actually from some of our surveys, I must reiterate it again with a colleague from [name of a SEA country], certainly [population group within name of country], at least I know for a fact, but they are only concerned about antibiotics, chemicals, hormones in their food. So that consumer public awareness, food safety aspect is very, is one of the top factors that feeds into the livestock production system and for people in this region definitely. Yea.
	Day 1 workshop:<br><br>P: That certificate is different from the OIE international trade certificate, so there's always the impression that people go to the supermarkets, see how they are certified. Who is certified? An independent [inaudible] system is certified. Countries want to be certified. It is a niche market certification. [inaudible] certification, land and soil certification. So those certifications are distinguished from the clear [international] standards.<br><br>R: And would this certification also, would consumers want …<br><br>P: It is consumer driven also, it is a consumer driven market driven you know. Third party, [R: we don't have it], you don't have it, but it is a big contributor. They are all certified for example. Consumers only buy whatever organic poultry or whatever, then it is happening.
	Day 1 workshop:<br><br>P: There are a lot of production process certification at each level. It is responding to the market. GAS certification is a big thing for aquaculture, global aquaculture [and land] certification. They certified hundreds of farms in [name of country], [name of a different country], [name of another country]. So the market is happy to accept that. So then one of the component will be responsible use and less use or no use of chemicals in farm practices.<br><br>R: so it's global and domestic product certification?<br><br>P: yea, certification, yea, just two.

**Consumer demand:**<br><br>Demand for antimicrobials	Day 1 workshop:<br><br>P: Not only for [inaudible] but also [inaudible], that the [inaudible]sometimes we have a perception if I am, for example, I want to seek for general sickness .by going to a specialist and asked the doctor to give the high-level antibiotic rather than low level antibiotics. [inaudible] Yea, this is people's perceptions with the doctor is specialist that he can give me, they have [inaudible] better than [inaudible]<br>…<br>P: doctor, the people and the doctor who gives more drugs.<br>Yea, so they are more likely to prescribe.<br><br>R: Is that sort patient demand for doctor driven by the patients?<br><br>P: Yes. There is patient demand and patient expectation for the doctor. Competition also. Doctor incentive.
	Day 1 workshop:<br><br>P: It might be, it might be access to the antibiotics rather than prescribing behavior. Because some antibiotics aren't prescribed at all. They can just go into the pharmacy and get whatever you want.<br><br>R: And grab, over the counter.<br><br>P: yea if they can afford.<br><br>P: yea exactly.<br>…<br>P: Some people go straight to the pharmacy shop. They don't want to go to the doctor. They don't have time to wait at OPD or they don't have the money.<br><br>P: Yea<br><br>R: Okay.<br><br>P: Or they just, basically want something to relieve themselves ….
	Day 2 workshop:<br><br>P: But sometimes this one I think it is demand by the patients, because even [if] the doctor do not prescribe the antibiotic to the patient, the patient will not really trust the doctor. Yea.

THEME: Access to antimicrobials, diagnostics and alternatives

Access to antimicrobials	Day 1 workshop:<br><br>P: … health care professionals and health care facilities, because the health systems and its issues also affect this. It is not just the health care professionals…So you may have [inaudible] systems, which are [inaudible] private coming up. They are charging more for example and therefore affordability issues, lack of diagnosis, the health systems itself is a major, the way it is changing in many countries is a major issue.<br><br>R: So question then. So the type of health system available, we have more private health care systems. What does that do to use?<br><br>P: So [an individual] did a study once where for example all the primary health care facilities run by the government stocked only cotrimoxazole . Right? So that is one side of it. Okay? On the other side, you have all the private hospitals and clinics selling only the third generation cephalosporin because that is where the profit is, and currently might be meropenem and might be even posted. Okay?<br><br>R: So it could be either.<br><br>P: Yea, we're saying, it is not a hospital. I am talking about a clinic and they give an IV meropenem to outpatient. I am not kidding.<br>…<br><br>P: But the… the complexities of the health system is now changing the dynamics…So you have insurance coming in on one side. So the insurance it is pushing. Right. Then on the other side you have the affordability angle. Okay. On the other side, you have lack of resources of beds or doctors or nurses. All affects this actually.
	Day 1 workshop:<br><br>P: So, my first thought was health care facilities, hospitals, and clinics. Where the people access those places. Those are important. I don't know how you measure it.<br>…<br>R: So if their increased health care seeking behaviours, there might be …<br><br>P: Increased access to health care facilities?<br><br>R: And if you have increased access to health care facilities, you might have decreased, decreased [inaudible]?<br><br>P: Well you might have. You might have…as it goes in lots of different directions. You might have increased better management of infections, so

	…increased access…Appropriate use, hopefully, not necessarily. You might actually increase the inappropriate use.
	Day 1 workshop:<br><br>P: The only thing here is that we are on the one hand governments hospitals often have an access priority, has an access issue. So the only thing is how we fit this into both.<br>…<br>P: It is certainly a challenge. The access issue you talk about, the lack of access to antibiotics and especially in very rural areas, compared to in big cities where there is all private medicine and drivers towards using more expensive antibiotics, more broad-spectrum antibiotics.<br>…<br>P: Yea, and the actual provision of medical care.
	Day 2 workshop:<br><br>P: Now, no. Government doesn't officially have a list of antibiotics that can be used in agriculture.<br><br>R: So does that mean people aren't selling them anymore? Or is it like a black market thing?<br><br>P: It is a black market thing. It is not legal yea. It is not a registered list, because a registered list you can use, yea.<br>…<br>It doesn't matter even, pesticides go into you know black market you know. So a lot of unregistered pesticides are also being sold, but that is all cross border trade and that kind of thing.<br><br>P: But yea, but that is like a big barrier in [anti]microbial use.
	Day 1 workshop:<br><br>P: And then you are talking about they probably will go to quacks, right, and not to professional doctors. That is also seeking behavior. Probably they want to mix of medicines. So for example of a specialist, pharmacologist okay. I won't mention which country, but it is not [name of country], [others laughed] where they were making these medicines then. I was curious because there were around a thousand capsules. So I asked them what, you know, what is this for, and they said, one doctor actually prescribes this. I said, why are you making a thousand. No he gives it to all the, all the patients. [P laughed] I said, what is in it. One antibiotic, one [narcotic], ones to [inaudible], one anti-inflammatory, possibly one vitamin. You are covered.
	Day 1 workshop:<br><br>P: Exactly, and I think one important point that's missing, and I don't know how you wanna reflect that is the cross over use. So there is actually considerable use of human drugs in livestock.

	P: And they are accessed through the pharmacy. So they actually skew all your statistics about what's the use of human drugs, because they end up… in the chicken, in the chicken. Yea, I think in [LMICs]…<br>…
	Day 1 workshop:<br>…<br>P: …obviously then there is the whole questions about access versus excess and we discussed it yesterday already, because in a way, not representing very well the poorest in our society, when I heard something like that, because they actually need to have access to drugs, but then that is maybe another problem to be solved through other means, but I think the risk by having too much over the counter sales, almost outweigh that, so I think it is a bit of a cost benefit.
	Interview B:<br><br>Fake drugs that have no active ingredient is another issue. Patients take drugs that are ineffective and so what impact does that have if any?
	Day 1 workshop:<br><br>P: Buying of [inaudible], so doctors, health care, prescription, everything is fine. Again in many countries, in low income countries and more areas, buying medicine is also not [inaudible], people can buy it over the counter. So all the things or talk on fisheries and aquaculture and livestock, also [inaudible] many people from reusing the one-year-old, two-year-old prescriptions, but many are never reusing the prescriptions at all, they are still buying antimicrobials and antibiotics over the counter, so the regulations are so poor. Now with this is online now, it is available. For example I can tell you [inaudible] it's like [inaudible] online medicine has become so [inaudible], no prescription [inaudible] now …. because I can [inaudible] those scripts [inaudible] ten minutes it comes to me, you know [inaudible] I just pay online Goggle pay [inaudible].<br><br>R: We had a situation where you could buy online meropenem with an aminoglycosides for poultry feed.<br><br>P: Poultry. But here, human.<br><br>R: Yea, so …<br><br>[P said something inaudible]<br><br>R: Yea, and that you saying that applies to humans, to everything.<br><br>P: To agriculture to life, it continues. It is the same. I think it is like a central thing.<br><br>R: Yea.

	P: Because there is a big overlap… in the… [R: in the crops too?]
	Day 1 workshop:<br><br>P: Do you discuss the role of the quality of [antibiotics]?<br><br>R: ….So how would we… it's quality of…<br><br>P: It's quality access.<br><br>R: Do I have the direction right?<br><br>P: Quality access… like… The arrow the other way. Both. Maybe.<br><br>R: Think if the quality of the drugs has declined, how does that affect, so how do we tie that in so we make the quality of the drugs measurable.<br><br>P: You mentioned something about efficacy, future efficacy… [inaudible]<br><br>P: It ties into the future efficacy as well as the human use, and it also goes to the burden of illness.<br>…<br>P: Yea. Say drug quality.<br><br>R: Direct?<br><br>P: Yes.<br><br>P: And this is actually again common. The same issue can be [applied] to animal sector.<br><br>R: Do we tie this to regulations?<br><br>P: Yes. That is also tied in.<br><br>R: We need a tie into regulations because there is the provision of poor quality or counterfeit drugs. Not control is a better way to phrase it.<br><br>P: Yea, it's not… nothing is going against it, and it is actually then linked to the access that …describe impact this, that we combine.<br><br>P: Regulations and drug manufacturers. I think that is a very large subject.<br><br>P: Because it is at every level of the use. Right, because from the drug to final take.<br><br>R: So is it regulations of drug manufacturing and distribution maybe? Then that would capture for the distribution of the drug and how it gets to the end user.

	P: Yes. Exactly.<br><br>R: And that is tied to what again?<br><br>R: The quality of the drugs. So as you have more regulations of the drug manufacturing and distribution, we will have improved quality of drugs that people can access.<br><br>P: But also I mean it is at every level. So we are talking about the manufacturer. We are talking about the [inaudible] supply chain. We are talking about what can be [stocked?] in the pharmacy shop or health care clinic, because there is a classification now. We are talking about prescriber, dispenser, administrator. We are talking about the patient also. So, it's actually…
Access to diagnostics and alternatives	Day 1 workshop:<br><br>P:…So it is the same with any animal health service provider, and also they haven't got a clue what this is they are looking at…<br><br>P: I think it is both. It is a bit like…an inability of the service providers to do something else, because they don't have the diagnostic tools. They don't have the knowledge. They don't have the infrastructure.
	Day 1 workshop:<br><br>P: There is often a big circle of potentially increased resistant human infections and increased burden, etc. etc. So lots of links there potentially.<br><br>R: Health care costs.<br><br>P: Yes. Definitely. All of those. Okay so that is step one. The major one which I would like to talk about, which we talked about in the last couple of days is diagnostics, access to antibiotics, and making a diagnosis in the first place of what the infection is and testing that for antimicrobial susceptibility.
	Day 1 workshop:<br><br>P: like crazy saying that probably give me an answer on that, but what I worry about from a point of view is we somewhat know the adverse effects of antibiotics now, but we definitely do not know many of the new drugs are on the horizon, because too artificial… are built to bring out efficacy and not adverse effects in the triangle of epidemiology or whatever you want. So even probiotics I will just hold on and see what actually happens, though natural probiotics like we know from experience safe.<br><br>R: Some of the novel products are challenging the regulatory systems. There is something, like nano particles that we don't have, to evaluate human safety. So it is challenging.

	P: So the other small angles on probiotics is that especially in and also [name of country], there is a lot of fixed dose combinations coming.<br><br>R: Fixed dose<br><br>P: Yea, so for example we just an expert for that through the Supreme Court and talking about let's say or you know. Now the next round is one antibiotic and something totally separate. It could an, could be a, could be anything. So that is another huge issue in many of our countries actually, where very irrational combinations are present, which again back to the point of diagnosing, because I don't blame the physicians and vets also. Something they are not clear what the diagnosis is. So you try and give broad spectrum, but that hits microbiome<br><br>P: Yea.
	Day 1 workshop:<br><br>P: For the livestock side, so I think you got some of the drivers, that drive use, including probably non-access to non-AM alternatives,
	Day 1 workshop:<br><br>P: You have got development to those alternatives, microbes, but we do have alternatives already, some of which are vaccinations, things like TB. Not a very good vaccine, but it has some effects sometimes. vaccine very good. Typhoid antimicrobials. Typhoid has been a huge issue for many years particularly in this part of the world. Vaccination as an alternative strategy for controlling the number of infections that there are in the first place. It is a very strategy.<br>R: So we have it [on the model] in the animal sector, but we don't have it in the<br><br>P: Exactly.<br><br>R: So just like use of this.<br><br>P: . Exactly the same thing, just needs to apply to human medicine too.<br>…<br><br>P: Even for the interaction now. This is like the …like the human behaviours. Policy.<br><br>P: And the same applies to disinfectants and antiseptics as well.<br><br>R: And I think you had a good point that you touched on, it is not necessarily development of alternatives. It is access and availability of alternates, because there are a lot now that exist?

	P: Yes.
	Day 2 workshop:<br><br>The main question is now. Is there available access to antibacterial and antibiotics, regulation and enforcement and no alternatives again coming back to the same picture? There is no vaccines, there's no argument. So if you [inaudible] use the message there, I know that I should not use the antibiotic, but what is…<br><br>P: What is the choice? Yea.<br><br>P: Antibiotics, because …<br><br>P: Yea.

THEME: Food safety

**Food safety:**<br><br>Food safety at different food distribution channels (e.g., wet vs chill markets)	Day 1 workshop:<br><br>P: And I also thinking about here, because the Canadian model, they are only look like talking about retails, but in Southeast Asia they are many different kinds of the channel distribution. For example, like a wet market.<br><br>R: The wet market.<br><br>P: Wet market, retail market. I think wet market here in Southeast Asia is bigger than retail.<br><br>R: So should we change that node to calling it something broader<br><br>P: I think wet market.<br>…<br>P: I think that is a bigger node than retail in Southeast Asia.<br><br>R: And it is like the cost of things different and like the market versus like the bigger food.<br><br>P: Yes. And also because of the wet market, when you are talking about retail, usually they have kinds of standards, but for the wet market, they come with the small kiosk from the seller, and the small kiosk, like that, they don't, they probably buy animals from the small they just the animal around house and those are very difficult to control.<br><br>R: And is there regulation on like costs and stuff for wet markets, or is it up to the seller of like how much they sell or whatever?<br><br>P: Usually up to the seller yes.<br>…<br><br>R: And then how does that influence things, like drives, we link that but …<br><br>P: Because we are talking about the retails here I don't know about other but in [name of country] at least, the bigger retails they have standards, controlling. So the producer, you at least need some certificate, but for the wet market, because the amount of the product is not a bigger volume, so usually they will go to, go around local markets, local animals, local slaughter house, which is not in the mainstream of controlling by the government.<br><br>R: So it is negative to the regulation.

	They are not regulated by this. I am just wondering how we link it into things.<br><br>R: So increase migration increase wet market. Maybe we get into something to do with the lax regulations.<br><br>P: Could be lack of.<br><br>R: And it is maybe decrease food safety, and then increase human illness?<br><br>P: And probably cannot be traced and not be traced where the animal comes from. So that is very difficult.<br><br>R: But people, so if we try to link it to maybe like consumer demand, the people go there for different reasons than they would go to retail.<br><br>P: Because of the people [from country] still believe that even you buy, and warm meat. When they go to the market, it is kinds of warm meat. Right…Warm meat, but if you go to the retail, will be frozen.<br>…<br>So the culture of the probably Asian they don't buy the concept of the frozen meat yet.<br>…<br>And sometimes they prefer for example like chickens, sometimes they prefer to buy live chickens than just the meat chicken. That is the culture part.<br>…<br>P: And you may well have covered it but it is food preparation practices. Talking about food production, it is more than just that. It is whether people for cultural reasons eat cooked food, or whether they eat raw food. It is a large culture particularly in [name of country] and probably in parts of [name of country] as well of eating raw food. Raw meat. Blood. Raw fish, which increases transmission dynamics for chances.<br><br>P: A lot of street food.<br><br>P: Yes.<br><br>P: That maybe affect to the human health.<br><br>P: Yes that is right.<br><br>P: That leads to the hospital.<br><br>P: Yes. It all relates to the excess right.<br><br>R: So did you get them moving to human health.

	R: So the cultural factor is going to help, culture preference is the food and how you prepare food and that typically affects hygiene.<br><br>P: Yes.<br><br>R: And then from there goes to food safety<br><br>P: It will go to food safety. It will increase burden to illness potentially and if there is antibody resistance in pathogens that are in that food that will contribute.<br><br>R: We don't really have anything about like food, street food versus food chains, versus we don't have any<br><br>P: …because in Asia, especially in Thailand, street food is probably the biggest distributor of the food around countries.<br><br>R: And where they source their food from.<br><br>P: Yea.<br><br>P: Okay, raw versus cooked is one issue. We need to talk more street versus restaurants. I would actually prefer street and raw versus cooked here. Okay, because street has a high turnover.<br><br>P: Yes, high turnover.<br><br>P: And whereas restaurants, you don't know what is inside the restaurant.<br><br>R: Yea.<br><br>P: You could have fishes lying around one year.<br><br>P: Hotels are the worst you know in my experience and they have transient kitchen staff who have no training in food safety.<br><br>P: You should have stepped on the street.<br><br>P: Go to the street food.<br>…<br><br>R: Is it a cultural preference for food? Raw versus cooked. Street food versus restaurants?<br><br>P: Street food is kind of distribution.<br><br>P: That is right, but I think…

	R: But is it linked in the same way that this does? So if we have increased street food consumption, what does that lead to?.... So if we have more switching from kitchen restaurant to street meat. What does that do to the system?<br><br>P: Actually more in [name of country], there was never a culture of eating out in many states. Now because of the lifestyles and the stresses and moving, traffic, etc. there are more and more people eating out, and the street food actually has increased.<br><br>R: So could it be more a switch from home, so increase eating out could lead to more issues.<br><br>P: Yes. Yes. It changes our food.<br>…<br>P: It changes the dynamics of it.<br>…<br><br>R: I am just wondering if then if the switch from home cooking to more street food or fast food, does that then change this sort of nutritional, like worse nutrition. I don't know, I could be making, changes in microflora?<br><br>P: Definitely because there are different changes in the balance of nutrition.
**Food safety:**<br><br>Use of antimicrobial disinfectants and detergents for food safety purposes	Day 2 workshop:<br><br>P: Another part is that I am not sure, are we talking antimicrobial in a broad sense, right? Have you explored the usage of cleaning agents that they use in food industry? Disinfectant? Maybe ammonias which actually can affect, they are really used a lot and used improperly. I mean this is something that is widespread a lot, and this kind of [inaudible] don't really rings home from the food contact surface. It transmit to the product.<br><br>R: Okay.<br><br>P: And you not just eating the meat. You are eating the detergents, the chemicals that comes with [it].<br><br>P: By-products.<br><br>P: So this is actually a big issue especially in the meat industry, where, and also industries that doesn't have any pig processing. Just blend I think.<br>…<br>P: And this is not only in food manufacturing. If we go to KFC. If we go to [inaudible] things, right. So where there is meat.<br>….

	R: And some of these agents are also used for disinfection on farms?<br><br>P: Yea. Right.<br><br>R: And so if we decrease the ones that select for resistances, sometimes the genes for these are moving on the same genes for antibiotic resistance. So if we decrease these, we could decrease resistance…<br><br>P: Right.<br><br>P: Is there evidence of detergents causing this kind of resistance?<br><br>P: Yes, especially those based on triclosan, chlorine based, ammonia-based yea. So if let's say some companies might not name the name, they change their detergents, every now and then, but it does not affect the ability of the microbes. You can even alter something. It still has some microbes.<br><br>P: They have got very hard spores.<br><br>P: Yea, even on the surface, and also improper usage, in terms of the people that are doing it are, they don't have a proper understanding of chemistry. So if you tell them do 250 [unit], they will just<br><br>[Another P: I got it]. In Malaysian term it's called [agaga], they are just like that. So they didn't do that in proper scale.
**Food safety:**<br><br>Increased automation to reduce need for antimicrobial cleaning agents and improve food safety.	Day 2 workshop:<br><br>P: Another part that I would like to add on is the increase in automation in food manufacturing, especially in countries like [name of country]. We had one customer last week who want us to help with their production of the increase [inaudible], and the one thing, the outcome from that is reducing workforce. The workforce that handles all of these chemicals and stuff, so generally when you increase automation you decrease chemicals to sanitize, because the human part is [removed].<br>But then you lose jobs.
**Food safety:**<br><br>Whose responsibility is food safety.	Day 2 workshop:<br><br>P: Also in [name of country], there is something the authorities, especially for those restaurants, bigger restaurants that they are going to self-regulation. Right. So now it is very intensive, like you know they give ratings to hospitals, caterings and everything. A is good, B is… you know, on the hygiene system. So when they go self-regulation, self-regulate the system, so the premise that some must regulate food safety. Right, so this can be either way. Whether they do it properly, or they don't really, yea.<br><br>R: It is a compliance issue.

	P: Yea.<br><br>R: Yea, and sorry, was that the retailers or the food production?<br><br>P: Retailers.<br><br>R: So there won't be an overarching governing body.<br><br>P: Yea, so we are thinking about the level of, there is something like 150 foot premises of this, and there is only one food safety officer for every 500.
	Day 2 workshop:<br><br>P: Something that I would just like to discuss, always in the food industry we are talking about a lot of things that was at one conference recently was food safety ownership. People don't really care enough to do things properly. So how would that kind of fit in this.<br><br>R: I think, yea. Is that an issue of awareness and education possibly?<br><br>P: Yes. Yes.<br><br>R: Okay. So we have food safety. I have a big thing here on everything. Food manufacturing preparation, food retailers.<br><br>R: Yea, it goes right down to the individual level, like in their own kitchen.<br><br>P: The whole [inaudible] chain.<br><br>R: (name of participant) talked yesterday about the different diets, so raw versus cooked meat. Warm versus cold meat. That is all back to awareness.<br><br>P: I mean I think somewhere it was the education that, I don't think it is in the right way. They have a food meal program in schools, and they are asking the kids, I mean you know if it you know smells bad, it tastes bad, then reject it. So I don't think that is very good for kids.<br><br>R: It is not good for awareness. It is almost like shifting it down to the consumer and away from the government.<br><br>P: Passing blame.<br>…<br><br>P: 50% of our food incidents happens a lot outside of the house.<br>…<br>Yea, so one of the major things is school, it doesn't get reported so much, but we know.

	P: Yea.<br><br>R: So…I have this, like we captured the self-regulation of the food. You are getting it even broader. It is like the government is pushing down the responsibility, so that is kind of reflected in self-regulation and retailers…<br>…<br>P: Yea.<br><br>P: It is like …<br><br>P: Trust you have to go by trust, but very little, I would say the trust bank is very low.
	Day 2 workshop:<br><br>P: Yea, I think in [name of country] it is a particular issue, because the level of cleanliness can change yea I mean with different requirements. Nobody wants to go to the kitchen and see what is going on, yea, but it all depends on yea.<br><br>R: Is that at every stage, like retailer down to farm?<br><br>P: Yea. We have a huge immigrant population, yea, because it just lack labour you know to do these things, and we are not willing to pay the price to get good food on the table, and I think yea I mean generally we externalize the cost for cleanliness, environment, health. We externalize the cost. The government doesn't internalize the cost. If you really internalize the cost, similarly with pesticides you know if you really internalize the cost of production, then whatever food you produce is actually very expensive, because if you got to pay for the environment, if you are going to pay for [food] safety, it is going to be very, very expensive, but that is a political thing. They don't want to do that, that you know everybody is happy, yea at the end, I want to go for a very cheap meal, and so the price keeps going up, but the sad thing is where people really care for good food production, like maybe some of these organic farmers, then you really internalize the cost.
	Day 2 workshop:<br><br>P: Yea. You see one of the, if you look at a more developed economy, compared to our developing economy, one of the key things is everything is sort of the pull factor is price. What can people afford, yea? And everybody wants cheap food and therefore they don't want to pay for a lot of you know from the cost of labour to setting up systems which you know sort of underscores cleanliness and so on.
**Food safety:**<br><br>Food preservation	Day 2 workshop:<br><br>P: I think the same on this issue. What I look at is here around the world, I can put with this, what you call it, expiration date, you know best before. I rescue food, you know, supposed to be before expiration dates, we

	donate and we check again the date of expiration before to people who are in need, but even a piece of brown [inaudible] from an airline which we rescue and left two days ago, I kept it for another month and send it to the lab, it came up with passing mark, so the problem is the expiration date that is on whether canned food or packed food, are basically the manufacturer best guess, you know, and whatever costs, on the cyclic of the food written, it is still billed back to the consumer period, as a business, you know. So this needs to be, something needs to be done. There must be some regulation or body to tell me why your canned food only can last one year, you know, while when we check, even I kept another year it is still good, you know. So this cause a lot of wastage, because we are so obedient, looking at the dates.<br><br>P: It's coming back to food labelling, there are specific and very clear guidelines on how to set expiration, best before, and when it expires, so they have clearly labeling requirement.<br><br>P above: I'm saying we need to narrow down. I am not saying you should not put a date.<br>…<br>P: Maybe some bar chart or whatever, where people can read, best before doesn't mean tomorrow you consume something happen, but we as consumers, we would not want to attempt to eat the next day, you know. We forgot as a human we have the ability to detect rotten food. That is it, you know. We can buy fish from the market and try to choose the freshness, you know pull out the gill, press the eyes and you know we can do it ourselves. When it comes to canned food, we just so obediently like our hand phone. I don't even know why hand phone, just press down, you know. So it is costing massive waste out there. [R: yea] So I am not saying that. We need to narrow down, to be certified that the [bad food] cannot be doubled, in a sense you know because just on my study, just [name of a company] alone, [Name of company] in [name of country] is a few billion revenue on mostly pack[aged] food, you know. Chocolate, instant noodle. This 2-3% of food being returned just because it is close to expiration, and you can't sell anymore, and it land in the landfill. 2-3% of good food inside is still good to eat. It is just because they make it cannot eat. It does not make sense, and it is a few hundred million being thrown you know, right? So something needs to be done by authority. Don't leave it to even certification on hazard and so on. Some authority higher than that, that you have to show proof you know, your test, that this is one year, this is over some messes, in that sense. So this would save a lot.
	Day 2 workshop:<br><br>P: I think agree. You see technology in all expect from a mobile phone apps, you know GPS technology, transportation, non-invasive surgery, we are still in the dark age as far as preservation of food. Something needs to be emphasized on that. It is not just the antibiotic. We are still very far behind. Everything is on it too. It change the way we live, you know. Food

	preservation is still the same thing, soft preservation, additive, nitrates and things like that. Nothing like a scanner, you can scan everything. It can last like that. Then we may not need antibiotics. We will process the meat using that system, you know. You can extend three years. So far it is very, you know I know culture, [inaudible], you know and the rest are preservation you know. There is nothing that happens over how many decades, we are still in the dark ages.

THEME: Population growth and migration

**Population growth and Migration:**<br><br>Increased crowding, demand for resources and food products, and access to products via different distribution channels and associated impacts	Day 1 workshop:<br><br>R: We had one follow up on the increased population, leading to migration, but I think you created some new nodes, but it does link into consumption of other non-meat products consumption…	
	Day 2 workshop:<br><br>P: I think we are adding something like 90 million to 100 million to the population every year. Yes, every year. Yes, every year.<br><br>R: Population growth.<br><br>P: So you will be ready. You don't medicate. [If] we [don't] clean up our mess, I don't think, you know, you will balance back, you know.<br>…<br>P: Also, but yea so I think that one tries identification of agriculture is yes, population growth. We are more people with more and our consumption patterns, our diets that are also changing you know to the worst, consumption of more meat for instance, so there is much more demand for meat, and it is forecasted to be growing, and these will lead to expansion and intensification of livestock production with the consequence of using more and more antibiotics if we follow the same pattern.<br><br>R: So it is not only population growth but also a change in diet that goes with it. Change in lifestyle… and population demand, and increased consumption…If we decrease food waste, would that mean there is an increased amount of domestic product on the market.<br><br>P: Say again.<br><br>R: If we decrease food waste, would that mean there is an increase in domestic product, because we have that line over there. Decrease food waste.<br><br>P: Because at this currently from farm to home, we waste a good 40% of food, right, which is not necessary because of our consumer behaviour.<br><br>R: And consumer preferences.<br><br>P: Yes.<br><br>P: Because if we won't eat something that doesn't look beautiful, it is wasted. So if we decrease that part in the farm to fork, we would increase the domestic availability of food.<br>P: Availability is there to match the population.	

	Day 1 workshop:<br><br>P: We are talking about every country is facing the same thing. Increased demands, decreased resources, crowding and migration. Migration may not be migration between countries. Here is always migration happening to urbanization for example. All this is actually affecting hygiene, transmission of infections and bacteria, water resources. The whole thing it gets mixed in with that kind of a macro issue also.<br><br>P: So migration is part of the bigger.
	Day 1 workshop:<br><br>R: So you said migration. We have got crowding on here. Something that happens and the need for survival, the drive to survive.<br><br>P: So you also have an inverse action on business models with more population, prices may come down or demand of certain products may go up, especially as you move from a rural to an urban lifestyle. Let us put it that way. Maybe from veg to non-veg, that is why migration becomes a component actually.<br><br>R: So how - link the population to population demand? Is that what you are saying?<br><br>P: Migration is a component of. See it is when you have an increased population and you can't get a job in your own place, probably that you move.<br><br>R: Absolutely. So the existing population and migration can create population demand for product…but you said something else.<br><br>P: It impacts more resources. It impacts food safety. It impacts the sanitation. It impacts even the, for example, when you have an increased population, there are not enough resources. You tend to, let's say if you don't have wood. I will give you a simple example. You could probably eat something which is different to what you normally eat for example.<br><br>R: Okay. So this would all …<br><br>P: And it does affect and impact the food chain also indirectly.<br><br>R: Yea.<br><br>P: So I think when we are talking about migration, right. So because it is Southeast Asia, they still have livestock movement around the border as well. So that is probably not talking about this.

	R: So with migration of people, there is migration of animals, livestock. Is that what you are saying?<br><br>P: Yes, because they are trading live animals move around, so that might affect two or three things. One is the kinds of diseases and the other one is maybe just move around of the AMR around the regions.<br>Because of the, for example like [name of four different SEA countries], they move animals around. Sometimes they move the animals from China to migrate.<br><br>…So and often these animals are transported on long distances under poor conditions. Sometimes carrying viral diseases like foot and mouth disease, which I have seen.
	Day 1 workshop:<br><br>P: So you have got this issue of, for example if you go to many of the big cities, whether it is [name of two big and highly populated cities] or whatever. You have got huge pollution issues leading to respiratory infections. That leads to health seeking behavior. That leads to antibiotic use. Then you have got water scarcity issues. That leads to drinking, whether it is in a village or a city. That leads drinking difficult water. Let's put it that way. That leads to diarrhea. That leads to again health seeking behavior.
	Day 2 workshop:<br><br>P: … We demand perfect food, and want it cheap and so on. And on the lifestyle, the fast food, the convenience store frozen food and all these things we have created convenience is all come from this people, and the population is growing…<br><br>What I know is it is all created from evolution and so on, the lifestyle, the population, and that creates, things become very commercial. We need to produce at the speed and everything commercialized, packaging material and so forth, and we abandoned organic farming you know. In that sense, that to speed up, because our rejection of imperfect food we need to, farmer has got to plant more to sort of offset the rejection. So from the farmer, there is a lot of losses and so forth. It is all in the name of profitability. This is what is happening, and with this, when it comes to production, to do it we need to administer like more antibiotics, herbicide, pesticide, whatever, so that you can get a better yield, you know, and this component is basically here what we are looking at.
	Day 1 workshop:<br><br>P: And I also thinking about here, because the Canadian model, they are only look like talking about retails, but in Southeast Asia they are many different kinds of the channel distribution. For example, like a wet market.<br>…

	P: Yes. And also because of the wet market, when you are talking about retail, usually they have kinds of standards, but for the wet market, they come with the small kiosk from the seller, and the small kiosk, like that, they don't, they probably buy animals from the small they just the animal around house and those are very difficult to control.<br><br>R: And is there regulation on like costs and stuff for wet markets, or is it up to the seller of like how much they sell or whatever?<br><br>P: Usually up to the seller yes.<br>…<br>R: And then how does that influence things, like drives, we link that but …<br><br>P: Because we are talking about the retails here I don't know about other but in [name of country] at least, the bigger retails they have standards, controlling. So the producer, you at least need some certificate, but for the wet market, because the amount of the product is not a bigger volume, so usually they will go to, go around local markets, local animals, local slaughter house, which is not in the mainstream of controlling by the government.<br><br>R: So It is negative to the regulation. They are not regulated by this. I am just wondering how we link it into things.<br>So increase migration increase wet market. Maybe we get into something to do with the lax regulations?<br><br>P: Could be lack of.<br><br>R: And it is maybe decrease food safety, and then increase human illness?<br><br>P: And probably cannot be traced and not be traced where the animal comes from. So that is very difficult.

THEME: Awareness and understanding of AMR

**Awareness and understanding:**<br><br>Farmers	Day 2 workshop:<br><br>P: Farm biosecurity: many small-scale poultry farms as a traditional farm without farm biosecurity practices. They are also limited knowledge on farm management, medication program and lack of antibiotic knowledge…
	Interview A:<br><br>because you know you have got remember with the level of education, particularly in small farmers, they don't know what they are saying, you know they build up a sense of the story, and patterns, but we were often finding where there were FMD (foot and mouth disease) outbreaks that they were using antibiotics differently. We find a whole lot of empty bottles of gentamicin for example. So, they don't necessarily differentiate between the different antimicrobials and the agents.
	Day 2 workshop:<br><br>P: I would agree with what you said. I see from the authority, all the way to the farmers, they don't understand the consequences of that.<br><br>P: Yea.<br><br>P: They don't understand it. They don't even know what is an antibiotic.<br><br>P: Yea.<br><br>P: They don't know why they use it to treat disease. So…<br><br>P: Yea. Exactly. Even clinical doctors, yea. So but I am looking at it from the farming perspective from farming in terms of profit. In terms of animal and livestock. I think it has been used in livestock for quite some time, but for us (crop sector), it is new, because I think people are trying to use antibiotics because of this resurgence of bacterial diseases, exotic bacterial diseases and that is not helping.<br><br>P: Livestock officials are the same.<br><br>P: So it is not helping.<br><br>P: The concept of AMR in [name of country] workshop, it looks like it was very complex about the specific cause. We have farmers in the room. We had sixty participants and it was difficult.
	Day 1 workshop:<br><br>P: The other thing is also the sharing of experience between farmers. That is a very important source of information. So if one farmer has had good

<table>
<tr><td></td><td>success with one drug, they tell their neighbour. So even if that makes a different problem. They say like, oh they use whatever, penicillin or whatever it was called or just this new drug, that they now sell in the drug [inaudible] and I buy this one. So there is this really poor level of knowledge about what antibiotics are and how they work.

P: They trust their neighbour. Yea who has good experience in that case that did work.

R: So this all influences antimicrobial use on a farm.

P: Yea.

R: And the training will influence good farming practices and terrestrial use or user, just talking straight …

P: I think both. Yea

R: Okay, and this is all driven by lack of resources, lack of education.

P: Yea.</td></tr>
<tr><td rowspan="3">Awareness and understanding:

Food chain actors</td><td>Interview A:

P: wet markets for animals – a high risk area for stressed animals shedding high levels of faecal bacteria and AMR. I can't stress enough the major issue of informal and inhumane slaughter, even in 'formal' settings, in SEA – it is truly a hot bed of AMR risk for workers and food systems – aside from cruelty, which is documented for various zoonoses also. A lack of knowledge, training, standards and regulation persists….</td></tr>
<tr><td>Day 2 workshop:

P: Something that I would just like to discuss, always in the food industry we are talking about a lot of things that was at one conference recently was food safety ownership. People don't really care enough to do things properly. So how would that kind of fit in this.

R: I think, yea. Is that an issue of awareness and education possibly?

P: Yes. Yes.

R: Okay. So we have food safety. I have a big thing here on everything. Food manufacturing preparation, food retailers.

R: Yea, it goes right down to the individual level, like in their own kitchen.

P: The whole [inaudible] chain.</td></tr>
<tr><td>Day 2 workshop:</td></tr>
</table>

	P: I am looking at my perspective as educating food waste in this country, like what I know is we actually are practicing a very rotten food system locally, you know…<br><br>but I think while we are doing this and me doing the food waste, we need to educate you know in school in my eleven years in secondary or primary school, nothing was taught on all these shocking, you know. We learn to as we move on, but we can inject education everywhere, even from small, you know something that people know and learn to respect food. How food comes about. How much resources has been wasted. You know how much of this thing affect our health.<br><br>This is like, it is all known even Americans have now been running a 42% of American are having cancer by the age of seventy-two you know. Fifty years ago cancer is unheard of you know. In my country [name of country], it is 25%, and as the country developed, because you demand [inaudible], percentages will go up, so I think education is important for us to do that. And the food wasting issue, because of our demand lifestyle, what would produce and we know from the people here obesity, five hundred million around the world. Even in my country, [name of a SEA country] we are among the factors in this region, compared to… [name of three other SEA countries] and so it is happening. We are basically copying the American model, as we progress [economically], we get larger and so on, I don't think taller. [laughter] So I think we need to inject education everywhere into people. While we start to do all these things you know, because all these also affect climate change you know. Food waste in the land fill emits methane gas, you know, and the resources, the [inaudible] field that need to go into agriculture. It is all lost, you know, the arable land, the water, and all this affects you know, so I don't know where to put these, you know. I think it is important that I do my part, you guys have to do your part. The population will catch up with us if we educate. So there will be reduction.
**Awareness and understanding:**<br><br>Policy makers	Day 2 workshop:<br><br>P: And they probably won't work anyway. [P: they might not work] So actually making vaccine is the easy part. The hard part is how to convince [inaudible] like in [names of 2 SEA countries], they don't have lots of vaccine, because they just make it very hard.<br><br>R: To get them to adopted?<br><br>P: Yea, because they need approval.<br>…<br>P: We need to educate the policy makers also. Yea.<br><br>P: Yea, educate the policy makers.<br><br>P: Give them a master class in vaccine production.

	P: And that it is safe. They have the misconception that is not safe. [P: exactly] We see it with humans, with kids, those crazy antivax.
**Awareness and understanding:**<br><br>Prescribers, front-line workers	Interview B:<br><br>P: There is nothing on the health care provider (physicians, pharmacists) but they are important because they have an influence on prescribing drugs or dispensing them. This has an impact on use (appropriate/inappropriate).<br>…<br>The real front-line works and patients have insufficient awareness about this issue and they need to be aware to ensure health and well-being.
	Day 1 workshop:<br>…<br>P: …Okay so that is step one. The major one which I would like to talk about, which we talked about in the last couple of days is diagnostics access to antibiotics and making a diagnosis in the first place of what the infection is and testing that for antimicrobial susceptibility.<br><br>P: Can I intervene<br><br>P: Please.<br><br>P: So one of the big problems when you say the word diagnosis, if the young kids on the block do not know their approach.<br><br>P: No.<br><br>P: They are not taught the approach.<br><br>P: No that is right.<br><br>P: So for example, sorry microbiology or pharmacology we are taught modelling of a bacteria, put it this way, it like this and it like this. We are not taught about what it can do and how we interpret the culture and sensitivity or how to approach when is a gram positive or a gram negative, etc. etc. So what happened and just before that or that actual lab result, and we were talking about this earlier, two days back, most of our teachers would concentrate on the history and get a preliminary diagnosis just by listening to the patient. Just by listening, and then you examine and confirm what you think and then take one or two tests and get the diagnosis.<br><br>P: Today super specialists and beyond they do the opposite. They know how to tick all these dots, but they go by exclusion. So this is especially very important in low and middle countries, where we can say all we want, let's have this many diagnostic facilities, this many equipment, this many trained personnel, but we know it is a long run. So we have to

	improve the approach, teaching and the interpretative and problem solving skills, as well as developing clinical algorithms, which help those physicians to assimilate that data they get from the patient, and sort it out in their mind.<br><br>P: Absolute right, and that is why I use the term stewardship. It encompasses all of what you what you succinctly put very nicely stated. It is not just the availability of services. Ultimately it has got to be holistic process involving training people to take the right sample but interpreting the result at the end of all of that, then it goes around in circles.<br><br>P: And you can make that link to livestock as well.<br><br>P: Exactly.<br><br>P: Same thing with officials. You see a lot of farmers every, nobody talks of clinical sense, and nobody talks of pathology. Nobody talks of how they function in the process they lose everything and if they got the disease. Provide us , there is nothing. The animal is happy, smiling, so that is all because of their availability and access to modern diagnosis and running away from pathology<br><br>P: I think so.<br><br>P: Unfortunate. Very unfortunate actually.
	Day 1 workshop:<br><br>P: Yea we are talking about everyone actually. Even… even we are talking about, we have a situation in [name of country] currently, where people are reaching a super specialty level, because the entrance exam only tests knowledge, okay [inaudible] otherwise, but they don't even know how to put in an IV line, because they have been studying all the time… So that sounds like awareness can even be in a super specialist about a field they don't know. Somebody goes to a cardiologist. Something that is hypertension, but they say no, I have a cold. Can you give me medicine? Probably given antibiotics
	Day 1 workshop:<br>…<br>P: We can put it as part of stewardship and diagnosis, but it would be good to have one node somewhere, a theme somewhere about education learning and training in all spheres, because where there is a farmer, or whether it is the vet or whether it is a doctor, have many of the population just go to the and they have probably not been trained enough to recognize but they are given something.
	Day 2 workshop:<br>…<br>P: And then because sometimes the decision is made by probably the farm, you know, the owners.

	R: The farm owners.<br><br>P: And probably some kind of guidance, from the veterinary, you know, sort of doctors or somebody who may not be having a full understanding of, you know, a complete understanding of the (antibiotic) product. For example, if your product is not very soluble, then you might have a lot of residues floating on the water, I think there's some, you know, publications, where they are told that the resistance is mainly because the drug which is floating is undissolved. It has been consumed by the fish and you know this kind of thing. Then probably, that might also lead to, you know, resistance for the drug.
	Day 2 workshop:<br><br>P: So people are very selective about it. Write that down and you know back and also clinical doctors don't usually very silly, I would think you know, because the first thing they do when you have a flu is to say, I think you better get some antibiotics. It is silly yea.<br><br>P: Yea.<br><br>P: Antibiotics don't work for viruses.<br><br>P: Especially this region.<br><br>P: Exactly. Yea, I had it in [name of Asian country]. I was in [name of Asian country].<br><br>P: I am not saying it is not, it is greater than other regions.<br><br>P: …because they think about prevention. They say oh you have got a virus flu. A couple of days later, you will have a bacterial infection coming on probably. Therefore, you might as well take it, yea for the next five days…It doesn't work with flu.<br>…<br>P: Oh I had a big problem. I studied in, I was in Japan, yea for a number of years. I studied there, and the first thing the doctor did was prescribe antibiotics. I said what the heck you are doing you know. This is wrong. Yea, but oh yea as you say, patients, yes sometimes. So the prize factor, differential I say negates the use. So if you put a premium on availability, in terms of cost, price, then I think it sort of negates the… how would I say the… impact of you know use in terms of extended use whatever.
