# Supplementary material for: Factors impacting antimicrobial resistance in the South East Asian food system and potential places to intervene: A participatory, one health study
Source: Front Microbiol. 2023 Jan 5;13:992507. doi: 10.3389/fmicb.2022.992507 (PMC9849958; doi:10.3389/fmicb.2022.992507)
Supplement: Supplementary file 1 [file Data_Sheet_1.zip › Supplementary File B.pdf]

SUPPLEMENTARY FILE B: QUOTES PER OVERARCHING FACTOR

Acronyms:

P: Participant
R: Researchers that facilitated discussions
SEA = South East Asia
AMU = Antimicrobial use
AMR = Antimicrobial resistance

Please note:

1. Some quotes were modified in instances to protect the identity of participants or the organizations they represent.
2. P: represent a participant. Some quotes contain multiple P's. This means the quote reflects a discussion thread involving a participant and the researcher or multiple participants.
3. A given quote may be coded in more than one theme if it applies to each.

OVERARCHING FACTORS

**Drive for Survival:**<br><br>Desperation due to conditions in SEA	Day 1 workshop:<br><br>P: So to me I would say, divide environment in water and soil and then for water you get antimicrobials getting into water and then again people getting exposed to antimicrobials through drinking water is one thing. Then you have also people getting exposed to antimicrobial resistant bacteria through water. So there is another arrow that connects water through resistant human infection to resistant human infections.<br><br>R: I did already do that one.<br><br>P: Okay and the same with the rest. The same with all water users' as she said, but it is already linked and fish. So aquaculture.<br><br>R: Yea. I got it.<br><br>P: To antimicrobial use. So this type of this<br><br>P: Connect environment back to humans. So you have got this issue of, for example if you go to many of the big cities, whether it is [name of city] or or whatever. You have got huge pollution issues leading to respiratory infections. That leads to health seeking behavior. That leads to antibiotic use. Then you have got water scarcity issues. That leads to drinking, whether it is in a village or a city. That leads drinking difficult water. Let's

	put it that way. That leads to diarrhea. That leads to again health seeking behavior.
	Day 1 workshop:<br><br>P: …and the last point I want to bring in was survival. That is also a key thing. I mean we are talking about, this is where policies may differ. No policy is going to judge any farmer who is existing for survival families' sake and forsake. They are already in debt. Nobody is helping them. Yet climate change is affecting everything. There are pests and insects. All that they just have to survive and they have limited space, crowding, you basically say don't crowd them, but they will crowd. There is no space left.
	Day 2 workshop:<br><br>P: Everybody… you know farmers are desperate, the countries are desperate. Because…with pest control, yea, …find very little solutions for bacterial related problems. And so the only way is either you use a sort of a curative action, in fact going up to even using soap solution, you know, J-binds and whatever, plus antibiotics, but then now governments recognize that you know antibiotics that is not the good way to go, because of this resistance issue, and therefore they have sort of now coming out with these action plans, but what is the…what are the solutions that they are providing, you know to, to [inaudible] us for example. And so in Florida almost 90% of the trees are affected now, even though you know we thought America will come up with the solution, but now they are very worried that it will fly to you know, because it is spread by a vector, and so they are worried that it will go to the California citrus industry, and Australia is [berserk] you know, because Australia has got a very big citrus, so export market and when the industry is very, very crucial.<br><br>P: So from the food security angle, and also food security in the context of bacterial diseases affecting food security crops, like rice. It is a very serious problem. Yea, we don't have a bactericide I would say. Similarly we don't have a virus-cide, [inaudible]-cide we don't have. We have a lot of fungicides, but not bactericides.
	Day 2 workshop:<br><br>P: …[plant clinic] don't encourage people using antibiotics you know when…give recommendations…. [R: what clinics?] Plant clinics, just like human clinics and you know farmers in many countries recommended antibiotics, because the major problem with antibiotics is you know controlling bacterial diseases, it's a… how is it…it is the Holy Grail. It is a major impediment for crops, yea? Particularly rice. So farmers tend to resort to you know desperate measures I would say.<br>…<br>…[Plant clinic] go by good agriculture practice standards so…encourage [farmers] not to use, although there are a lot of desperation.

	Day 2 workshop:<br><br>P: The whole problem is a lot of people are not sure what is happening. Everybody is looking at a sort of a curative mode of, you know, action, when you have a desperate problem. So I just give you an example, yea. Why we form this technical committee in order to address this issue of antibiotic use, simply because two things in agriculture are very important. One is from the food security angle, which you have addressed, because food security meaning that we worry about bacterial diseases okay. I can talk about [name of country] and also maybe the countries in the region. Rice has got a major problem with the bacterial diseases and the only way we can prevent them is by resistance development and that is a long drawn out process, and pathogens evolve. So you get screwed up, you know. So we tried all sorts of preventions for bacterial diseases or curative infection.<br><br>So food security is a big issue. The second issue is markets. Our horticultural products you know. We lost the papaya industry. We are losing the papaya industry. We lost the citrus industry, and we were very happy when Florida got the citrus greening problem, because we thought that would be some… [group laughed], happy in the sense that I am sorry, but not happy in the, not in the wrong sense, but happy in the sense that we know that there is going to be a lot of fundamental research going into it. America will support this. USA is very far ahead, but still they have not resolved the issue.<br><br>So in 1998 we had this what we call the [name of place] [inaudible]. They wanted to plant 500 hectares of this very sweet orange and it was a great export potential, but then this citrus greening came, and citrus greening is like HIV. Once you get it, it is very difficult to remove from the system, because it is a bacterial disease and if you could find a solution to a bacterial problem today in agriculture, it is a Holy Grail. You are a multi-millionaire. You make money.<br><br>So people come with all kinds of combinations, you know in terms of trying to use bactericides, you know antimicrobials. So what happened was people were, this citrus greening came to [name of country] in 2000… around 2004, 2005, and it sort of wiped out this [name of place]. Yea, sort of wiped out. It slowly sort of depleted the whole industry, and that is why we came and we got worried, and there were also a lot of products, agricultural products coming in which were like you say growth enhancers, you know people were modifying bacteria easier to modify by technology modify some of this, and transfer this bacteria and this was also a big concern about what happens if there are conjugated strains, and you know all these hybridization between bacteria.

	So it is from the soil, from people, the problem. There was one professor from [name of country] went there, very serious problem with the citrus greening. [Professor] actually came there and gave recommendations for our citrus farmers, especially this honey melon and also watermelon. Not watermelon, this pumelo, which is a big industry…to inject and that was a real problem. America doesn't do that. America sprays. They spray, so but it is a bit gray in terms of that, they started Injecting. So we got worried you know when you start injecting, because the reason for injecting is this particular bacteria affects the physiology system of a plant. And therefore, it blocks, it is like a heart problem you know where you have cholesterol in your heart. Your arteries get blocked, and therefore the whole plant slowly dies. So they started injecting.<br><br>Of course it worked for some plants, but it didn't work for a lot of things, and that concerned us, because of the physiological systems, you know going into the soil, the water system, lab. So this is where the issue came, and until now we have not resolved the problem, although we have given recommendations how we could minimize the use of these antibiotics, but still you know the citrus industry is gone. So in that sense our export is gone basically.<br><br>R: That affects market.<br><br>P: So yea, that is a market factor.
	Interview A:<br><br>P: you know changing gut flora and consumption of the foods, etc. Increasingly you know we know that we are seeing sort of endemic aspects of contamination into the food system, and so I guess how that is going to play out…So yea there are other things that might change to some degree and relate to food security,
	Interview A:<br><br>P: I think many of the nodes there, though I would think particularly in this region, one that is of key importance is contagious viral diseases, infectious viral diseases. Obviously you know things like African swine fever, bird flu, and I would think that they are really quite a predominate node in the sense that they, while they may or may not have direct impacts, they obviously because there is no treatment for these diseases per say, they do influence systems significantly. They influence markets and trades significantly, and the really obvious one at the moment is this African swine fever in the pig industry.<br><br>P: But also we now see as .. that impacts the industries that support it, but also it impacts the poultry industry and the fish and aquaculture industry as well, as people shift and as industries shift, and so a node about I would think about not only the systems per say and illness,

	because you have got production and upfront production, but the geography and trade of products is a key one…<br><br>R: The contagious infectious viral diseases one… do you see that as sort of something that impacts the whole system?<br><br>P: Good question. I think probably in some ways both because contagious infectious diseases you know obviously can ultimately be suppressed, so for examples in Indonesia, High Pathogenic bird flu, HPAI is still in existence and they consider it now sort of endemic, but that might also mean that the immunity of flocks is reduced and therefore they are more susceptible, vulnerable to bacterial diseases as well or complexes which have a viral bacterial and management interrelationship, and therefore that might lead to more use of antibiotics. For example, or other impacts related to their system, and then as I was saying before these diseases also can have a shift on the overall situation and systems, because they might push it in one direction or another.<br><br>P: So I think it is probably a combination, and then I guess even if it does, additionally if it is zoonotic, like you know bird flu, then again that might relate to inadvertently increasing treatments needed or preventatively for humans and could contribute to the resistance patterns as well.
	Interview A:<br><br>P: pandemics could also be a driver of AMU (animal or human…).
**Drive for survival:**<br><br>Profit driven	Day 1 workshop<br><br>P: So there is the social, psychological side, which is not being really touched yet, which includes things like culture and beliefs, which includes awareness and knowledge in one sense, which includes greed and influence. It includes even things like survival. Survival of the patient, survival of the animal, survival of the farmer, which can drive you whatever you need to policies.
	Day 1 workshop:<br><br>P: … Somebody goes to a cardiologist. Something that is hypertension, but they say no, I have a cold. Can you give me medicine? Probably given antibiotics and we also mentioned in that same section, profit, greed and influence.
	Day 1 workshop:<br><br>P: Yea and the terms for the [government] service providers, they sometimes also sell the drugs themselves. So it is actually an important, so it is like a pervert incentive [R: yea] for them to sell, because that is often their only source of income, so they cannot charge for the service, like you know in West world, that actually your knowledge, your expertise has a price.

	…<br>R: It is just the sales.<br><br>P: It is just the sales. So while they actually do [inaudible] your knowledge, at the same time there's incentive to generate income.<br><br>R: And there are two things there, and I think [researcher name] has got one. If you profit from the sales of antimicrobials, it is going to drive antimicrobial use up, and may lead to poor antimicrobial stewardship.<br><br>P: Yea.<br><br>R: But I think there is both the profiting, but there is also the linkage between prescribing and dispensing, and it can be, if you both prescribe and dispense, there is a conflict. Even if it is the right, you still have that conflict. Whereas if they are separated, you have to go from the doctor to the pharmacy. It can possibly lead to better stewardship and use.<br><br>P: And ultimately out the black door.<br><br>R: That is true I think not just on the animal side but the<br><br>P: aquaculture<br><br>P: It's exactly the same.<br><br>P: This model develops in the right [case] especially in this part, but with follow-up extension service coalition is so deep in terms of sales. Sales of [inaudible] Sales of feed, sales of the company chemicals, that is the one that is fueling need.
	Day 1 workshop:<br><br>P: We should try to balance the role played by producers and others in the value chain in promoting the use of AM and pesticides. It is not just producers. Producers are also pressured by others – feed companies, chemical companies, procuring companies, lenders, etc.

OVERARCHING FACTOR: Leadership Priorities

**Leadership Priorities:**<br><br>Education and health	Day 1 workshop:<br><br>P: We have got one more area. That is the macroeconomic and the microeconomics. Now if you notice more and more governments are moving away from spending money on education and health or some governments are moving towards privatization, with its own pluses and minuses. So when you decrease your efforts on education and health, Canada maybe not in this so far, but the rest of us are, what invariably happens is that you, it has its knock on effects on nutrition, nutrition education in schools for example or even free mid-day school programs with kids and that is a very important thing actually in many LMIC. It has knock on effects on whether teachers actually teach approaches to life rather than teaching subjects, because most of the teachers are paid to get them through the exams.<br><br>You have got the health issue directly right and when you have less budgets, some of the knock on affects are on farmers and vets with hospitals, you know all that stuff is happening. So that is a macroeconomics of it and we haven't even really, we are not talking about war zones here. It is normal people. War zones are even worse. Right. Then the microeconomics of it, the trickle down effect with movement to the right in most countries, okay, where private reigns supreme. They are beginning to influence either directly or indirectly on policies. Okay. In [name of country] for example, very interestingly education lists were currently the counsel of [name of country]. Now it is corporate career professionals who are on the board for example. So that movement to the right invariably affects most of these things.<br><br>R: So two points there. The first one the macro It is more shifting priorities in macroeconomics.<br><br>P: Yes.<br><br>R: Away from education and then with the micro.<br><br>P: It is a trickle down effect of that.<br><br>R: Of the macro.<br><br>P: Yea, but also the other factors making poorer, more poorer…And they are 80% of them probably are agriculture, because it is their work. They probably don't have the skills to or the are in a rural setup that they don't have anything else there.<br><br>…So that brings us to the other tangential point on that and that is policy makers, what are their interests and what are their influences and what is

	their behaviour and how does the leadership get chosen in many of our countries and so we have very few, because finding the leader and whether it is the village or the city or the town or the country makes a difference. Yea, so you have got for example, where there was not one not even one because the [name of leader] was a bit of a tyrant. His son overthrew him in a bloodless coup and what you see in [name of the country (not a South East Asian country] today if you visit it, there don't have much oil actually, but whatever resources they have, he has actually given it to the people. So that is one of the number one country where women have more jobs than men for example. So that brings to the point at every level in this chain also, whether it is a farmer leader or a union leader, or a professional association among doctors or pharmacists, the leadership training awareness and empathy to the whole issue matters.
	Day 1 workshop:<br><br>P: … health care professionals and health care facilities, because the health systems and its issues also affect this. It is not just the health care professionals…So you may have government systems, which are [inaudible] private coming up. They are charging more for example and therefore affordability issues, lack of diagnosis, the health systems itself is a major, the way it is changing in many countries is a major issue.<br><br>R: So question then. So the type of health system available, we have more private health care systems. What does that do to use?<br><br>P: So [an individual] did a study once where for example all the primary health care facilities run by the government stocked only cotrimoxazole . Right? So that is one side of it. Okay? On the other side, you have all the private hospitals and clinics selling only the third generation cephalosporin because that is where the profit is, and currently might be meropenem and might be even posted. Okay?<br><br>R: So it could be either.<br><br>P: Yea, we're saying, it is not a hospital. I am talking about a clinic and they give an IV meropenem to outpatient. I am not kidding.<br>…<br><br>P: But the… the complexities of the health system is now changing the dynamics…So you have insurance coming in on one side. So the insurance it is pushing. Right. Then on the other side you have the affordability angle. Okay. On the other side, you have lack of resources of beds or doctors or nurses. All affects this actually. Not enough, not enough ICUs. Right. If you don't have a bed and all you have are very busy, crowded OPD, outpatient department. You have infectious disease people. So, infected people, infecting each other, and then of course [they can acquire hospital] associated infections…

	P: Hospital acquired infections, the role of health facilities in acquisition of infection and antimicrobial resistance.
**Leadership Priorities:**<br><br>Decisions to grow foods unsuitable to the environment for economic growth and to grow foods for food security purposes	Day 2 workshop:<br><br>P: I have a question. Are some of these issues because and are people looking at the fact of like suitability for growing regions? Like trigger when you said like growing citrus in [name of SEA country], which has a very, like not too dissimilar but a pretty dissimilar environment than like [name of places in a non-SEA country], and they had all these problems. I am wondering like how much research is going into or like how much do people care about like suitability of a crop for an environment. Like… it seems like that could, just overall make the crop healthier. Like people grow tomatoes in [name of country] too, but have to use like so much pesticides to grow them, because the environment is not exactly suited, but they can do it. It is profitable.<br><br>P: So when we choose locations for growing, of course it is basically looking at economics of scale. Of course the market factors you know. It is all going back of costs. In [name of country] of course we choose locations based on certain agrological zones. We have identified them, and of course you can't stop growers from growing because if they find that something is very profitable, you know, they have got markets and therefore they grow. And of course they specifically selected this valley because this particular state produces a lot of oranges, used to produce. And oranges are sub-tropical you know, you need the sweetness, you need the dry period and so on. So everything was working well until this disease came in 2004 when they initially spotted it. By the way exporting quite a good amount, but unfortunately no, they were not able to sustain. Now they don't talk about it anymore very much. It is an industry. Yea.<br><br>R: We would probably use a lot of antibiotics and a lot of heat to grow them in Canada.<br><br>P: Right.<br><br>Group: yea.<br><br>R: So I think it is a very important point. I think it is the suitability and I would actually probably say it is not just crops, but I'd say food production and the environment. Because in some cases for us to raise, say warm water fish in [name of non SEA country], would also be equally unsuitable.<br><br>P: I think one of the sellable points is you know in terms of looking at this, just not from the technical point of view, but more in the socioeconomic positive whatever where the governments listen when you really have the social impact, because it sort of affects communities you know. People who have been given so much of hope. You know the first two

	years it is so beautiful. You know you are exporting. You are making money. There were reports. If you look at our reports, oh yea they made a lot of money. You know they were from people earning, you know $1,000. You know they were earning $15,000, and all of a sudden this thing comes in and it impacts communities, whole communities, you know? So that is the selling point in terms of pushing this agenda as much as, you know, from the health perspective of course, yea?
	Day 2 workshop:<br>…<br><br>P: The same thing with the effective microorganisms, you know in agriculture, that one of the other issues that we were examining in our committee about the effective microorganisms, because they want to increase the soil microflora, you know so then there is an issue, because a lot of people got excited about this, you know because the soil is the problem where everything starts with the soil, as much as for human beings and animals, it starts with the gut. Therefore gut microflora for cattle, gut is very important because of the whole micro processes going on in the gut, and therefore eating the right kind of plants and changing the microflora can impact on what comes out you know at the end of the day.<br><br>P: But the environment also influences the gut microflora, so where communities where people raise chickens and cows in their houses. Like those bad bacteria tend to get in the way of healthy gut.<br><br>P: Right. Right.<br><br>P: So then that has an impact.<br><br>P: But our contention about using probiotics is, when your gut system gets affected because of some infection, you know, especially diarrhea or whatever, then you sort of recompose, you know.<br><br>P: Sometimes, but if you are constantly in this environment, where you are exposed to fecal pathogens, and you develop environmental enteric dysfunction, and your gut microbiome is not healthy and your intestine is not healthy, and then you are not absorbing nutrients that you eat.<br><br>P: Exactly. Sort of has a cascading effect on a whole range of other things.
	Day 2 workshop:<br><br>P: The whole problem is a lot of people are not sure what is happening. Everybody is looking at a sort of a curative mode of, you know, action, when you have a desperate problem. So I just give you an example, yea. Why we form this technical committee in order to address this issue of antibiotic use, simply because two things in agriculture are very important. One is from the food security angle, which you have

	addressed, because food security meaning that we worry about bacterial diseases okay. I can talk about [name of country] and also maybe the countries in the region. Rice has got a major problem with the bacterial diseases and the only way we can prevent them is by resistance development and that is a long drawn out process, and pathogens evolve. So you get screwed up, you know. So we tried all sorts of preventions for bacterials diseases or curative infection.<br><br>So food security is a big issue. The second issue is markets. Our horticultural products you know. We lost the papaya industry. We are losing the papaya industry. We lost the citrus industry, and we were very happy when Florida got the citrus greening problem, because we thought that would be some… [group laughed], happy in the sense that I am sorry, but not happy in the, not in the wrong sense, but happy in the sense that we know that there is going to be a lot of fundamental research going into it. America will support this. USA is very far ahead, but still they have not resolved the issue.<br><br>So in 1998 we had this what we call the [name of place] [inaudible]. They wanted to plant 500 hectares of this very sweet orange and it was a great export potential, but then this citrus greening came, and citrus greening is like HIV. Once you get it, it is very difficult to remove from the system, because it is a bacterial disease and if you could find a solution to a bacterial problem today in agriculture, it is a Holy Grail. You are a multi-millionaire. You make money.<br><br>So people come with all kinds of combinations, you know in terms of trying to use bactericides, you know antimicrobials. So what happened was people were, this citrus greening came to [name of country] in 2000… around 2004, 2005, and it sort of wiped out this [name of place]. Yea, sort of wiped out. It slowly sort of depleted the whole industry, and that is why we came and we got worried, and there were also a lot of products, agricultural products coming in which were like you say growth enhancers, you know people were modifying bacteria easier to modify by technology modify some of this, and transfer this bacteria and this was also a big concerns about what happens if there are conjugated strains, and you know all these hybridization between bacteria.<br><br>So it is from the soil, from people, the problem. There was one professor from [name of country] went there, very serious problem with the citrus greening. [Professor] actually came there and gave recommendations for our citrus farmers, especially this honey melon and also watermelon. Not watermelon, this pumelo, which is a big industry…to inject and that was a real problem. America doesn't do that. America sprays. They spray, so but it is a bit gray in terms of that, they started Injecting. So we got worried you know when you start injecting, because the reason for injecting is this particular bacteria affects the physiology system of a plant. And therefore, it blocks, it is like a heart problem you know where you have

	cholesterol in your heart. Your arteries get blocked, and therefore the whole plant slowly dies. So they started injecting.<br><br>P: Of course it worked for some plants, but it didn't work for a lot of things, and that concerned us, because of the physiological systems, you know going into the soil, the water system, 12lab la. So this is where the issue came, and until now we have not resolved the problem, although we have given recommendations how we could minimize the use of these antibiotics, but still you know the citrus industry is gone. So in that sense our export is gone basically.<br><br>R: That affects market.<br><br>P: So yea, that is a market factor.
	Day 2 workshop:<br><br>P: Everybody… you know farmers are desperate, the countries are desperate. Because my experience working with the pest control, yea, we find very little solutions for bacterial related problems. And so the only way is either you use a sort of a curative action, in fact going up to even using soap solution, you know, J-binds and whatever, plus antibiotics, but then now governments recognize that you know antibiotics that is not the good way to go, because of this resistance issue, and therefore they have sort of now coming out with these action plans, but what is the…what are the solutions that they are providing, you know to, to [inaudible] us for example. And so in Florida almost 90% of the trees are affected now, even though you know we thought America will come up with the solution, but now they are very worried that it will fly to you know, because it is spread by a vector, and so they are worried that it will go to the California citrus industry, and Australia is [berserk] you know, because Australia has got a very big citrus, so export market and when the industry is very, very crucial.<br><br>P: So from the food security angle, and also food security in the context of bacterial diseases affecting food security crops, like rice. It is a very serious problem. Yea, we don't have a bacterial-cide I would say. Similarly we don't have a virus-cide, [inaudible]-cide we don't have. We have a lot of fungicides, but not bactericides. The other thing is of course now extended use of soft approaches, chemicals like [inaudible] which is now coming into, it had been used for quite some time, but people are bringing transgenic species, [inaudible] canola in Canada. All these things are also causing problems in terms of genes floating over across systems.
	Day 2 workshop:<br><br>P: Yes. Yes for Southeast Asia countries it is a food security crop.<br><br>P: So like from my perspective as a nutritionist I consider like all the crops, food security crops.

	P: Sure. Sure. What I mean is ..<br><br>P: Like it is a major crop.<br><br>P: Yea, basically governments when they strategize, when they strategize crops as food security, they have a couple of crops. They usually use, of course here when we talk about food security, it is just not about the quantity. It is also the quality. So you are also talking about nutrient …<br><br>P: Nutrient densities<br><br>P: The nutrient hunger, sort of thing. But yea basically when Southeast Asian countries put food security as their number one issue, it is to rice. Number one is rice. Everything goes back to rice. If you don't talk about rice, don't talk to the government, yea? [another P laughed] Yea, because a huge amount, Malaysians spend about, we spend about almost a half a billion dollars on subsidies just to keep people growing rice. Half a billion.<br><br>P: But don't you think that is a problem, well like it just sounds like a problem nutrition wise, like I just think about [name of country] where 87% of people's calories come from rice, and so then they are severely nutrient deficient.<br><br>P: malnourished. Yea, yea.<br><br>P: So I think, yea I think, this is a cultural thing. So basically… you know when we had the… demonstrations. Food security became a big issue, you know [inaudible] and that kind of stuff. In 2008 there was a problem with rice production. The moment, because rice is very funny, because all of us are growing rice in Southeast Asia. People eat three times a day, rice. [P laughed] This is the thing. Although we don't as we grow older, we only take it for lunch, but other than that we take a lot of rice, but the problem is rice is traded, only 5% of world rice is traded, which means every country produces their own rice and they want this as security. Whatever happens? You know rice is not an economical crop. Put it this way. Meaning that your investment, rice, government invests money. Not for the sake of making money. They invest in rice because they need to have rice, because people need rice. So it is an uneconomical crop.<br><br>P: Yea, but I think there are not getting anywhere economically, and the countries are not getting anywhere nutritionally, because there is too much rice.<br><br>P: Exactly.<br><br>P: And not enough of the other.

	P: True. I think we understand that…I think people down the level you know they don't understand that, because for them without eating rice, at least two or three times a day, it is impossible. I know I go to [place in a non SEA country], and I am eating just salads and you know sandwiches. Where is my rice? You know. Once a day, so anyway they have to spend so much money in order to do that. So although it is now they are looking at it from the nutritional imbalance and so on. So rice is very important. We spend so much money… to produce rice. Yea.<br><br>R: So (name of participant) is there something we need to capture here with the nutrition, the food, the crop production…<br><br>P: Yea well I think we are kind of circling around the food security that it is difficult because we are sort of defining it differently. So I don't know if we want to say like, make another node, diet quality, and like I don't know how you would say that.<br><br>P: There is something here.<br><br>P: Like suitability and ….<br><br>P: [There is] something here on nutritional composition of diet.<br><br>R: Does that get it?<br><br>P: Better food security<br><br>P: But that could play into like crops, like crop characteristics. Sorry.<br><br>P: Because when you talk about food security, you are also talking about nutritional security. It is all built in.<br><br>P: Yea well in my mind food security, yea like food security and nutrition security. There is a lot of overlap.<br><br>P: Yea the quality/quantity.<br><br>P: Yea. So dietary quality is influenced by the food environment, which is influenced by what crops people grow, which is a factor of suitability. So I don't know if like there is a link between suitability and crop diversification.<br><br>R: Yea, and I'm also wondering if you are growing unsuitable food, it is not as nutrient dense.<br><br>P: Right, like the soil.

	R: So if they [grow food that is unsuitable], so would that go to nutrient composition?<br><br>P: Nutrient composition of the diet. Yea.<br><br>P: But we must also think that when you talk about nutrient, you know, quality, it is just not purely rice, because we do grow a lot of other crops, agricultural crops.<br><br>P: Right.<br><br>P: And vegetables, so there are also bacterial diseases affecting those crops. So that also impacts the farm you know. Like for example, tomatoes, you know somebody brought up about tomatoes, this we are bacterial virgin tomato. There is no solution, except to do grafting, but you know you can't use antibiotics you know for those kinds of things.<br><br>R: Can we tie, what I did was I put food environment, this kind of influences the crops that are selected, which influences nutritional composition of diet...<br><br>P: Yea, so I guess then the, if you have a varied nutritional composition of your diet, so you have rice and vegetables and different animal source foods than that is going to [place the] arrow [on the model] over to better diet quality. Yea.<br><br>R: But I think we also are missing the economic piece. So the government, because of food security or fear of insecurity, the government invests money into different crops. So would that be again to the suitability or …<br><br>P: Yea I think so. I think it all plays when the government is deciding the suitable crops, and they are deciding that they want to invest in rice and rice and rice, then I think …<br><br>P: I think they recognize. I think governments recognize this factor called hidden hunger that looks at micronutrients, you know the nutritional quality. So food security in the context of hidden hunger …it's also interesting.<br><br>P: Yea, but maybe it is not addressed for this.<br><br>P: The emphasis is not so much compared to rice.<br><br>P: Right. Right. Yea.<br><br>P: Rice is always on the top of the agenda.<br><br>P: Well it is an important cultural food.

	Day 2 workshop:<br>….<br>P: …. We need more micro-nutrients, and I do believe that monoculture is a drive to disease and …<br><br>P: And decrease micronutrients intake<br><br>P: That is the big problem. Commercial. Yea.<br><br>P: Because the farming regulations in the literature, they are probably having the system that we are not talking about them and this is what is important. This is a mission [of name of organization], right? To help these people to access those protein, or those micronutrients.

OVERARCHING FACTOR: Changing socioeconomic structure

**Changing socioeconomic structure:**<br><br>Poverty, affluence, lifestyle changes and consumer demand	Day 1 workshop:<br><br>R: …but it does link into consumption of other non-meat products consumption and there is another comment here - by changing structure, socioeconomic systems in Southeast Asia, there are more moving into the upper column and they have a great demand for some of these…<br><br>R: Socioeconomic status has increased …<br><br>P: Changing food behaviour actually.<br><br>R: Changing food behavior.<br><br>R: And consumption?<br><br>P: Yes.<br><br>P: And I think it might change nutritional composition of that. I think it is not just, I think it is not in this case, the socioeconomic status, but there is right now a change that is happening in the structure of the socioeconomic status. So it is not just the status itself, but there are more moving from ….<br><br>P: Abstractor.<br>…<br>P: about gut flora, just a small point, what we are eating probably also has an effect on the gut flora….So for example, in [name of country] they use a lot of curry using that much. So can change the dynamics in the gut flora has a direct influence on.
	Interview A:

	P: you know alternative protein sources and how that relates to food security, etc. but I haven't you know thought it through fully in relation to AMR, but it is hard to know whether it will, and how things can change, but you know I guess as the affluence of the region increases and to some degree it is probably more inequality, but that means you know people may be able to pay more for food and for meat products, and some of the externalities that are currently not included in the price of meat and farm animal products, might be able to be better incorporated to also take some pressure off you know the profit making and conventional production systems. So in some ways having that all translate through to the consumer, I could see could help facilitate and this is sort of you know we work with the retailer, producer and consumer sort of level, it might help to facilitate producers to increase their [animal] welfare and take some pressure off the system. So there are indirect influences that they could influence the whole system.
	Day 2 workshop<br><br>P: Our consumer behaviour lifestyle change and we are willing to pay this convenience frozen food and so on. So the producer, put more things out there. I mean look at our dietary requirement. We, most of us has surpassed our dietary requirement, you know. We don't eat to live anymore. We live to eat. You know, we are twenty-four hour convenience store. In [name of country] we are twenty-four hour MacDonald, KFC…Yes. So we are changing. When you do that, open more time to eat more likely to eat, you cause food waste. Food waste has impact in everything. Resources, oil, land, labour, and it costs more for antibiotics to get the whole system up for people to choose, you know we check objects based on size, colour, shape, you know. Banana needs to be C shape, you know. A straight banana is not a banana. It comes to that point, alright?. Apple with some blandish, even the case, everything is the same. We recheck, even the hypermarket is salted up cosmetically, esthetically and we still reject, right. So it creates all this, you know.<br>….<br><br>Like in old days we hunt for the food we eat. You know just hunt for your family. Now here we are hunt for [name of country] family and export you know.<br><br>P: Economics are naïve. [inaudible]. Profitability.<br><br>P: I think the key thing to sort of point, is what are the factors that drive antimicrobial resistance. There are major driving forces, you know. Pull and push factors, yeah? , markets, security, food, affluence, you know, changes in lifestyles…
	Day 2 workshop:<br><br>P: I am looking at my perspective as educating food waste in this country, like what I know is we actually are practicing a very rotten food system

	locally, you know. And I think starting all are quite man made from people like us. Like what the (name of participant) was mentioning. We demand perfect food, and want it cheap and so on. And on the lifestyle, the fast food, the convenience store frozen food and all these things we have created convenience is all come from this people, and the population is growing. I mean whatever you are trying to do is fine, but the way I look at it, it is just one small portion of what is here on the production of food, right? I look at it from my perspective as a layman when I sit down here, is another shocking thing for me, that I can put into my [inaudible] whatever I meet in the future, you know. What I know is it is all created from evolution and so on, the lifestyle, the population, and that creates, things become very commercial. We need to produce at the speed and everything commercialized, packaging material and so forth, and we abandoned organic farming you know.<br>In that sense, that to speed up, because our rejection of imperfect food we need to, farmer has got to plant more to sort of offset the rejection. So from the farmer, there is a lot of losses and so forth. It is all in the name of profitability. This is what is happening, and with this, when it comes to production, to do it we need to administer like more antibiotics, herbicide, pesticide, whatever, so that you can get a better yield, you know, and this component is basically here what we are looking at. Of course I am not so technical how to contribute, but I think while we are doing this and me doing the food waste, we need to educate you know in school in my eleven years in secondary or primary school, nothing was taught on all these shocking, you know. We learn to as we move on, but we can inject education everywhere, even from small, you know something that people know and learn to respect food. How food comes about. How much resources has been wasted. You know how much of this thing affect our health.<br><br>This is like, it is all known even Americans have now been running a 42% of American are having cancer by the age of seventy-two you know. Fifty years ago cancer is unheard of you know. In my country [name of country], it is 25%, and as the country developed, because you demand [inaudible], percentages will go up, so I think education is important for us to do that. And the food wasting issue, because of our demand lifestyle, what would produce and we know from the people here obesity, five hundred million around the world. Even in my country, [name of country] we are among the factors in this region, compared to… [name of three other SEA countries] and so it is happening. We are basically copying the American model, as we progress [economically], we get larger and so on, I don't think taller. [laughter] So I think we need to inject education everywhere into people.
	Interview A:<br><br>and then also we have touch points with the consumers, public, and other related things to, in relation to diet and livestock products and non-communicable diseases, and that sort of thing,

**Changing socioeconomic structures:**<br><br>Labour changes	Day 2 workshop:<br><br>R: One thing that I am kind of picking up from that is the economics was driving the job market and so the job market is not profitable for farmers, so that is a driver that we don't really have.<br><br>P: Big issue because we have got a big theme on youth in agriculture in every country that we work. You talk about, [name of country[, even [name of another country]… with a large population, nobody wants to do agriculture, and so I got a surprise when they started asking us about you now dehusking machines on coconuts, because we thought [name of country] will have enough people to do dehusking. They said, can you give us your machine, nobody wants to dehusk. So nobody wants to [do] agriculture. So in a way we have to face up to this reality that sooner or later things are going to be very intensive. You have to make sure you are prepared for it…<br><br>P: Especially more now than doctors.<br><br>P: Yea. Yea. No it is true.<br><br>P: Our business to keep, our business. I just over the weekend, I asked my [name of country] worker, to come and clean my garden. You know my wife was complaining, yea before coming here. No before coming to [name of country], I pay him fifty ringgit for almost forty-five minutes work. [Now] I pay the same guy, I pay two hundred ringgit for two hours work…So I totally his salary is more than mine you know. So this is hard work. No. No. Serious. I mean I pay 30 ringgit, 40 ringgit for a day's work. Our workers get about sixty ringgit per day's work. This guy got forty-five minutes, he gets fifty ringgit to clean my little patch of garden you know. He says you either do it or you pay good. So labour is a very big issue. Anyway. [Group laughed]
	Day 2 workshop:<br><br>P:…sooner or later, as the population increases, fewer people are going to produce food for more people. It is inevitable. Yea, because you find people are not going into agriculture. A lot of them are you know everybody wants to go into IT and do other things. Everyone wants to eat food, but nobody wants to produce, because it is hard work. It is really hard work. It is not easy to do, agriculture, which means sooner or later you are going to this very large industrial scale systems, and so it becomes very inevitable you know and so one has to be very careful about whether you find alternatives through research and so on. To sort of feed those systems, you know, rather than ….
	Day 2 workshop:<br><br>P: I think the key thing to sort of point, is what are the factors that drive antimicrobial resistance. There are major driving forces, you know. Pull

	and push factors, yeah? markets, security, food, affluence, you know, changes in lifestyles…

OVERARCHING FACTOR: Governance, regulations and enforcement

**Governance, regulations and enforcement:**<br><br>Governance mechanisms (e.g., international, regional, domestic, industry policies, voluntary market/product certifications, knowledge brokering/technical assistance, and associated benefits and challenges).	Day 1 workshop:<br><br>Reading an organization's policies around AMU:<br><br>P: Reduce harm (from antimicrobial use)<br><br>R: Is this just in [name of country] or all of Southeast Asia?<br><br>P: Yea.<br><br>R: Is it through the whole production line of Southeast Asia?<br><br>P: Yes. Yes. Pig, chicken, and shrimp. All of them.<br>…<br>R: So I might start with the general, should we start here? Okay, so we have here [P's name] has provided us with the global vision for antimicrobial use stewardship and food animals and from [name of organization] and their mission is producing safe, high quality and sustainable food with responsible use of antimicrobials and so their key priority per, how, what they are trying to do in terms of influencing policy in antimicrobial use stewardship, one is enacting global best practices for responsible antimicrobial use.<br><br>[name of organization] prioritizes those antimicrobials that are medically important for human medicine to be used only for therapeutic uses under veterinary oversight, and that antimicrobials that are animal only, or those that are not medically important for human medicine, shall be considered as the first option for use. So it is AMR stewardship and veterinary sort of piece. So I am wondering here, it sounds like there is, so is the vet in Southeast Asia, is it just in [name of a SEA country], it sounds like you need a prescription from a veterinary doctor to get medications for animals. Is that all across Southeast Asia? Or…<br><br>P: In the fish farm. The law not recommend for veterinary to recommend that.<br><br>R: Okay.<br><br>P: Yea, [name of org] to [sign] originally off farm fish and farm shrimp, when you use antibiotic, [inaudible] to authorize in [name of country, name of another country, name of another country]….<br><br>R: They all need veterinary prescriptions. Okay.<br><br>P: Yes.

	R: This is about AMR stewardship. I am going to mark it on the model, it is medically important for human use. So these influences is what is going on at the farms. From use, I am assuming would have a decreased use on farms of medically important ones, and then the other one is elimination of the use of shared class antimicrobials which are medically important for human medicine for growth promotion purposes globally. So, this is also here, goes to growth promotion I am assuming?<br><br>P: That is correct.<br><br>R: Growth promotion. There it is. Yea. So that will reduce, right?<br><br>P: Yea.<br><br>R: And then working with global experts and partners with all stakeholders to identify new and better ways to care for the animals, to enhance animal welfare and reduce the need for antimicrobials. So this idea of collaboration across different sectors, experts and partners with all stakeholders. So what kind of stakeholders do you engage? Is it, who would you have at the table to try and collaborate to do this one? Increase, work with global experts and partners, so it is researchers, it is people in the industry, is it people with …<br><br>P: All, for my partner.<br><br>R: Stakeholders<br><br>P: My farm… to buy my feed.<br><br>P: And I had veterinary some, veterinary to take care…to my customers.<br><br>R: Okay. So it is with the farmers and the customers that you buy the feed from. I think that is what I am understanding. So you have got collaboration will influence AM stewardship.<br><br>Okay, and then develop internal monitoring. So we have surveillance as well to, so develop antimicrobial resistance monitoring through collaboration with national, international government organizations to advance the antimicrobial stewardship program. So, part of this that is surveillance.<br><br>P: Just a question, [R: hm-mm?] Because in the model it says terrestrials on farms, so that means they only focus on land animals.<br><br>R: …We have agriculture on farm use here.<br><br>P: Oh I see.

	R: And we have up here for crop, like non-antimicrobial use in non-animal agriculture, so horticultural crops, ethanol production kind of stuff, and we have antimicrobial use in wildlife, and human antimicrobial use here.<br><br>R: Yes. Is there anyone here with agriculture, we are going to tie that, this part to our stewardship, this stuff.<br><br>R: So it would go right from the, from here…Does that capture what you were saying?<br><br>P: Yea, because I think it is the only case between those in aquaculture.<br>…<br>R: So you think also like, so we have terrestrial farm use, we have aquaculture use, but this isn't tied to animal welfare and it is not tied to aquatic illness.<br><br>P: Yes, because for us it is the same, even aqua or land animals, we need to prepare security, good animal welfare<br><br>P: with animal welfare.<br><br>R: In the aquatics sector.<br><br>P: Yes, and more fair for aquatic animal too.<br>…<br>P: But. Another question, in Canada, a veterinarian responsible for both aqua and land animals?<br>…<br>R: We have some specifics that are just for aquaculture, but yes, they can be responsible…<br><br>P: Because in Thailand I think land animals are managed by livestock departments but aqua managed by [another P: fishery] fishery organizations.<br><br>R: By fisheries?<br><br>P: Yes, and those organizations, for example, as my understanding in [name of a SEA country]. The fishery part is [another P: not recommended] not recommended veterinarians involved so, I mean people just can put whatever they want in there.<br><br>P: Yea, farmer to use.<br><br>P: I am not sure it is the same.<br><br>P: …in many of southeast Asian countries, common authority for reporting to is the chief [executive] officer. But in many southeast Asian

	Countries, the veterinary divisions do not have much role and the interest [inaudible] in aquatics. So OIE as it stands, global standards, has given multiple aquatic focal points, it is the responsibility of the chief [inaudible] officer of each of these countries, to appoint a OIE aquatic focal point, and [name of a SEA country] is leading in appointing the [inaudible] aquatic focal points. Only some countries help take up the initiative based on the OIE recommendation, and they have employed, they have nominated OIE aquatic focal points. In [name of a SEA country] [inaudible] aquatic [inaudible] institution is the OIE aquatic focal point and he or she is responsible to report to the chief [executive] officer. So while all certification, everything is done through the common country mechanism, and the Department of Fisheries has the full legal rights, under the OIE, and the local CVO to do that. Prescription, non-prescription, whatever, punishments, whatever, but that's – it's not as it's fully regulated. It is not half-hazard, it's full regulated… in many countries.<br><br>R: In many countries it is like that?<br><br>P: In many countries…<br><br>R: Across Southeast Asia?<br><br>P: Yes, which has appointed for OIE aquatic focal point under the CBO of a particular country. What does that mean? Some countries unfortunately, I am very sorry to say that, unfortunately the reps do not want to give the delegation and the power to the local fish people and they are holding on to it, and they are going to the OIE assemblies and not really reporting any [inaudible] on the fisheries and giving it a bad impression about the fish. So it is the responsibility of the OIE rep authorities in these countries to delegate and seek the information from the respective [fish] department. You just take the OIE list now and can see OIE focal points are [inaudible] from more than 20 focal points, [inaudible] from more than 21 to 25 countries. Some countries the [inaudible] departments are [inaudible] proactive and very responsible to you and asking you follow all this, you with me. But some countries only the OIE delegate wants to go to OIE assembly. He doesn't go to the fish… company, so he is not appointed. So it is the mistake of the recommended authority of the country. [R: Okay] OIE has given the powers as a global standard agreed in one of the world general assembles passed by all the 191 members…You can just see the OIE documents, just go to the OIE [Inaudible]… you see all the respected authorities. The only thing the letter goes through the common authority.<br>…<br>P: It's some of the southeast Asian countries…not all. I know in [name of country], I know in [name of another SEA country], I know [name of a SEA country], [name of a different SEA country], [name of a different SEA country], [name of another SEA country], and [name of another SEA country], [name of another SEA country], there are a lot of OIE focal

	points appointed by the national [corporate?] authorities, which is the CVO, chief [inaudible] officer. [Sadly], it's not optimal because…<br><br>R: So how does that kind of, does it… so we have got to going to influences use in antimicrobials in aquaculture. This regulation [P: yea, this regulation applies, yes] and how this is set up. Is there anything else that we should, that influences here?<br><br>P: We could [inaudible] aquatic animal resistance surveillance, national reporting and international reporting. All the health certification, everything goes through the OIE [inaudible] focal point.<br><br>P: Including your… antibiotics certification has to go. It goes through focal points. That means the Department of Ministries and more, fisheries, Ministries and Department of [inaudible]in giving this [inaudible].
	Day 1 workshop:<br><br>P: [inaudible] Because, I mean, [OIE] have set up standards that is important in supporting reductions in antimicrobial use here, supporting antimicrobials. I show the example [inaudible] reducing usage of antimicrobials in animals. I think it is directly relates to all these things, because once you do the proper [inaudible] … and you do the reporting, and sharing of the information and if you're strong enough to [implement] international standards, services, so it will cut down, among government [inaudible]so I don't know how.<br><br>R: It also affected domestic standards, it also affects veterinary service<br><br>[P's comments were inaudible]<br>…<br>P: Part of the international standard.<br><br>R: So is that part of the international standard as a direct link?<br><br>P: Yes.<br><br>R: We think the international standards directly leads to good farming practices or through domestic standards?<br><br>P: Both. Yea. Because most of the domestic standards are drawn from the international standard and also there is some difference to [inaudible].
	Day 1 workshop:<br><br>P: I would like to mention the 8 focal points OIE introduced for each country to facilitate e the process of implementation of OIE standards as follows: animal disease notification, animal welfare, aquatic animal, communication, animal production food safety, laboratories, veterinary products, wildlife. The focal points are usually the experts in the particular

	sector in the country and they are to support the OIE delegate to prepare comments for draft standards and also to provide expert assistance to implement standards at country level, mainly through embedding them to local laws and regulation. In the meantime, to appoint focal points by the OIE Delegates, there is no set procedure or a protocol at country level. It completely depending on the decision of the delegate. In some cases, such as wildlife and aquatic, the focal point can be outside the veterinary service as usually aquaculture and wildlife are under separate ministries or organizations. Otherwise, usually all the focal points are under the veterinary service.
	Day 1 workshop:<br><br>P: Basically, it involves international standards and everything into one. It is not easy.<br><br>R: Yea, so challenge … it's integrating international and other regulations. Is that what you mean, international standards?<br><br>P: Yea, I mean… actually I work in the [name of organization], these [inaudible] international standards include public rules.<br><br>R: Yea, that is a challenge. Integrate international standards. [name of organization] examples into local regulations. Challenge to making this happen.<br><br>P: Actually I think is the farmers, the farmer sector, the private sector together, it is not difficult to implement those things, they can [inaudible], it is quite easy to implement but they have conflict of interest.<br><br>R: So the government sector, farmers.<br><br>P: And industry.
	Interview A:<br><br>P: It is interesting to note …the OIE veterinary list of antibiotics is almost an inversion of the WHO CIA list – yet transference of resistance is clearly shown via plasmids, DNA etc and even to probiotics, some disinfectants.
	Day 1 workshop:<br><br>R: So the next point is there is product certifications. So what is being said is product certification is …we apply different hygiene practices, but also food safety. So is that international standard still?<br><br>P: Some are market difference [inaudible] partly countries international trade.<br><br>P: So it has to be [inaudible] or something has to be certified.

	P: They all link to hygiene practice and …<br><br>R: Linked to good hygiene practice on the farm.<br><br>R: But also trade and ….<br><br>R: And…is it linked to using less antibiotics?<br><br>P: No. No…No, what I mean it is like now there're a lot of market driven and voluntary certification program to certify the production process. So to be selling under that brand, it's must have [inaudible] around use and the [anti]bacterials, food security, good hygiene practices, …so in one way if all fish produced wants to be certified, through market-driven [voluntary] international, then the whole issue has significantly come down, but now less than one percent of the fish that is produced is certified.<br><br>P: That certificate is different from the OIE international trade certificate, so there's always the impression that people go to the supermarkets, see how they are certified. Who is certified? An independent [inaudible] system is certified. Countries want to be certified. It is a niche market certification. [inaudible] certification, land and soil certification. So those certifications are distinguished from the clear [international] standards.<br>…<br>P: It is consumer driven also, it is a consumer driven market driven you know. Third party, [R: we don't have it], you don't have it, but it is a big contributor. They are all certified for example. Consumers only buy whatever organic poultry or whatever. Then it is happening.
	Day 1 workshop:<br><br>P: There are a lot of production process certification at each level. It is responding to the market. GAS certification is a big thing for aquaculture, global aquaculture [and land] certification. They certified hundreds of farms in [name of a SEA country], [name of a different SEA country], [name of another SEA country]. So the market is happy to accept that. So then one of the components will be responsible use and less use or no use of chemicals in farm practices.<br><br>R: so it's global and domestic product certification?<br><br>P: yea, certification, yea, just two.
	Day 2 workshop:<br><br>P: Yes okay. So coming from food manufacturing background and also I am going to talk about food safety right. I am going to talk about more on food safety education. So I don't really see that the points are already there, so okay. So the type of food certification that is different than what I say is that… to the food industry, right and there is a big emphasis on

	food taking raw materials from vendors that are [inaudible], who has a good track record of you know producing, and selling meat which are not tainted so in [name of country] we have seen that kind of trend growing. So we hear things like ABF - antimicrobial-free chicken - so it is on the rise, that kind of thing, then why so, it is because we see the Japanese companies coming here to [name of country] companies especially in poultry, right, in preparation for the next Olympics in Tokyo, right. So some of them come here to produce Halal poultry. Right, so when they came here, they also have [inaudible] So what we see is that now that they have only, they can pick their raw materials, the meat that they process from vendors, which has high or good track records. So what I am saying is that an increase in food safety education, on certification can actually lead to you know, can influence.<br><br>R: So I have got quality assurance…certification of food…<br><br>P: Right. It is better to put maybe food safety certification.<br>…<br>R: … there is this factor of international mergers. International trade.<br><br>P: Yes.<br><br>R: And they want these certifications.<br><br>P: Yes. Right.<br><br>R: Which is driven by business.<br><br>P: Yea. International demand yea.<br><br>R: Yea. So it is trade, but also these companies.<br><br>P: Right.<br><br>P: Trade plus companies, especially those that bring to the GFSI. They want this kind of conditions.<br><br>R: Yea, and say like when the users or something comes in, they want to buy only antibiotic free so it is international retailers.
	Interview A:<br><br>P: I think, I guess so. I think that is sort of small… and unregulated, pretty much unregulated trade, and I mentioned, I attached a little bit on either "regulated trade" or trade within vertically integrated systems. So as you would have probably gleaned, the country is very hugely in the role and predominance of vertically integrated companies. So [name of a SEA country] probably trumps that and then [names of three other SEA countries], but they would be, I guess though, vertically integrated

	companies have more control to some degree over antibiotic use generally, but they have contract farmers and usually they specify and control the drugs they have, but we have also found that you know there are things that go beyond those bounds as well. So there is an aspect of industry compliance and long compliance that perhaps is another node, and sort of underneath if you like, the level of national, international agreements and standards, etc. etc.<br><br>R: Okay, and by integrated, trade vertically integrated systems…if you can just explain that for me.<br><br>P: Yea sure. So I guess for example, in [name of country], 89% of [type of animal] and [type of animal] production is by vertically integrated companies, dominated by six or seven companies…and they will have their own farms. They will have contract farms. They will have their own sort of houses, and processing factories, and then there will be others that go to other slaughterhouses and processing factories usually some of the contract farms, and they may have their own retail outlets or they may certainly supply B2B business or to wet markets, or to large supermarkets, etc., or they have as I say, their own retail outlets.<br><br>P: Now they will aim to control and they have a [an agreement] with the government to restrict the use of certain antibiotics and they have an interest in doing that, and some of them have even gone to certification, of raised without antibiotics through the NFS or NFA certification in America. So they will have an interest in trying to reduce antibiotic and antimicrobial use and that is following an expectation to their contract farms as well. However, there will still be endemic disease on those farms. There will be endemic PRRS, porcine respiratory and reproduction syndrome, which works in a triangle of bacterial and management factors, to have clinical disease or not.
	Day 2 workshop:<br><br>P: Basically we have got ASEAN network, collect network, you know, under the ASEAN secretariat ….I think usually all the countries share this information under the ASEAN agriculture…so a couple of years back, when… couple of years back, you know, [plant clinic] don't encourage people using antibiotics you know when…give recommendations…. [R: what clinics?] Plant clinics, just like human clinics and you know farmers in many countries recommended antibiotics, because the major problem with antibiotics is you know controlling bacterial diseases, it's a… how is it…it is the Holy Grail. It is a major impediment for crops, yea? Particularly rice. So farmers tend to resort to you know desperate measures I would say.<br><br>So they use antibiotics basically, so but [plant clinics] don't encourage that because of all these issues, and I think that…

	Nobody uses antibiotics now. It is not officially registered. Previously they used to register, erythromycin, streptomycin, [another antibiotic inaudible], base products. But now they say, no it is not officially registered, which means coming back to the question of harmonization between countries. Usually they share a lot of these things among all the countries.<br>…<br>P: …at least they got some regulatory framework or they build this framework and I am sure some of this action plans come as a result of this meeting that they have that we have assembled.<br>…<br>A very comprehensive document, but I am not sure the operational plans, whether they have overall framework action plan that says oh you should be very careful about how you use your antimicrobial.<br>…<br>[Plant clinic] go by good agriculture practice standards so…encourage [farmers] not to use, although there are a lot of desperation.
	Day 1 workshop:<br><br>P: Technical services from private sector technical representative do not reach small farmers, while service [officers] from the government are also limited.
	Day 2 workshop:<br><br>P: Because there is a big overlap… in the… [R: in the crops too?]<br><br>R: The crops, because there is often not crop health professional.<br><br>P: Right.
**Governance, regulations and enforcement:**<br><br>Regulations on the design of antibiotics for veterinary use	Day 2 workshop:<br><br>P: So basically most of the antibiotics that we talk about, most of them are poorly soluble water for example. So for the product to be effective, first of all the product must get dissolved, [pause] must dissolve. Maybe in water, or in the GIT. [P: gastro-intestinal track?] Gastro-intestinal track. So for it to be effective it also should get absorbed and then it should achieve the particular level of concentration in the body fluid or in the blood for example. So if the product that you are administering, we are not sure whether you know could be you know a product which has been developed for humans. So whether this product has been …modified to be used in livestock for example, or that it has been used for using in aquaculture. So that is also important and then antibiotic that you are using, although we don't know what is this antibiotics, whether those antibiotics are being used as a base, or it can be used as a [inaudible - solvent?] which is water soluble. So very important. That is going to decide whether the product is going to be dissolved, absorbed to get to the particular level of concentration.

	So therefore product design is important. Product design and product selection. [pause] So in the Southeast Asia context, maybe we are not sure whether we have a proper advisory in terms of you know what is a suitable product or drug and whether it is in the proper form to be used and how it should be administered. What should be the dose, for example? [pause] So these are the criteria's that are to be considered in order to be effective in terms of the efficacy of the antibiotic. Say if you are not considering these aspects it is a possibility that the product will be something or not. Probably you will have a particular level of concentration of the drug, resulting in you know the resistance basically. You have resistance to the antibiotics, then you might end up having resistance strains of microbes and infections. So this is very important aspect here is to select a product which can be, you know, which can be used and proper advisory and also in terms of regulatory framework, you know, whether the proper regulatory framework is there you know, to control the use of drugs and to use them properly like for the specific purpose.<br><br>R: Okay and does that exist?<br><br>P: No, it does not really. Not, I mean I am not very sure. Not very sure of the regulatory, you know, framework.<br><br>R: Now the regulatory framework to control the antibiotics are properly, I missed your words that you said, that are properly …<br><br>P: Not properly used for the specific purpose…And then because sometimes the decision is made by probably the farm, you know, the [farm] owners.<br>…<br>P: And probably some kind of guidance, from the veterinary, you know, sort of doctors or somebody. Who may not be having a full understanding of, you know, a complete understanding of the product. For example, if your product is not very soluble, then you might have a lot of residues floating on the water, I think there's some, you know, publications, where they are told that the resistance is mainly because the drug which is floating is undissolved. It has been consumed by the fish and you know this kind of thing. Then probably, that might also lead to, you know, resistance for the drug.<br><br>R: So it applies also to the aquaculture side.<br><br>P: Yea…
	Day 2 workshop:<br><br>P: I want to add something to that, that is very true, there is not much control in animals, but there is a national pharmaceutical regulatory [inaudible] organization which actually ….

	P: Yea, they regulate, they regulate. They are licensed also, there is a license activity actually, in [name of country], it is licensed, [P: but not much in humans], but in terms of it is not so strict as… as, for example, we need to prove a lot of things when you register a product of human use, but the dossier that we need to submit for the registering of a veterinary product is not as detailed as compared to submitting dossier for human use. Relatively LESS (emphasized). I am not saying no. Relatively less control. I think it should be, you know, it should not say that it is not controlled, but it is less controlled, relatively less controlled.<br><br>P: I think many of this have been addressed in the [name of country] Action Plan on Antimicrobial Resistance. This is the plan from 2017 to 2021. It is already published. Many of these issues have been addressed.<br>..<br>P: Yea. There is such a, I think all these things came out in a sort of converged, based on all these issues that were addressed, but I think the points raised by the animal industry is quite true, because we don't go very detailed into efficacy and so on. We just sort of adopt and adapt, you know. [laughed] Basically…
	Day 2 workshop:<br><br>P: The last point I would like to make is with respect to production of products, which normally there is a very stringent control and production of, you know, products for humans, but the regulation is less restricted for animals and fisheries. There is not much of controls on this kind of thing…<br>Relatively less you know in terms of controls and manufacturing and access… access as well as manufacturing you know, procedures, designs, you know in terms of product design, etc. There is also less control.<br><br>R: For facilities producing animal pharmaceuticals.<br><br>P: Yea.<br><br>R: So how would we link that? So the human side is strong.<br><br>P: Yea.<br><br>R: Antibiotics is one thing and then from there is like the regulation of it. On the human side what it would lead to. On the animal side there is less regulation.<br>And then human regulation of antibiotic manufacturing, like what I am hearing you say…on the human side it is strong, and on the animal side it is not.<br><br>P: Manufacturing sector facilities and you know control of it.

	R: It is kind of like quality assurance of the manufacturing.<br><br>R: And then that would influence how it is used, which would help decrease to control it.<br><br>P: So there is one more point here, in more point in terms of efficacy, in terms of efficacy, for example for humans, we need to prove that it is viable, that viability, it has to be proven. So a lot of data has to be submitted, but whereas in veterinary, I think that is not so much of control in terms of performance of the products and efficacy.<br><br>R: Right. Yea, if we had more efficacious products, it would decrease resistance.<br><br>P: Yea, the product is less efficacious, it will have more chances of resistance.<br><br>R: Resistance. Yea.<br><br>R: Yea, those are all great points.<br><br>R: So this one is stronger. So we need efficacy… human is strong… [writing on board]<br><br>P: Thank you
**Governance, regulations and enforcement:**<br><br>Regulations and surveillance on AMU, including different types of AMU (e.g., growth promotion, metaphylaxis, prevention)	Day 2 workshop:<br><br>P: And third point is that you know like especially in the case of preventing, we are talking about, so whether we should treat the, you know, the reason for better hygiene and other things so I would suggest in my opinion that you know using antibiotics as a preventive care, I think is a bit dangerous actually. So preventive care probably we should use more of improved hygiene, water quality or with immunobiotics, etc., [R: on farm] and then if at all you want to use preventive, vaccination is the best option, but if you are using antibiotic as a preventive, you know, then …<br>P: Are there any regulations against that in Southeast Asia that you know?<br><br>P: Not really. No.<br><br>P: So people can just do it, and they are going to do it if it is going to make it more profitable.<br><br>P: I mean yea this is the, not here I don't think there are restrictions on, you know, use of veterinary products, drugs. A lot of control is not there. I think there is not much of regulatory control.<br><br>R: And actually that is true also for high income countries.

	P: Yea.<br><br>R: The restrictions on preventive use are very, very rare. [P: oh really?] Restrictions on growth promotion is more common, but on prevention use they are trying, the European Union is starting to talk about having that for a certain drug classes, but it is very…We know, our poultry industry now has restrictions on certain categories for preventive use, but it is unusual. None of our other species have that. So yea. So I think, so you captured that preventive use that we would want to, so if we decrease preventive use, we are going to have decreased use overall and decreased resistance in the environment.
	Interview A:<br><br>R: … and you mentioned metaphylaxis…Is metaphylaxis its own node?<br><br>P: Yes, I think so…So perhaps you know having two that, the prevention of you know clinical and confirmed infections and useful metaphylaxis, and we know that that is happening in a wide scale in many industries.<br><br>R: And is metaphylaxis…. are the regulations around, like what is the sort of rule generally?<br><br>P: Yea, I know [name of SEA country] does, but I can also talk a little bit to [the names of 3 other SEA countries]. At this stage as you probably know, there are attempts or you know use or in some countries regulations around metaphylaxis in relation to antibiotics I might add. Not everything, but antibiotics used in feed and water. So in [name of SEA country] that exists, so they can be used under veterinary prescription, but it may not exist for every country. It does in theory exist I believe in [name of another SEA country], but it is still used. So there is a lot of undercurrents you know, illegal use also, because either it is broadly done under a veterinary provision, or it is just done illegally with mass use, and I know that because I was speaking to a [person from name of a SEA country] vet just the other day. So because it is not necessarily good enforcement in many of these markets, but the other area is – now in terms of general metaphylaxis, there is beyond feed and water use, which is usually the routes to metaphylaxis obviously, but there is not necessarily and the metaphylaxis and the feed and water use is usually restricted to antibiotics. It is not necessarily for anti-toxicity stats, or those antibiotics that are not considered critically important. So …other types of antibiotics, tetracycline obviously, amoxicillin and others.<br><br>So I think the enforcement and the regulation is generally lax and the observance of it, and then it is mostly focused on from a global push on growth promotion, not this metaphylaxis, and that is where I think there is a real, there is still a tension and that came up at the global [name of event] second meeting last year, a year ago, and also when I went to [name of another event], which is a large industry event for Southeast

	Asia, that tension still exists between I think certainly Europe and US, but also probably in the middle of that of course Southeast Asia and other low and middle income countries. So yes that is still a significant area that is a gap I would say.
	Interview A:<br><br>P: And it is a big, still a big, large remaining use of antimicrobials into the system, and so for example in [name of a SEA country] they can't use in theory legally certain antibiotics in feed or water, and unless prescribed for a vet. Now that would mean that they can't, okay just use them routinely off the [inaudible - growth?] promotion. However, if the vet agrees that there is a high risk perhaps of a certain disease, whether it be viral or bacterial, then they might prescribe that it can be used to prevent sort of a rise of say a bacterial related you know respiratory disease or gastrointestinal disease for example, and so I think splitting those two out is quite important because there is a lot of nuances around on the farm use in the system.
	Interview A:<br><br>P: the lack of transparency and reporting on AMU for growth promotion, mass prophylaxis vs treatment and related in part is the informal access plus lack of decoupling or other strategies between vet [or] company prescription and sales.
	Interview A:<br><br>P: Similarly, there is a lack of farming AMU targets – as in some countries in Europe – where targets are set per species [or] industry for average daily use (per animal)
	Day 2 workshop:<br>…<br>R: And one other comment about that preventive use, one of the ways of making it, and proving the stewardship is if things are through the veterinarian, so then the veterinarian can make a decision on whether the prevention is needed as opposed to direct access by the farmers purchasing it.<br><br>P: Yea.<br><br>R: So there is some …<br><br>P: That is a fact piece, I think we just established over a period of time, so you want for prevention, you can use antibiotics. So probably to change that [R: thinking] thinking, probably it is going to be a challenge. Yea.

	Interview A:<br><br>P: Regulatory factors…access, reporting sales and use– with agricultural and Pharma industries (plants / animals) is apparently missing [in this model]– this is obviously key.
**Governance, regulations and enforcement:**<br><br>Regulations on what antibiotics to use for agriculture (crops)	Day 2 workshop:<br><br>P: We have something here that, I don't know, tell me if I am saying it right, but the government of [name of country] three years ago.<br><br>P: A few years ago, stopped registering antibiotics, meaning it cut off farmers from accessing them.<br><br>P: From companies selling them or registering them, because the …[once you] registered them, you can use them legally. So in 1974 when the [inaudible] act came into being, there were about fifteen to sixteen different types of antibiotics used against bacterial diseases and also fungicides, because some of them do have some for anti-fungal you know properties, but recently after that review, which we did, and subsequently I think they put it into an act, and now if you go into the web site, registered pesticide web site, there is nothing. It is all removed totally. The same thing with [name of another SEA country]. [Name of country three years ago, three years ago they were using, you know antibiotics. It is quite common for Southeast Asian countries to use antibiotics, because it is easily available in retail shops.<br><br>R: But now you are saying it is not.<br><br>P: Now, no. Government doesn't officially have a list of antibiotics that can be used in agriculture.<br><br>R: So does that mean people aren't selling them anymore? Or is it like a black market thing?<br><br>P: It is a black market thing. It is not legal yea. It is not a registered list, because a registered list you can use, yea.<br>…<br>It doesn't matter even, pesticides go into you know black market you know. So a lot of unregistered pesticides are also being sold, but that is all cross border trade and that kind of thing.<br><br>P: But yea, but that is like a big barrier in [anti]microbial use.<br><br>P: But it is very sensitive to talk to a farmer you know. Governments may not like it, because they think that they are doing their job, but I think we know on the ground that it is not working.

	Day 2 workshop:<br><br>P: I would think that you don't legalize it by registering it. That is the government's point of view. We don't want to, it is like drugs. You don't legalize it, but of course the black market is always there. That is up to you, but as far as the government is concerned, because governments are party to international conventions you know. They are part of the WHO.<br><br>P: It is more like a shell.<br><br>P: So when you are in a government office, when you go for the international meetings, you have to put your very official standing on this. What have you done for this? So I would think that when the government goes in, and sits in this WHO meeting, they have to make sure, hey what are you doing about this intervention.
**Governance, regulations and enforcement:**<br><br>Regulations on who can prescribe antimicrobials, the role of perverse incentives and government price caps	Day 1 workshop:<br><br>P: Yes, exactly. And that is one of the other things I got on too, who is doing the prescribing and where are they prescribing. Is it doctors?...Is it doctors who prescribe? Is it nurses who are allowed to prescribe? Is that under medical control? In the UK nurses can't prescribe, but they are effectively doing it under, with permission of the doctor, who is giving advance permissions to do so or do they have autonomy to prescribe. Right.<br>…<br>R: So increased regulations will give us then better antimicrobial use.<br><br>P: Use. Yes. I'd expect that.<br><br>R: And then the other one is prescribing behavior?<br><br>P: Or lack of prescribing. [laughter]
	Day 1 workshop:<br><br>P:… Somebody goes to a cardiologist. Something that is hypertension, but they say no, I have a cold. Can you give me medicine? Probably given antibiotics and we also mentioned in that same section, profit, greed and influence...Which includes strategies such as perverse marketing…
	Interview B:<br><br>P: There is also another dimension of the issue. It is not just about inappropriate prescribing practices, but rather unethical prescribing practices where there are kickbacks for pharmacists and physician if they prescribe drugs - kickbacks from pharmaceutical industry. Thus, not all drugs that are sold are prescribed. They are sold over the counter and under the counter …

	Interview B:<br><br>P: On the medical side of things – medicine is evidence based yet that's not how things work. Health economics literature shows medical science linked with financial incentives.
	Day 1 workshop:<br><br>P: Yea and the terms for the [animal] service providers, they sometimes also sell the drugs themselves. So it is actually an important, so it is like a pervert incentive [R: yea] for them to sell, because that is often their only source of income, so they cannot charge for the service, like you know in West[ern] world, that actually your knowledge, your expertise has a price.<br><br>R: It is just the sales.<br><br>P: It is just the sales. So while they actually do [inaudible] your knowledge, at the same time there's incentive to generate income.<br><br>R: And there are two things there, and I think [researcher name] has got one. If you profit from the sales of antimicrobials, it is going to drive antimicrobial use up, and may lead to poor antimicrobial stewardship.<br><br>P: Yea.<br><br>R: But I think there is both the profiting, but there is also the linkage between prescribing and dispensing, and it can be, if you both prescribe and dispense, there is a conflict. Even if it is the right, you still have that conflict. Whereas if they are separated, you have to go from the doctor to the pharmacy. It can possibly lead to better stewardship and use.<br><br>P: And ultimately out the black door.<br><br>R: That is true I think not just on the animal side but the… .[P: aquaculture] aquaculture<br><br>P: It's exactly the same.<br><br>P: This model develops in the right [case] especially in this part, but with follow-up extension service coalition is so deep in terms of sales. Sales of [inaudible] Sales of feed, sales of the company chemicals, that is the one that is fueling need.
	Interview A:<br><br>P: you know there is price capping, there is government price capping in this region on commodities and products, livestock commodities and products and so that again adds a pressure to the system to be able to produce fast enough, the profitability. So actually I don't see that there in terms of you know government subsidies, government price regulation,

	that sort of thing. It does have, definitely has a relationship to profitability in conventional systems.
**Governance, regulations and enforcement:**<br><br>Regulations on antimicrobial manufacturing and distribution.	Day 1 workshop:<br><br>P: It might be, it might be access to the antibiotics rather than prescribing behavior. Because some antibiotics aren't prescribed at all. They can just go into the pharmacy and get whatever you want.<br><br>R: And grab, over the counter.<br><br>P: yea if they can afford.<br><br>P: yea exactly.
	Day 1 workshop:<br><br>P: It might be, it might be access to the antibiotics rather than prescribing behavior. Because some antibiotics aren't prescribed at all. They can just go into the pharmacy and get whatever you want.<br>…<br>P: And then you are talking about they probably will go to quacks, right, and not to professional doctors. That is also seeking behavior. Probably they want to mix of medicines. So for example of a specialist, pharmacologist okay. I won't mention which country, but it is not [name of country], [others laughed] where they were making these medicines then. I was curious because there were around a thousand capsules. So I asked them what, you know, what is this for, and they said, one doctor actually prescribes this. I said, why are you making a thousand. No he gives it to all the, all the patients. [P laughed] I said, what is in it. One antibiotic, one [narcotic], ones to [inaudible], one anti-inflammatory, possibly one vitamin. You are covered.
	Day 1 workshop:<br><br>P: Do you discuss the role of the quality of [antibiotics]?<br><br>R: ….So how would we… it's quality of…<br><br>P: It's quality access.<br><br>R: Do I have the direction right?<br><br>P: Quality access… like… The arrow the other way. Both. Maybe.<br><br>R: Think if the quality of the drugs has declined, how does that affect, so how do we tie that in so we make the quality of the drugs measurable.<br><br>P: You mentioned something about efficacy, future efficacy… [inaudible]

P: It ties into the future efficacy as well as the human use, and it also goes to the burden of illness.
…
P: Yea. Say drug quality.

R: Direct?

P: Yes.

P: And this is actually again common. The same issue can be exposed to animal sector.

R: Do we tie this to regulations?

P: Yes. That is also tied in.

R: We need a tie into regulations because there is the provision of poor quality or counterfeit drugs. Not control is a better way to phrase it.

P: Yea, it's not… nothing is going against it, and it is actually then linked to the access that …describe impact this, that we combine.

P: Regulations and drug manufacturers. I think that is a very large subject.

P: Because it is at every level of the use. Right, because from the drug to final take.

R: So is it regulations of drug manufacturing and distribution maybe? Then that would capture for the distribution of the drug and how it gets to the end user.

P: Yes. Exactly.

R: And that is tied to what again?

R: The quality of the drugs. So as you have more regulations of the drug manufacturing and distribution, we will have improved quality of drugs that people can access.

P: But also I mean it is at every level. So we are talking about the manufacturer. We are talking about the [inaudible] supply chain. We are talking about what can be [stocked?] in the pharmacy shop or health care clinic, because there is a classification now. We are talking about prescriber, dispenser, administrator. We are talking about the patient also. So, it's actually…

R: It is almost like a water shed node.

<table>
<tr><td></td><td>P: So, there are, there are probably things like this which need to be boxed separately and with a mark saying it actually affects [inaudible].
R: Yea, and that would be on the animal side and so we do have those sorts of all encompassing things. Okay.</td></tr>
<tr><td>Governance, regulations and enforcement:

Regulations on land use</td><td>Interview A:

P: In some ways this increasing use of certainly by the big companies, so can afford to, to use biogas units on their, particularly on their peak units, but the effluent ultimately the manure must go somewhere. Location of farms is a classic thing that probably isn't yet, is a high risk factor. For example, obviously in dairy, but certainly with pig farms, probably poultry as well, proximity to water courses, the water table, lakes, etc. etc. is increasingly important, and that is relatively unregulated I believe in Southeast Asia. So regulation in relation to farm … and the environment. I don't know if it is already captured in non-farm environmental areas, but …
…because you know in some countries mostly in [name of a country outside of Asia], you actually had to you know provide a plan of not only the location but the type of housing, etc. before that might get approved for building permits and even investment…</td></tr>
<tr><td rowspan="2">Governance, regulations and enforcement:

Role of labour costs and price structures</td><td>Day 2 workshop:

P: Yea. You see one of the, if you look at a more developed economy, compared to our developing economy, one of the key things is everything is sort of the pull factor is price. What can people afford, yea? and everybody wants cheap food and therefore they don't want to pay for a lot of you know from the cost of labour to setting up systems which you know sort of underscores cleanliness and so on. So if you really look at you know developing countries, developing economies, basically we want cheap food, so that [name of country] is very cheap relatively compared to a lot of other countries, and so people can get eat twenty-four hours a day everything is so convenient, but that all comes at a cost simply because you have got cheap labour, you know maybe immigrant labour, and you don't want to raise your base salaries, you know to a level where people can really get good people and through the system you have got to make sure along the whole production chain you have got all this traceability in place. Yea, so it is big issue in terms of how do you change the structurally in terms of pricing, in terms of the system, in terms of the policies. How do you move this? You know. So that contributes to this whole case of production.</td></tr>
<tr><td>Day 2 workshop:

P: Yea, I think in [name of country] it is a particular issue, because the level of cleanliness can change yea I mean with different requirements. Nobody wants to go to the kitchen and see what is going on, yea, but it all depends on yea.
R: Is that at every stage, like retailer down to farm?</td></tr>
</table>

	P: Yea. We have a huge immigrant population, yea, because it just lack labour you know to do these things, and we are not willing to pay the price to get good food on the table, and I think yea I mean generally we externalize the cost for cleanliness, environment, health. We externalize the cost. The government doesn't internalize the cost. If you really internalize the cost, similarly with pesticides you know if you really internalize the cost of production, then whatever food you produce is actually very expensive, because if you got to pay for the environment, if you are going to pay for [food] safety, it is going to be very, very expensive, but that is a political thing. They don't want to do that, that you know everybody is happy, yea at the end, I want to go for a very cheap meal, and so the price keeps going up, but the sad thing is where people really care for good food production, like maybe some of these organic farmers, then you really internalize the cost.
	Day 2 workshop:<br><br>The thing about one thing maybe you need to capture is the cost of using antibiotics is probably more expensive than the conventional pesticide. So farmers will not resort to using antibiotics if they have a choice. They don't have a choice. So it is a cost factor. So it negates unless your investment is high. Like citrus is expensive. So therefore you can afford to use antibiotics, but if you are using, if it is no cost, costly, and it offsets your market value, then …<br><br>P: Like the local spinach.<br><br>P: Yea, they wouldn't want to use it on a very cheap crop. They might as well use any pesticide or grow another crop you know. So it is a cost factor, and costs of pesticides is so low now, it is about 5% or less of the total cost of production, because China is now dumping so much pesticides. It is called the pesticide tsunami, basically. Oh yea. Every country in Southeast Asia is facing this problem, because China produces a dirt cheap price and so farmers can spray any amount of pesticide, and antibiotics are only coming as a last resort when they can't do anything, and that is costly, yea, because you can't simply go to a market and say, I want a bottle of antibiotic, you know no way. No way. It has got to be very, very precise. It is going to be very, very expensive. Yea.<br><br>P: So if we change the cost differential between the antibiotics and the other therapy that could change antibiotic use practices. So it would make them more expensive.<br><br>P: Exactly. So this is a regulatory thing. You have got to put our differential and it has got to be for a very premium sort of… premium sort of problems I would say.<br><br>P: Situations that exist.

	P: So human [health] is a premium you know.<br><br>P: Yea.<br>…<br>P: Oh I had a big problem. I studied in, I was in [name of Asian country], yea for a number of years. I studied there, and the first thing the doctor did was prescribe antibiotics. I said what the heck you are doing you know. This is wrong. Yea, but oh yea as you say, patients, yes sometimes. So the prize factor, differential I say negates the use. So if you put a premium on availability, in terms of cost, price, then I think it sort of negates the… how would I say the… impact of you know use in terms of extended use whatever.<br><br>R: So there are two different costings happening there. If you have a really profitable crop, then you can afford to use antibiotics.<br><br>P: Exactly.<br><br>R: But then also the amount, the price of, was it the alternatives, change …<br><br>R: Yea, other options, because I think it is not even another product. We have a situation in [name of non SEA country] where it is cheaper to give an antibiotic than to increase ventilation in the farm, so if it is just a cost thing.<br><br>P: Exactly. Exactly.
**Governance, regulations and enforcement:**<br><br>Role of different standards, regulations, traceability and enforcement	Interview A:<br><br>P: I mean international trade agreements, I mean really I see is a block and you have to consider virtually the trade of its open block. They haven't got standards that they implement really for that trade. International trade beyond near out of Southeast Asia is another thing again. Domestic standards and targets, you know some countries have them, some don't, some of course a bit, some not at all, and so there is a difference between obviously the green and standards targets and the reality, and then does it link to any other parts. Well we know the, I am just trying to think, looking at your systems. It may link to predispose individuals if related to the agriculture. So that probably comes back to that node or connection generally with livestock illness directly to humans that are involved, either handling on farm or processing them, and usually traders and transporters are independent to farms and even processing.
	Interview A:<br><br>P: …but salmonella is another one. So for example, [name of a SEA country] has a high, stipulates a higher climate salmonella free eggs and chicken, etc., and that drives you know, produces to fill that market, but it

	might mean that they focus on salmonella, but and they trade for that, driven by trade and markets,
	Day 2 workshop:<br><br>P: Okay, let me continue with the nutrition and [inaudible]. Okay. So I talk about food security first okay, because when we talk food security then we will talk about the affordability as well, okay. So what will affect the affordability is that the demand and supply. Of course, if you want all our citizens to be able to afford to buy…okay the food okay, definitely we need to increase supply. Okay, so that we can reduce cost. So how to increase supply. So definitely we want to ensure we have a good production system. Okay. We want to reduce [inaudible], we need to reduce this. So what normally the farmer or the grower, okay, in this country they use antimicrobials. So because based on my experience that when we are collecting the samples, okay, from a farm level, so we always heard about where from the farmers they say they tend to use a lot of, drugs or antimicrobials instead of just for preventive purpose. So even they use prohibited drug, okay, even they use a prohibited drug…for a number of agriculture. I am not sure okay whether they obviously want to know about it. So actually [name of country] do not have the regulations. Okay. We don't have any law and regulations to control the prohibited… [it is not really a] prohibited drug in [name of country] but [it is a] prohibited drug in [the] US. Okay. [Ma La Kut] green is actually prohibited in US or western countries, but if we check back our [name of country] Food Act regulation 1985, we don' t have any specific regulations on this [Ma La Kut] green but when we do the… [trying to find the word] study okay, we manage to identify …probably the drug, even [name of another drug] actually is a prohibited drug in [name of country] to be used in a poultry farming. Okay, so. this is the reasons why we have the AMR problems in our food system. So because okay because of course this, because of the [inaudible] because the farmer said, they cannot afford to loss, okay to loss, yea, their produce.<br><br>P: Yea farmers. So they tends to use, even if they use, prohibited drugs.<br><br>R: Okay.<br><br>P: Yea, because we don't have a good regulatory framework to control.
	Day 1 workshop:<br><br>P: Traceability is important, particularly when you know it is buyer, seller sort of arrangement. So of course if say the Japanese want to buy our eggs, you know, they have to make sure that there is traceability, yea.<br><br>P: If they have anything like some management system, this is a big part of it.<br><br>R: Certification piece.

<table>
<tr><td></td><td>P: Basically, it is a business imperative. You want to expand your business, you need to comply. So people follow ISO standard, you know traceability that is part of it.

R: Yea, so international trading, and exports.

P: Right, but internally I think very little traceability, because people don’t understand, because I think the internal regulatory system is very weak, yea. There are a lot of laws you know, regulations, but enforcement is a big issue, and like one in five hundred people you know what can you do. We are basically, yea. So you have to reduce the number of people enforced so that you can increase your enforcement capability, but you don’t let everybody go into a business and you know you will find that it is just too substantive you know for you to manage. So it is a lot of structural change in terms of, yea. So we going towards a few people producing food, way of managing that.</td></tr>
<tr><td></td><td>Day 2 workshop:

P: ….now I was in the pesticides point for many years. So I look at this original data, the distribute data, I would say about 30% of our vegetables get rejected on a monthly basis, but that is because of fungicides, you know, because fungicides have very low PR intervals, and so therefore you know your PPM levels are very low. So therefore, it gets rejected very fast. The reason being many vegetables because by the time you harvest and you bring them to market, it takes some time and fungicides prevent post-harvest, you know, harvest, fungicides prevent deterioration, otherwise farmers lose a lot. So that is the reason why they spray, but I would say that is a routine test that goes on. Yea, not necessarily because of [name of country], but of course [name of country] is very stringent in terms of checking you know random samples of [imported] things. Yea, I mean we do that very routinely in every month. Every month we develop it.

P: But is it possible because of the farmer that know, this sample is sent so that the good one to send to pesticide [inaudible]…

P: …What happens is the test of residue comes from the food regulation act by the Health Ministry. So this is done by, it is a random check in retail markets or in wholesale markets.

P: Okay.

P: It is not, you go to the farm, yes, occasionally but farmers are very smart.

P: Okay.</td></tr>
</table>

	P: So when the consignment, because it is tagged, when you have this consignment it is tagged. So there is a traceability which means you can know where the whole market comes in. The only problem is the bulking.<br><br>P: Okay.<br><br>P: Because one wholesaler buys from different farmers, and he bulks it, but whatever it is, we try to reprimand on the wholesaler. So we go to the wholesale markets, and they take samples, you know random samples.<br><br>P: Okay.<br><br>P: So you wouldn't know when they are going to come and test your samples, and so then you know that you suppose your sample is tested, then you have got a black mark on you.<br><br>R: But how can the wholesaler be responsible?... well because they tell the farmer.<br><br>P: Exactly. Exactly, because they are the link to the farmers, you know at first. So it is a political thing. It is also very political, but of course we get a lot of feedbacks from [name of country], you know because [name of a SEA country] is very stringent. Those days people use to bluff. They do all kinds of freaky things, by putting the good baskets outside and the lousy baskets inside. Now the [name of SEA country] authorities say, get everything out. We will do random sampling and you know they do random sampling and if they catch anything, they will just make a phone call or send a report.<br><br>P: How come, last year, because letters, okay, the letters actually have been rejected by the [name of SEA country], but in the end the rejected letters, okay, sells in your home.<br><br>P: Yea, of course. If you find good letters in your home market that means you know that it has been a rejected sample. Yea. Don't worry you can go and eat in [name of a SEA city]. You should worry about, you should worry about salads, you know, because …<br><br>R: Wait why.<br><br>P: No, no because they said, there was a quick test by these Australian scientists from University of Queensland, even around randomly picking up samples you know from markets.<br><br>P: Supermarkets or wet markets.<br><br>P: Supermarkets are fine, but the wet markets.

	P: Okay.<br><br>P: And many of these stalls, many of these shops, they buy from wet markets. Yea.<br><br>P: Yea.<br><br>P: And farmer markets, and a lot of them are contaminated with e-coli and that kind of stuff, because people use all kinds of organic manure.

OVERARCHING FACTOR: Climate change

**Climate Change:**<br><br>Climate change impacts	Day 1 workshop:<br><br>P: …That is also a key thing. I mean we are talking about, this is where policies may differ. No policy is going to judge any farmer who is existing for survival both for families' sake and for individual's sake. They are already in debt. Nobody is helping them. Yet climate change is affecting everything. There are pests and insects. All that they just have to survive… and they have limited space, crowding, you basically say don't crowd them, but they will crowd. There is not a space left.
	Day 1 workshop:<br><br>P: What about climate change.<br><br>R: Climate change we have captured. We have been treating that one as an overarching affecting everything, but do you agree with that?<br><br>P: Yea, because it changes for all ….<br><br>P: Could it be like a good study, the climate change and see how it affects AMR.
	Day 2 workshop:<br><br>P: …our system is failing us, and it is going to kill us by the year 2050. Again, scientists are warning us. We will start to have food insecurity for crisis. With the climate change, agriculture, livestock, and so on. The climate change, the farmer has got to find ways, you know. It is not an immediate fix you know. Right. They are not wiping off one acre, two acre. It can be an entire thing, and the worst is coming. The last few years you can see that the climate is so severe you know.
	Day 1 workshop:<br><br>P: Then you have got water scarcity issues. That leads to drinking, whether it is in a village or a city. That leads drinking difficult water. Let's put it that way. That leads to diarrhea. That leads to again health seeking behavior.<br><br>P: Right.<br><br>P: You have got the same issue when you, if you want to wash your vegetables or you cook, you don't have the proper water. Okay. Rivers are drying up. Temperatures are varying nowadays. So farmers offer normal people the same issues affect directly now the environment.<br>…<br>So and the other thing there is I have a feeling, I mean I was, I am not sure about it, global warming is changing the acidity of oceans. Therefore the

	so called microbiome in oceans is changing. So therefore I don't know how it affects fish, but that is another issue altogether and that is what is going up into the clouds and then causing rain. So acid rain and what not. Okay coming down into the soil and that is where we are growing our vegetables and getting water.<br><br>P: The links within climate change and the environment are ….<br><br>P: Actually you just need to push the case a bit more.<br><br>R: Yea.<br><br>P: Yea so the water cycle is influenced by climate change.<br><br>P: So the whole biosphere, if I can put it that way is actually [inaudible] prevention. We as humans don't realize ….
	Day 2 workshop:<br><br>P: We are basically copying the American model, as we progress [economically], we get larger and so on, I don't think taller. [laughter] So I think we need to inject education everywhere into people. While we start to do all these things you know, because all these also affect climate change you know. Food waste in the land fill emits methane gas, you know, and the resources, the [inaudible] field that need to go into agriculture. It is all lost, you know, the arable land, the water, and all this affects you know…
	Interview A:<br><br>P: I guess another aspect that maybe isn't considered here, but relates to probably the whole system as you say are other things like climate change…I was speaking to the]name of organization] person based in [name of SEA city] recently and you know the impact of that not only obviously, but will be you know initially heat stress and heat stress on animals and farms - pigs and poultry being the most vulnerable - and that will of course increase their susceptibility and sickness and mortality, etc. etc., and then might change the dynamics of endemic disease and other things. So yes, we don't entirely know how that is going to play out in this system, but I think it is certainly important,

OVERARCHING FACTOR: Underlying intent of the system

**Underlying intent of system:**<br><br>A consumptive economy mindset drives system behaviour and this plus a lack of systems thinking impacts addressing root causes of disease.	Day 2 workshop:<br><br>P: …our system is failing us, and it is going to kill us by the year 2050. Again, scientists are warning us. We will start to have food insecurity for crisis. With the climate change, agriculture, livestock, and so on. The climate change, the farmer has got to find ways, you know. It is not an immediate fix you know. Right. They are not wiping off one acre, two acre. It can be an entire thing, and the worst is coming. The last few years you can see that the climate is so severe you know.<br><br>P: I think we are in a very consumptive economy.<br><br>P: Yea.<br><br>P: Everybody wants different things, huh. So it is a total mindset change. The economy is a very consumptive base, but it is big business. You cannot run this world without a business, because the business guys are the third force yea. The invisible force.<br><br>P: You become like oil, you will be in control.
	Interview A:<br><br>P: but somewhat yea frustration, people aren't thinking about how to improve the underlying risk factors to, that drive endemic disease…
