# Supplementary material for: Factors impacting antimicrobial resistance in the South East Asian food system and potential places to intervene: A participatory, one health study
Source: Front Microbiol. 2023 Jan 5;13:992507. doi: 10.3389/fmicb.2022.992507 (PMC9849958; doi:10.3389/fmicb.2022.992507)
Supplement: Supplementary file 1 [file Data_Sheet_1.zip › Supplementary File C.pdf]

SUPPLEMENTARY FILE C: QUOTES PER LEVARAGE POINT

Acronyms:

P: Participant
R: Researchers that facilitated discussions
SEA = South East Asia
AMU = Antimicrobial use
AMR = Antimicrobial resistance

Please note:

1. Some quotes were modified in instances to protect the identity of participants or the organizations they represent.
2. P: represent a participant. Some quotes contain multiple P's. This means the quote reflects a discussion thread involving a participant and the researcher or multiple participants.
3. A given quote may be coded in more than one theme if it applies to each.

LEVERAGE POINT: MULTI-FACETED APROACH

**Multi-faceted approach is needed. Not just one leverage point or one intervention**	Day 1 workshop:<br><br>P: No, I mean one single action may not be able to push it, so maybe I see a different node multifaceted.<br><br>R: Multi-faceted.<br><br>P: Yea.<br><br>R: Is that the intervention or the leverage point<br><br>P: The leverage point.

LEVERAGE POINT: National budgets, funding, money

**National budgets, funding, money**<br><br>Investments in developing alternatives to antimicrobials	Day 1 workshop:<br><br>Second thing hard to do. It can be significant [inaudible – too much background chattering] research and development mainly focus on vaccines.<br>[R: vaccines, regarding] for the key business, humans and maybe [inaudible] [R: Okay, humans, animals, livestock]. That would be a significant impact on the usage [inaudible] investment in R&D for a lot of these vaccines [inaudible] 99% reduction in antibiotics usage because of [inaudible] only because of vaccines.
	Day 2 workshop:<br><br>P: And then I think back to back I think one of the things that you brought up about developing alternatives. If you are taking out a particular solution, then you have to find alternatives, you know viable alternatives.<br><br>P: Because if you got alternatives to some of these problems you know to solving these problems, then I think people will not want to resort to this.<br><br>P: Yea.<br><br>P: To reduce that situation of desperation you know.<br><br>P: So perhaps better technologies for the farms could be disseminated<br><br>P: Exactly.<br><br>P: Yea, and so that comes with a lot of government investment into R&D.
**National budgets, funding, money:**<br><br>Investment in training and capacity building	Interview B:<br><br>P: Investing in and creating policies for training of health care professionals on stewardship.
	Day 1 workshop:<br><br>P: The crops, because there is often not crop health professional.

LEVERAGE POINT: Resistance in the wider environment

**Resistance in the wider environment**	Day 2 workshop:<br><br>P: If we assume that the water is one of the vehicles, of the channel, to a spread of antibiotics and antibiotic resistant bacteria, then what can we do to prevent water pollution by antibiotics and antimicrobials…<br>…<br>looking at pollution control from farms, something has shown to be effective is to install green buffers. So vegetation areas around farms or around water bodies to prevent this defused pollution. This run off that could potentially bring antibiotics in and so on, reaching a water body. So in wastewater treatment, because then wastewater, wastewater is where all these antibiotics can concentrate coming from cities, then when an antibiotic takes resistance bacteria comes from farms, one can install this green buffers along rivers or around farms, and then once water is polluted then drinking water treatment could be one of the ..<br><br>R: You are talking about intervention.<br><br>P: Yea.<br><br>R: Are these in place at all? These preventative measures going from the farm to the water.<br><br>P: Yes, but these are typically not in style to deal with antibiotics in particular. So the green buffers exist, do exist…

LEVERAGE POINT: Governance, regulations and enforcement

<table>
<tr><td>Governance, Regulations and Enforcement:

Strengthen regulatory control over the development of antibiotics for agricultural use.</td><td>Day 2 workshop:

P: I think the… yea I mean it is, this is specific to agriculture, but agriculture meaning crops, yea, presently exists, but not necessarily from a veterinary point of view and also. So that was what was brought up by your earlier participant, the veterinary, the animal industry.
…

R: Oh…The pharmaceutical person.

P: Yea. So he was saying about you know this problem of you really do not know a lot of people are following practices which are not very well scientifically proven in terms of [antibiotic] design. In terms of [gastrointestinal] tract design. In terms of use. And also in terms of you know the, what are the side effects and so on. So there are many things that are really not so clear.</td></tr>
<tr><td>Governance, Regulations and Enforcement:

Access to antimicrobials - Regulating and enforcing what categories of antibiotics can be used.</td><td>Day 1 workshop:

P: [inaudible] covers the leverage point, [and the influences coming] regulations through the leverage points. The first one was the regulation and policy. Very high impact but [inaudible] in my opinion easy to do [inaudible] enforcement.

R: So regulations and enforcement.

P: Especially for example if you look into some priority antibiotics, the one like [P's name] was telling, control production, control distributions from different categories, what's available to which group, how to use… So, stringent regulations and a policy and that is for [inaudible] especially in the low- and middle-income countries. That is one.</td></tr>
<tr><td rowspan="2">Governance, Regulations and Enforcement:

Access to antimicrobials - Registering antibiotics that are legal for use.</td><td>Interview B:

How to intervene to stop fake drugs.</td></tr>
<tr><td>Day 2 workshop:

P: We have something here that, I don't know. Tell me if I am saying it right, but the government of [name of SEA country] three years ago.

P: A couple of years ago.

P: A few years ago stopped registering antibiotics, meaning it cut off farmers from accessing them.

P: From companies selling them or registering them, because the …[once you] registered them, you can use them legally. So in 1974 when the [inaudible] act came into being, there were about fifteen to</td></tr>
</table>

	sixteen different types of antibiotics used against bacterial diseases and also fungicides, because some of them do have some for anti-fungal you know properties, but recently after that review, which we did, and subsequently I think they put it into an act, and now if you go into the web site, registered pesticide website, there is nothing. It is all removed totally. The same thing with [name of another SEA country]…three years ago, three years ago they were using, you know antibiotics. It is quite common for Southeast Asian countries to use antibiotics, because it is easily available in retail shops.<br><br>R: But now you are saying it is not.<br><br>P: Now, no. Government doesn't officially have a list of antibiotics that can be used in agriculture.<br><br>R: So…it is like a black market thing?<br><br>P: It is a black market thing. It is not legal yea. It is not a registered list, because a registered list you can use, yea.<br><br>P: …pesticides go into you know black market you know. So a lot of unregistered pesticides are also being sold, but that is all cross border trade and that kind of thing. But yea, but that is like a big barrier in [anti]microbial use. But it is very sensitive to talk to a farmer you know. Governments may not like it, because they think that they are doing their job, but I think we know on the ground that it is not working.<br><br>R: So is the leverage point like government regulations.<br><br>P: Policy driven. Yea.<br>…<br>P: Huge, yea, that is a huge, because the idea I think what we are trying to say here, is more in terms of curbing the source. You try the supply. You cut the supply and the source. Then all along the production chain, along with the maybe, the awareness and education.
**Governance, Regulations and Enforcement:**<br><br>Access to antimicrobials: Regulating the manufacturing, distribution and sale of antimicrobials (e.g., ban over the counter sales)	Day 1 workshop:<br><br>P: You mentioned something about efficacy, future efficacy… [inaudible]<br><br>P: It ties into the future efficacy as well as the human use, and it also goes to the burden of illness.<br>…<br>P: And this is actually again common. The same issue can be exposed to animal sector.<br><br>R: Do we tie this to regulations?

	P: Yes. That is also tied in.<br><br>R: We need a tie into regulations because there is the provision of poor quality or counterfeit drugs. Not control is a better way to phrase it.<br><br>P: Yea, it's not… nothing is going against it, and it is actually then linked to the access that …describe impact this, that we combine.<br><br>P: Regulations and drug manufacturers. I think that is a very large subject.<br><br>P: Because it is at every level of the use. Right, because from the drug to final take.<br><br>R: So is it regulations of drug manufacturing and distribution maybe? Then that would capture for the distribution of the drug and how it gets to the end user.<br><br>P: Yes. Exactly.<br><br>R: And that is tied to what again? The quality of the drugs. So as you have more regulations of the drug manufacturing and distribution, we will have improved quality of drugs that people can access.<br><br>P: But also I mean it is at every level. So we are talking about the manufacturer. We are talking about the [inaudible] supply chain. We are talking about what can be [stocked?] in the pharmacy shop or health care clinic, because there is a classification now. We are talking about prescriber, dispenser, administrator. We are talking about the patient also…
	Day 1 workshop:<br><br>P: So I think one would be the possibility to ban over the counter sales. I guess that is something we know, so we understand the channels, the marketing channels for antibiotics. So there is something that we can do about that, but they are, obviously then there is the whole questions about access versus excess and we discussed it yesterday already, because in a way, not representing very well the poorest in our society, when I heard something like that, because they actually need to have access to drugs, but then that is maybe another problem to be solved through other means, but I think the risk by having too much over the counter sales, almost outweigh that, so I think it is a bit of a cost benefit…And I think to make sure that this is even possible to work. We were sort of saying, okay, but then what are the alternatives present, when people do go, and for drugs, so at least there is something else that they can get. And I am not convinced about that. That is big problem.

<table>
<tr><td></td><td>R: So it is policy change.

P: Yea.

R: Yea. So you have distribution channels. You need to be cognizant of people who are in need to live in rural areas.

P: [inaudible]

R: They don’t have access. I am just repeating just to make sure [the audio-recording captured it.

P: Yea.

R: So there is access to antimicrobials for them.

P: Yea.

R: It is not taken completely away.

P: Yes.

R: But also a need to provide alternative to antibiotics. Any strategies for trying to make this happen?

P: Well I think it would be, well getting the alternatives, having the right policies in place, and being able to enforce it and I think it relates to what was saying before, this was strictly used. This is going into the same, it could be one intervention that you were mentioning, but the problem is how to enforce policies in that.
…
P: I think it would be high impact, yea, because it actually affects all the sectors, it’s livestock, aquaculture, human health.

P: If you could include online pharmacies also.

P: Exactly, that would be over the counter. Yea.</td></tr>
<tr><td></td><td>Interview B:

putting regulations in to regulate where individuals can access drugs and have enforcement to back it up.</td></tr>
<tr><td>Government, Regulations and Enforcement:

Access to antimicrobials-Regulating who prescribes and decoupling antibiotic sales from prescription.</td><td>Day 2 workshop:

R: And one other comment [made is] about that preventive use, one of the ways of making it, and proving the stewardship is if things are through the veterinarian, so then the veterinarian can make a decision on whether the prevention is needed as opposed to direct access by the farmers purchasing it.</td></tr>
</table>

<table>
<tr><td></td><td>P: Yea.</td></tr>
<tr><td></td><td>Interview B:

Making sure the culture of kickbacks don't happen. Pharmacies should be regulated to not get money for drugs that they do not give prescriptions for.</td></tr>
<tr><td rowspan="2">Government, Regulations and Enforcement:

Setting, monitoring and enforcing antibiotic policies and guidance, AMU limits and targets, benchmarks.</td><td>Day 1 workshop:

P: Yea, the other things I have written down was antibiotic policies and guidance in reducing antibiotic use, monitoring use. I think that is probably covered in a lot of the training that we talked about earlier and ….

R: Guidelines. Is that part of it?

P: Health, infection, prevention, and control guidance. Guidance is the important element of that.
…
R: We had like the guidelines and then we had enforcement of guidelines.
…
P: Okay. Good.</td></tr>
<tr><td>Day 2 workshop:

P: Basically a lot similar to what they have done. One of these is regulations as well. This morning we were talking about the lack of specific limits of antibiotics in certain food. So that has to be directed and this comprises everything for meat, for vegetables, for water. Right, and coming down from that, we need specific policies. Sometimes they do regulations on including they can do a lot, but not enough on specific volume to actually focus on that.
…
P: Regulation is the intervention that a specific thing that…the government need to do is the establishment of specific limits of antibiotics.

R: Of use of antibiotics?

P: In how much they can actually put in. They do have it now, but as [P's name] mentioned, it is not so specific, not really for the entire food chain, just on some specific, some sporadic foods maybe.
…
R: Okay. Are you thinking more of the processing - antibiotics through like the actual [inaudible]?

P: Yea, exactly.</td></tr>
</table>

	…<br>R: And slaughterhouses?<br><br>P: Slaughterhouses and for those people that are processing.<br><br>R: Manufacturing.<br><br>P: Manufacturer, because they are the last ones.
	Interview A:<br><br>P: …meeting targets…
	Day 1 workshop:<br><br>P: I think for the industry you know, the measurement that we can do is the amount use of antibiotics reduced in the productions, but when we are talking about AMR, what is the measurement? And, when we reduce using antibiotics into a certain level, how can we say this is success or not success [in reducing AMR levels] and then that targets standard of benchmarking for the industry to understand that we achieve or we are not achieving. So this is quite unclear for me. Because I think it is kind of parallel issue antibiotic use and AMR, but at the moment we are talking about AMR, but implementation is reducing using antibiotics. How can we make those two compare to each other?
	Day 1 workshop:<br><br>P: I can give you one bit that put into the program. Actually at this first stage, [name of organization] support[s] countries to generate data. So I mean to understand what is the antimicrobial resistance level in these countries and also to understand what is the pattern of antimicrobial usage in different, I mean, human and animal and both. It is these are evidence to be taken into consideration at country level to develop their policies, so… the member states [are expected] to use this data to redefine their policies or take immediate actions to modify the recommendations of policies for those in their countries so that is the immediate help that is [inaudible] by the UK government. So [name of organization] are implementing all these [inaudible]. So I think that it is very extensive intervention and that has to be used.
**Governance, Regulations and Enforcement:**<br><br>Animal welfare:<br>Standards and regulations	Interview A:<br><br>P: we can't emphasize enough the importance of improving animal welfare, decreasing stress, decreasing the vulnerability and susceptibility to disease in farm animal systems and farming and food systems more broadly.<br>…<br>P: wet markets for animals – a high risk area for stressed animals shedding high levels of faecal bacteria and AMR. I can't stress enough the major issue of informal and inhumane slaughter, even in 'formal' settings, in SEA – it is truly a hot bed of AMR risk for workers and food

	systems – aside from cruelty, which is documented for various zoonoses also. A lack of knowledge, training, standards and regulation persists….
**Governance, Regulations and Enforcement:**<br><br>Requiring certifications for people who produce food to ensure good production practices and safe food	Day 2 workshop:<br><br>P: …. I mean generally we externalize the cost for cleanliness, environment, health. We externalize the cost. The government doesn't internalize the cost. If you really internalize the cost, similarly with pesticides you know if you really internalize the cost of production, then whatever food you produce is actually very expensive, because if you got to pay for the environment, if you are going to pay for [food] safety, it is going to be very, very expensive, but that is a political thing...<br>…<br>P: You put so much of you know systems in place, you know I want you to do this. I want, even composting chicken manure has got more antibiotics…so everything is so stringent, you know in a system where people are trying to produce good food. I actually came out with a solution last time when I was in the port. I told them that why is that, you know every other profession you have people need to be certified. Okay, in [name of country], we don't certify everybody. We don't certify our plumber. We don't certify electrician, although you need to have a certain grade, yea. People will produce food for the masses. They should be certified…<br><br>P: Yea, at the moment the food analyst, those people testing, they have food analyst [inaudible], [P: yea, yea] but person producing the food …<br><br>P: Anybody can. It is an easy entry market. Anybody tomorrow I feel like becoming a farmer, I just become a farmer. I don't have to have qualification. I just go and produce anything. So these are tricky issues, yea. If we don't pay attention to this kind of details. Yea.
**Governance, Regulations and Enforcement:**<br><br>Implementing traceability systems that are enforced (requires) increasing enforcement capacity	Day 2 workshop:<br><br>R: You mentioned the traceability piece, …tracing … the whole system and I captured it, as assuming it is not happening. It doesn't really happen much. I wasn't quite sure if there is anything more you wanted to say about that? …<br><br>P: Traceability is important, particularly when you know it is buyer, seller sort of arrangement. So of course if say the Japanese want to buy our eggs, you know, they have to make sure that there is traceability, yea.<br><br>P: If they have anything like some management system, this is a big part of it.<br>…

	P: Basically it is a business imperative. You want to expand your business, you need to comply. So people follow ISO standard, you know traceability that is part of it.<br><br>R: Yea, so international trading, and exports.<br><br>P: Right, but internally I think very little traceability, because people don't understand, because I think the internal regulatory system is very weak, yea. There are a lot of laws you know, regulations, but enforcement is a big issue, and like one in five hundred people you know what can you do. We are basically, yea. So you have to reduce the number of people enforced so that you can increase your enforcement capability, but you don't let everybody go into a business and you know you will find that it is just too substantive you know for you to manage. So it is a lot of structural change in terms of, yea. So we going towards a few people producing food, way of managing that.
**Governance, Regulations and Enforcement:**<br><br>Instituting taxes or increasing prices to influence production patterns.	Day 2 workshop:<br><br>P: And that is an issue we have too is that people want cheap, say chicken. They want cheap chicken breasts, they will buy the cheapest. They don't know the education behind why it is cheap.<br><br>P: Why yes.<br><br>R: And there needs to be an education piece on, well if we stop using antibiotics we need to maybe put more money in, chicken might go up in price.<br><br>P: Exactly. Exactly. Yea. So you stick back to your battery system that you want you know chicken you know to produce at the lowest cost, so that you have got this, you know consumers you know who can buy cheap chicken, but people go through so much in a developing economy, in order to get that food on the table and it costs you so much.<br><br>P: So link to these, maybe quantifying the cost of antimicrobial resistance, would allow internalize these costs in production.<br><br>P: Yea.<br><br>P: So that chicken production using extra antibiotics doesn't become so cheap.<br>…<br>P: Because they need the room for taxes to internalize the cost of this misuse of antibiotics.
	Interview A:

	P: The tax aspect is interesting – it could include also relate to the AMR as an externality better incorporated into costs by those that irresponsibly sell or use them or buy related products.
	Day 2 workshop:<br><br>P: Yea, the thing of changing diets, more sustainable and healthy diets.<br><br>R: Oh, that will be fun.<br><br>P: But then we agreed kind of that it was not enough with increasing awareness of consumers to change behaviour and consumption patterns, and your diet. So it would have to be brought together with policies like taxes that make you know unhealthy or unsustainable diets more expensive, and then under the assumption that changing diet will drive the market, will drive production, will change production as well. Production patterns.<br><br>R: Okay. So take me back. So we need taxing.<br><br>P: Taxing. Yea.<br><br>R: Sugars and all that.<br><br>P: Well yea.<br><br>P: Those taxes go over real well. [laughed]<br><br>R: You need to tax food.<br><br>P: Yea, and higher fee, I mean you know with the ultimate idea to influence the production patterns, including the use of antibiotics, and then it is not only about what we thought. It is not about, it is not only about awareness, but also taxing. We didn't discuss anything else.<br><br>R: … Okay. Any unintended consequences of taking this sort of approach?<br><br>P: Well if it affects the pool or those that are in the demand, that are still not satisfying their nutritional needs, then it is an unintended. I mean if we are trying to minimize the use of the consumption of meat, but there are specific [cultures] or people that actually need to require more meat. So if these changes is taxes, these taxes for instance, make it impossible for some people to access meat when they need it then it will be bad.<br><br>R: So they can't afford it.<br><br>P: Yea.

	…<br>Reduces accessibility.<br>…<br>R: … Okay, and by decreasing unhealthy food patterns, this should help with decreasing antimicrobial use.<br><br>P: In the production.<br>…<br>P: I mean we use the total amount of meat that needs to be produced in consequence.<br><br>R: Right. It is about meat.<br><br>P: Yea.
	Day 2 workshop:<br><br>R: … So you talked about a need, awareness and education isn't enough to create change in behaviors. We need taxing of unhealthy foods and practices for kind of, which is use. So taxing the production, taxing meats, animals. Okay, but through that process this should help decrease, I guess consumption of it.<br><br>P: Demand for it.<br><br>R: And demand. Yea, decrease demand.<br><br>P: Thus decrease production.<br><br>R: Yea.<br><br>P: So, what's a good idea?<br><br>P: Sugar, sugar tax, similar idea. Tax those from production sectors, which are …unfriendly, which are vicious, which are… unwilling to contribute [inaudible]. Tax them.<br><br>P: Yea, it is much easier said than done. They face a lot of opposition.<br><br>R: So that is a quite nice …<br><br>P: Argument is the same point, isn't it? Otherwise you see the food lobby, the way….<br><br>P: That's why it's so hard, they fight…<br><br>P: The agricultural sector is pushed to one side. It is the same argument that is used. [inaudible] It is different arguments especially where they

	are positioned now, with [inaudible] resistance. It is very bad trust and publicity<br><br>R: So a challenge is getting this on certain food systems. Is that what it is?<br><br>P: Yea, I think [inaudible] whether it's best food, bad food. Tax all those which are bad.<br><br>R: I can ask a challenging question here though.<br><br>P: Yea, but this is, there are implications to the socioeconomic you know system. Yea.<br>…<br><br>P: Taxing you know, you know, you got to be careful about taxing.<br><br>P: Yea, we didn't mention that in the conversation.<br><br>R: It might impact the people need to decrease.<br><br>P: Yea, because it all depends on your socioeconomic status and who will be able to be taxed and whatever.<br>…<br>R: A challenge question to this. Does the same thing apply to the animal side of things? Taxing for poor diet for animals. [P: oh definitely] Poor quality feed which should be taxed.<br><br>P: That should be an option.<br><br>P: But I think it is a very good point. We could tax those fishes are produced, which are put in fish meal.<br><br>P: Commercial.<br><br>P: Which is wasting nutritious fish. We could tax them. All those commodities which are obviously they are not primal productivity, they lower the food web. Those that are depending on fish meal. [inaudible] So you tax that sector …<br><br>P: And push people to raise fish that aren't reliant on fish meal. [P: Yea, that is actually a really good point.]<br><br>P: That is a very good point. You cannot, feeding cat fish to [inaudible] fish. People feed [inaudible] fish to [inaudible] fish. So that system can be taxed.
	Day 2 workshop:

	P: Yea, and we talked about the taxes as well.<br><br>R: Yes.<br><br>P: So that we tax on antibiotics.<br><br>R: So this is part of regulations?<br><br>P: Yes. Still regulations.<br><br>P: They are legal.<br><br>P: Yea they are legal, but the same thing with sugar, it is legal but if you use more.
	Day 2 workshop:<br><br>R: …we are talking about the need to tax use of antibiotics for use in livestock and aquaculture is a possible way to reduce use, and is that, I think that, do you have anything to add to that? Is that something that is viable or happens in Southeast…?<br><br>P: It is viable provided the antibiotics are available in the market. The personal value of the stock, shrimp farmers so they will go great extent to buy this.<br><br>P: It is not market value as I said, yea. It is only used for specific high value crops, high value productions, because it is expensive, you know. So you don't want to use it as a very broad you know use of. They must use other alternatives. Yea.
	Day 2 workshop:<br><br>P: So you are saying that monoculture are the drivers of AMR, or poor…you know, biodiversity has been lost. Could the tax could be applied on farmers. They are paying more tax, and incentive is to ask them to diversify the species and the nutritious fish. Not the protein. The protein you are not interested. You are interested in the micro-nutrients. So may the tax be having an impact on ..? like a drive on the…, because I mean we don't need more protein. We need more micro-nutrients, and I do believe that monoculture is a drive to disease and …<br><br>P: And decrease micronutrients intake<br><br>P: That is the big problem. Commercial. Yea.<br><br>P: Because the farming regulations in the literature, they are probably having the system that we are not talking about them and this is what is

	important. This is a mission [of name of organization], right? To help these people to access those protein, or those micronutrients.

LEVERAGE POINT: Prescribing, diagnosis, treatment practices

**Prescribing, diagnosing, treatment practices:**<br><br>Instituting and enforcing AMU guidelines and policies to improve antimicrobial stewardship	P: Yea, we can start. We talked about several things. The first one was restrictions on the use of antibiotics in humans and you can take it much more broadly as well.<br>…<br>Intervention would be introductory, introducing guidelines either in hospital or nationally or national association.<br>…<br>We went up with a list of antibiotics which are allowed, antibiotics which anybody can prescribe.<br><br>R: Is this for human use only?<br><br>P: This is, my experience is human use, but it could be done for veterinary use, other areas too and then you have a second and third line, list of antibiotics, which are available, but you have to have very good reasons to use them. So the second list might be where you use them, you know [when] there is antibiotic resistance and infection.<br>…<br>If you thought, if you have an infection in front of you, where there is likely to be antibiotic resistance, urinary tract infection in humans and you can use the second line, but you have to have good reasons to do so, and the third line will be the antibiotics which are effectively prohibited unless you authorization to do so and somebody is in control.<br><br>R: And who would that be …<br><br>P: In a hospital, that would be one of the very senior pharmacists or a very senior microbiologist or specialist depending upon but somebody senior with specific responsibility for controlling antibiotics. The same principles apply to all sorts of drugs actually. Doesn't just have to be antibiotics but that is obviously what we are talking about here.<br>…
	Interview B:<br><br>P: Investing in and creating policies for training of health care professionals on stewardship.
	Interview B:<br><br>P: Policies of utilization review – hospitals do quality reviews of their practice to ensure quality of care, and this can provide an opportunity

	to look at prescribing practices. Important to monitor this as part of quality of care.

LEVERAGE POINT: Treatment of waste and wastewater (e.g., sewage, manure, sludge)

**Treatment of waste and wastewater (e.g., sewage, manure, sludge)**	Day 2 workshop:<br><br>P: Yea, well in developing countries wastewater treatment you know it is very, very rare, effective water treatment is very rare. So all these wastes get into waters untreated. So it becomes more important. The prevention of exposure. Yea, drinking water treatment, or yea basically drinking water treatment it costs less. You don't have any interruptions. Once water is polluted you either treat the water before drinking, or …
	Day 2 workshop:<br><br>P: If we assume that the water is one of the vehicles, of the channel, to a spread of antibiotics and antibiotic resistant bacteria, then what can we do to prevent water pollution by antibiotics and antimicrobials, so where is water treatment would be one of the things to consider and the ones, water is already polluted with antibiotics, and antibiotics drinking water treatment…then once water is polluted then drinking water treatment could be one of the ..<br><br>R: You are talking about intervention.<br><br>P: Yea.<br><br>R: Are these in place at all? These preventative measures going from the farm to the water.<br><br>P: Yes, but these are typically not in style to deal with antibiotics in particular. So the green buffers exist, do exist, and wastewater treatment plants do exist, and drinking water treatment plant do exist, but they are, but they are there to prevent the pathogens, but if they are affected by normal pathogens they would be also affected for antimicrobial resistant pathogens.<br><br>R: Yea.<br><br>P: But they are not affected for its genes, so they can you know decompose bacteria and… the cell itself, but the genes get released and then they still are there, both in drinking water or in treated wastewater…

LEVERAGE POINT: Awareness and understanding

**Awareness and understanding:**<br><br>Any change to baseline knowledge is important even though insufficient to change behaviour	Day 2 workshop:<br><br>P: I think so, you know because knowledge in this case is not enough for them to change.<br>…<br>P: Yea, I defer in terms of the impact factor, simple because at the moment it is sort of a zero level. So anything that you change makes an impact, huge impact. That is what I am saying there. It is not a very marginal thing, because at the moment very few, I mean I am seeing it from the baseline, yea? What is there now? Very little.<br><br>P: Right.<br><br>P: So whatever you do in terms of educating people, making people understand, I think makes an impact.<br><br>P: Impact yes, but we are asking big impact, or low impact? I think it is somewhere in the middle.<br><br>P: It is right in the middle. Yea. We should have another category. Yea.<br>…<br>P: What do you say was zero?<br><br>P: No at the moment there is not much, many of the people, practitioners here that people were using the antibiotics, do not see the consequences of its use in terms of resistance or in terms of the medical implications and so on. So it is all floating up there. You know it is not right to the ground.<br><br>P: Yes, I agree. But in terms of the better practice and advocacy and performing practices, both in livestock and fish, there has been [inaudible] literature and education materials, programs, but in terms of impact, I have no answer. but in terms of the messages and material we have worked for the last twenty years, since the [inaudible] have developed, a lot of things that we have done.<br>…<br>P: So I would not agree that there is no… if you look carefully at the better practices, right from the, if you were a doctor or an official, all these basic guidelines, right from the field, up to [inaudible] no, no, no. This message has gone up in the last thirty years.<br><br>P: [group chatter] The difference between creating [inaudible] and …<br><br>P: It is reaching the farmers.

	P: It is reaching the farmers, but not near the chain in terms of the behaviour and their practices. The important thing is has the practice changed. Has the behavior changed?
	Day 1 workshop:<br><br>P: And the low impact was … education, awareness, campaign, best practices. All that. Easy to do. Hundreds of agencies are already doing it. The impact is still, we are not seeing it, it's very difficult to see the impact. [Inaudible] [name of organization] [inaudible]. . Everybody is learning the message though. But still the impact we are not seeing it. There's no [inaudible]. No indicators are to showing that it is working.<br>I will give you an example. Aquatic [inaudible] all the fifteen businesses, pass it to Atlantic to [inaudible] in spite of all international markets standards and interventions in place. It has not helped. Still spreading. Same as [Ebola] for example. So it is very hard to do. [inaudible] …It's a big intervention area.
**Awareness and understanding:**<br><br>Media	Day 1 workshop:<br><br>P: The link to this (reaching different target audiences such as consumers, decision-makers) I think that influencing the influencers would be important, and then we are all influenced by media. So training media or getting our messages through media, but really reach the people and create this awareness I am starting to see the issue of antimicrobial resistance in Spanish, but I don't know if it is everywhere, and it really creates this feeling of urgency, targeting the media.<br>…<br>P: I mean there is the world awareness week coming up soon. So that is one way.<br>…<br>P: We have committed quite a few media workshops. Interestingly many other media think that, initially at least that you know just like they want us to, they want direct recognition. They think that we invite them, that it is just for our recognition mostly right, so it takes some time for them to understand that. I actually told them during our workshop, please do not mention our names at all. Okay? but you only to write an article about someone, but just understand it, that when these things happen, you know exactly what to do and what to write.
**Awareness and understanding:**<br><br>Consumer choice, demand and behaviour (including youth)	P: Capacity development, behaviour change<br><br>P: Behaviour change. Yea.<br><br>P: So it would be to create either incentive, but also otherwise sort of work on fear and I think it probably sort of work in a direction of the shaming of antibiotic use, sort of creating that perception that any o save lives.<br><br>P: The misuse. Shame of misusing antibiotics.

	P: Yea.<br><br>R: antibiotics?<br><br>P: Misusing.<br><br>R: Of misusing antibiotics.<br><br>P: They are talking about, they mentioned climate change. Now we all feel bad that we are actually were flying half way around the world to be here, so I think sort of the consciousness is becoming more prevalent, that people are aware of how bad flying is. So we also need to start creating that awareness, how bad [misusing] antibiotics are. Social responsibility.
	Day 2 workshop:<br><br>P: Increasing public awareness of the dangers of AMR such as the campaign of healthy livestock products for consumption and clean and healthy life behaviour from an early age.
	Day 2 workshop:<br><br>R: And there needs to be an education piece on, well if we stop using antibiotics we need to maybe put more money in, chicken might go up in price.<br><br>P: Exactly. Exactly
	Day 1 workshop:<br><br>P: Yea, because this kind of linkage to the number one, consumer, sometimes they don't have an idea what is AMR. So they didn't pay attention and they didn't put the demand to the producer. So it is kind of, if they don't know that this is important to them, so we think kinds of awareness and knowledge and things.<br><br>R: So awareness and knowledge can also influence that.<br><br>P: Yes, but without their knowledge and awareness, sometimes they don't create the demands.<br><br>P: But because of lack of the good biosecurity, that is why it is forcing them a little bit to either use the antibiotics or alternative for treatment. Right, but the most difficult for them is biosecurity is not only management, it is an investment.<br>R: Okay.<br><br>P: So many of them, they don't have financial enough to invest , investment in good biosecurity n the farms. So that is why I think these

	three multiple areas are kind of linked together. The consumer know, the public want the producer to do good job, but they need good financial to implement the good farm biosecurity, but when they implemented they are selling to the market, the consumer didn't aware and not respond to the product, so they want cheap, cheap, cheap, but don't want to pay, so at the end it kind of, the circle everywhere, not goes nowhere.<br><br>R: … how would you sort of shape consumer demands…<br><br>P: … I think the number one because the customer can create the demand, because they are in between consumer and the producer.<br><br>R: And how would you get the … demand [changed]?<br><br>P: We think about it at the beginning we think like oh maybe it is kinds of policy education, but I think it is too big picture. So I think maybe has consumer when it has to be like bottom up a little bit. It may start from the school.<br><br>R: So start early in school.<br><br>P: Yes, because the school in the country is kinds of small node, but distribute to other countries, so if we are talking about the education policy to push that, I think it is very difficult, but go through the school curriculum or something, maybe it is kinds of can make it.<br><br>R: Embed education around antimicrobial resistance and good production and that sort of thing?<br><br>P: Yes for the students. Yea, and that might try to change a bit of the advantage in education things for the people.<br><br>R: Moving in over time. That is great.<br>…<br>P: But for the biosecurity I think it depends on, usually for the big company it is high impact, but easy to do, because we have financial enough, but for the low, like a small farmer, SME or something like that, it could be high impact, but hard to do for them because they don't have enough financial to support…to [do] biosecurity. So I think it can be like, it is easy if it is a big company, but for the small holder it could be difficult.<br>…<br>P: Can I add to that?<br><br>R: Yes.

	P: Because I think you sort of hit the nail on the head. We are also sort of thinking about education to create the awareness, but I would agree it is difficult to do and it is because of what the problem of AMR is in our society. So when you think about, if there are things like Ebola it is very easy to get some things into, into schools, in to the wider public to get people aware of what they have to do and what they not have to do. So I sometimes wonder are we really serious about the whole AMR problem, or are we not serious about it, and as long as we are not serious about this as a society, we were here, but the society nothing is going to change.<br><br>P: [Is there] AMR education into the school in other countries?<br><br>P: Not that I am aware of, but I think that is probably something more as at global level. I don't know about the [inaudible] but I think it is really something to start thinking about. So how can we maybe sort of thinking about the next generation, how to make them more aware of it.<br><br>P: Because I note down, sometimes I think maybe certain actions need top down.<br>…<br>But many actions sometimes it doesn't need top down, but bottom up. …Like education. So that is why I didn't think always policy will be the answer for any actions. Yea.
	Day 2 workshop:<br><br>P: I am looking at my perspective as educating food waste in this country, like what I know is we actually are practicing a very rotten food system locally, you know. And I think starting all are quite man made from people like us. Like what the (name of participant) was mentioning. We demand perfect food, and want it cheap and so on. And on the lifestyle, the fast food, the convenience store frozen food and all these things we have created convenience is all come from this people, and the population is growing…<br>…<br>In that sense, that to speed up, because our rejection of imperfect food we need to, farmer has got to plant more to sort of offset the rejection. So from the farmer, there is a lot of losses and so forth. It is all in the name of profitability. This is what is happening, and with this, when it comes to production, to do it we need to administer like more antibiotics, herbicide, pesticide, whatever, so that you can get a better yield, you know, and this component is basically here what we are looking at. Of course I am not so technical how to contribute, but I think while we are doing this and me doing the food waste, we need to educate you know in school in my eleven years in secondary or primary school, nothing was taught on all these shocking, you know. We learn to as we move on, but we can inject education everywhere, even from

	small, you know something that people know and learn to respect food. How food comes about. How much resources has been wasted. You know how much of this thing affect our health.<br><br>This is like, it is all known even Americans have now been running a 42% of American are having cancer by the age of seventy-two you know. Fifty years ago cancer is unheard of you know. In my country [name of country], it is 25%, and as the country developed, because you demand [inaudible], percentages will go up, so I think education is important for us to do that. And the food wasting issue, because of our demand lifestyle, what would produce and we know from the people here obesity, five hundred million around the world. Even in my country, [name of country] we are among the factors in this region, compared to… [name of three other SEA countries] and so it is happening. We are basically copying the American model, as we progress [economically], we get larger and so on, I don't think taller. [laughter] So I think we need to inject education everywhere into people. While we start to do all these things you know, because all these also affect climate change you know. Food waste in the land fill emits methane gas, you know, and the resources, the [inaudible] field that need to go into agriculture. It is all lost, you know, the arable land, the water, and all this affects you know, so I don't know where to put these, you know. I think it is important that I do my part, you guys have to do your part. The population will catch up with us if we educate. So there will be reduction.
**Awareness and understanding:**<br><br>Health care sector / prescribing, diagnosing, treatment practices	Interview B<br><br>P: The quality of medical training in countries needs improvement as there are inappropriate prescribing practices.
	Interview B:<br><br>There is nothing on the health care provider - physicians, pharmacists - but they are important because they have an influence on prescribing drugs or dispensing them. This has an impact on use (appropriate/inappropriate).<br>…<br><br>The real front-line works and patients have insufficient awareness about this issue and they need to be aware to ensure health and well-being.
	Day 2 workshop:<br>P: But then he is talking about like from the human health side, clinicians can have the training, although I don't know if they would be willing. Like a re-orientation.<br><br>P: A part of their curriculum within the medical school.<br><br>P: Yea. Yea. I mean I think they are probably hearing it.

	P: I think so.<br><br>P: Yea, they tell people to take the whole package of antibiotics.<br><br>P: Yea, exactly.
	Day 1 workshop:<br>P: …Okay so that is step one. The major one which I would like to talk about, which we talked about in the last couple of days is diagnostics access to antibiotics and making a diagnosis in the first place of what the infection is and testing that for antimicrobial susceptibility.<br><br>P: Can I intervene<br><br>P: Please.<br><br>P: So one of the big problems when you say the word diagnosis, if the young kids on the block do not know their approach.<br><br>P: No.<br><br>P: They are not taught the approach.<br><br>P: No that is right.<br><br>P: So for example, sorry microbiology or pharmacology we are taught modelling of a bacteria, put it this way, it like this and it like this. We are not taught about what it can do and how we interpret the culture and sensitivity or how to approach when is a gram positive or a gram negative, etc. etc. So what happened and just before that or that actual lab result, and we were talking about this earlier, two days back, most of our teachers would concentrate on the history and get a preliminary diagnosis just by listening to the patient. Just by listening, and then you examine and confirm what you think and then take one or two tests and get the diagnosis.<br><br>P: Today super specialists and beyond they do the opposite. They know how to tick all these dots, but they go by exclusion. So this is especially very important in low and middle countries, where we can say all we want, let's have this many diagnostic facilities, this many equipment, this many trained personnel, but we know it is a long run. So we have to improve the approach, teaching and the interpretative and problem solving skills, as well as developing clinical algorithms, which help those physicians to assimilate that data they get from the patient, and sort it out in their mind.

	P: Absolute right, and that is why I use the term stewardship. It encompasses all of what you what you succinctly put very nicely stated. It is not just the availability of services. Ultimately it has got to be holistic process involving training people to take the right sample but interpreting the result at the end of all of that, then it goes around in circles.<br><br>P: And you can make that link to livestock as well.<br><br>P: Exactly.
**Awareness and understanding:**<br><br>Food chain actors	Day 2 workshop:<br><br>P: So we talked about intensifying farmer, producer education about healthy farming systems. It is happening now, but apparently it could be improved, so that people can prevent illnesses that they might have to use antimicrobials for.<br>…<br>P: You need to say like educate the farmer/producer on best practices.<br><br>R: Okay. Educate.<br><br>P: The users. The users yea. There are the users…Whoever is buying it, you know for use.<br><br>P: Best practices, like best farming practices.<br><br>P: Yea, exactly.<br><br>R: Okay….Is there any skills training in that or is it education? What would it look like?<br><br>P: Education which means creating awareness and training and so on. It is all encompassing, yea.<br><br>P: Good agriculture practices.<br><br>P: I mean I think [government] extension agents are doing this with farmers now but maybe with an eye toward reducing antibiotic use.<br><br>P: Yea.<br><br>P: Like not that they need to know about antimicrobial resistance, but maybe the basics of why it is bad to overuse antibiotics.<br><br>P: Right. Right. This is what we tell a lot of our [government] extension agents around the region when we talk to them. You know because I think many countries are very much into this.

<table>
<tr><td></td><td>R: So the [government] extension agents. I have heard this different terms called for, people who work with the farmers.

P: Yea.

P: Government officials who go around and help farmers with their farms. That is my broad understanding of them.

P: Plus. Plus, [government] extension agent is one part of it, but whoever uses them, like even doctors you know, clinic doctors. They also have to be very careful about prescribing antibiotics.

P: Or clinicians.

P: Clinicians, yea.

R: They are a mechanism to do this.

P: Yea.

R: Or they need the training?

P: {Government] extension agents probably need more training, then train farmers.

P: Yea. Yea.</td></tr>
<tr><td></td><td>P: The antibiotic use in livestock. Yea?
…
P: Apply good farming practice on the farm. And also doing [inaudible]

R: And that was stewardship?

P: Yes. Stewardship for farmers training… for something like this.
…
P: The challenge is, the producer of course, would like to sell. It is not impossible [inaudible] to the farmers and also the second one is the farmer perception.

R: And from the perception of …

P: Regarding what antibiotics [farmers] should be using for the livestock. I will give you an example. Since last year [name of country] ban for ATP, but I just attended [inaudible] the private sector company last month, even though we ban ATP in [name of country] but the antibiotics [is] still high, they still put it in livestock. Because the farmer worry, because they worry… how to protect the farm.</td></tr>
<tr><td></td><td>Day 2 workshop:</td></tr>
</table>

	…<br>P: But I think… I think the message can’t just be don’t use antibiotics, you know. We were talking about like a message about like a healthy farming system, so that people can… not even dream of, you know like so that they can just meet…
	Day 2 Workshop:<br><br>…<br>R: I think, yea. Is that an issue of awareness and education possibly?<br><br>P: Yes. Yes.<br><br>R: Okay. So we have food safety. I have a big thing here on everything. Food manufacturing preparation, food retailers.<br><br>R: Yea, it goes right down to the individual level, like in their own kitchen.<br><br>P: The whole [inaudible] chain.
	Interview A:<br><br>P: Yea, I guess a few key points. I think there is still this, there is still this discourse that obviously relates to the impact of antibiotics on humans absolutely and that is undeniable, but there is less discourse about actually how AMR relates and will impact the livestock industry and systems per say as well, and this relates to not only the critically important antibiotics but those have been used already for decades and we know there are high levels of resistance, tetracyclines. The penicillin related groups, etc, and I think adding into the awareness, obviously high welfare systems, prudent use of antibiotics, so we talk about responsible use of antibiotics. We are not promoting no sure, because of course that can have a welfare impact. We are talking responsible use, meaning no growth promoting use. No metaphylaxis use used to treat sick animals and prevention you know clinical outbreak, and obviously awareness on the impact and other relationships of disease, but also awareness that the status quo and any high end aquatic use or antimicrobial use is a big red flag really for the sustainability of farm animal and livestock systems, because ultimately where you know 60-70% of resistance to tetracyclines
	Interview A:<br><br>P: yet with the certainly being a raison d’etre, …trying to raise awareness of the need to improve, fundamentally improve animal welfare in the farming systems, as part of the solution if you like, and again we see that in [names of countries outside of Asia] …and elsewhere, where concurrently they manage to reduce their antibiotic use and it is not always emphasized, though there was a recent paper from Barton in England, talking about this pig and poultry industry, and

	how they manage to reduce antibiotic use with the improvements in welfare, and that example actually from Barton, England was also high lightening the use of a breed.<br>…
	Interview A:<br><br>P: Part of solutions or higher welfare – the lack of good practice case studies, like those emerging in [names of different countries] where animal welfare was part of the solution to reduced AMU.
	Interview A:<br><br>P: Yea, it is sort of part of, initial production systems but the genetic companies and the demand of course from the industries really drives that and it is, it is happening faster in some Southeast Asian countries than others. It is sort of reached near max in [name of a SEA country] for example, but in [name of another SEA country] broiler genetics, there is still a gap in growth rate potential, and that may correlate with endemic illness and lead to antimicrobials. So it definitely can impact, but that bargaining definitely showed a decrease.<br>…<br>P: …because it is duplify another actor, which is usually what happens with genetics in these countries, certainly the poultry, one company owns the subsidiary of the main genetics. So again [name of company]… owns the main, the largest genetic subsidiary of the global one, the … one for example, and that then they have a double drive to see that in the system not only primarily in conventional production, but for their sales in the industry. On top of that then sticking with the poultry, for the pig industry, they derive their genetics more from still from the [regions in other parts of the world] …But another thing that makes me think about all of this is that…definitely an influencing factor is the awareness of industry and farmers on this topic, which of course will vary depending between the integrated companies, sold to farmers, etc. etc. So I am just thinking in terms of starting to think about you know solutions and interventions.
	Interview A:<br>Transport and slaughter mentioned – but perhaps not wet markets (for animals) – a high risk area for stressed animals shedding high levels of faecal bacteria and AMR. I can’t stress enough the major issue of informal and inhumane slaughter (even in ‘formal’ settings) in SE Asia – it is truly a hot bed of AMR risk for workers and food systems (aside from cruelty) which is documented for various zoonoses also. A lack of knowledge, training, standards and regulation persists….
	Interview A:<br><br>P: …but farm workers, veterinarians, and their families, interrelated, there is a direct relationship obviously with carriage of AMR bacteria and other micro-organisms.<br>…

	P: There are plenty of studies to show that farm and slaughterhouse workers I might add are sometimes up to ten times more or have ten times the prevalence of AMR bacteria. Streptococcus Suis is a classic, but there is many others.<br>…<br>P: I think they assume an aspect, where I see you know I think there is a differentiation as I mentioned before between farm and slaughter house workers and vets. I think they are their own potential node, because they are exposed, but they are not using antibiotics directly, but there are key risk factors as I mention and then it is that might lean towards a specific intervention or awareness. So I guess if you had to, I am putting my campaign head on now, but if you had to think about the most impacting interventions for awareness, you know that would be a key one versus general awareness of consumers.<br><br>So it may be useful thinking about how you ultimately are going to use this in interventions and innovations. If you had an extra node for those people at least, that group of stakeholders, which is mostly your vets, farm workers and sort of house workers. Those three are particularly high risk, and of course only some of those can influence the system, and the other actor would be you know corporate. So the sort of, there is all the different things but in terms of an actor to where you might influence it, there is obviously some small holders.
**Awareness and understanding:**<br><br>Policy makers	Day 2 workshop:<br><br>P: Vaccine.<br><br>R: Yea. The vaccines you mentioned. Thank you. I meant to write that in. We need vaccines.<br><br>P: You should write autogenous vaccine. They are locally produced.<br><br>R: Autogenous vaccine.<br><br>P: This would be the cheapest and most effective in LMICs.<br>…<br>P: The main point is that they are not commercial vaccines but many countries do not develop vaccines in [name of developed country] to come to [name of country in Asia], so you have got [inaudible] and they use their technology and develop vaccine using the local [inaudible] localized pathogens [inaudible] use and use their technologies [inaudible], they cannot bring the vaccines from your country. So there are very strict restrictions in many countries.<br><br>P: And they probably won’t work anyway. [P: they might not work] So actually making vaccine is the easy part. The hard part is how to convince [inaudible] like in [name of two SEA countries], they don’t have lots of vaccine, because they just make it very hard.

	R: To get them to adopted?<br><br>P: Yea, because they need approval.<br><br>R: To get it approved.<br><br>P: Yea, get it approved.<br><br>P: They might need a shift in thinking as well for the decision makers, because they need to understand what is the bacteria and that it is safe.<br><br>P: This falls under technology dissemination.<br><br>R: Yea.<br><br>P: Like there are so many technologies that need to be like disseminated from developed to developing countries…<br><br>P: We need to educate the policy makers also. Yea.<br><br>P: Yea, educate the policy makers.<br><br>P: Give them a master class in vaccine production.<br><br>P: And that it is safe. They have the misconception that is not safe.<br><br>P: exactly. We see it with humans, with kids
**Awareness and understanding:**<br><br>All sectors, particularly leadership at all levels	Day 1 workshop:<br><br>P: It is called influencing policy makers. Plus this may not be only at the top level. It can be in every unit or every section can be different leadership. So the thing is most humans like to have recognition, right, and they like to be able to achieve something and they like to be in the limelight. Loneliness is probably one of the greatest problems in the world. So if we use those core components and you are able to influence the leader, saying that you look good in the eyes of the people, you will actually be able to do something positive for the people, and if you are a politician, you will likely win the election with those two points, right? Then we have to link the AMR into that psyche.<br><br>R: So how …<br><br>P: So for example, for example, let’s say many places have a problem with hygiene and so if they have an active hygiene problem, and there is visible demonstration of improvement in people’s lives, and they

	know that this actually will be well played out in the media, etc. etc. etc. Probably something that they would be willing to try out.<br><br>R: So who would be the influencers? Who would influence the policy makers do you think? And is it different at different, are the policy makers different at different levels...<br><br>P: … Identify who has the Prime Minister's ears? Let's put it that way, Justin Trudeau included. [Laughter] Right? And there are people that they listen to, right? There are people that they don't listen to. I mean we have to find out who they are, and often it is bureaucrats, certain bureaucrats, and we and this is typically, village leader also, they have to depend on someone. They are not, you know, the all in all. So if you are able to convince that person, even if you don't have direct access to THE person, then I think we get half the battle won.<br><br>And again it depends on the background of the leader, but we have to have clear messaging. We don't have too much time. Okay. We don't have too much time with them, so probably an elevator pitch strategy is important and some people may ask for evidence. Some people may not. Okay. Depends on how you pitch it, but… this is my hypothesis, I am very sure that in spite of everything that we are doing in terms of AMR interventions, that we discussed yesterday, if we are able to get the leader on board. In today's world where even democracies look towards a particular leader, for whatever reason. We can't shift it.<br><br>R: And what are some challenges to do that?<br><br>P: Access to the leader. Okay. Often they have crisis which they have to, even if they have ears, they might want to deal with the next crisis, and therefore just gets brushed aside and the third aspect is often money. If it is a very investment-oriented campaign/interventions, then they will think twice. Nobody has money, unless they want to go to war.<br>…<br>R: So you need someone who is in the leadership position.<br><br>P: Each side will differ depending on the country culture.<br>…<br>P: We have one more… one more key requirement that is evidence sometimes[inaudible] they should not think that this is [inaudible] and believe it and take that, [inaudible] to show the so called policy maker to leaders too. Evidence is a must.<br><br>P: Not all leaders, but yes many.<br><br>R: The evidence here. Some want it. But we need strong evidence, at least have it ready.

	P: Keep it ready, yea, keep it ready.<br><br>P: Otherwise they are going to take AMR again as another… story.<br><br>P: Another spin.
	Day 1 workshop:<br><br>P: One of the things currently we have a window of opportunity as far as AMR is concerned, but it is fast closing in my opinion That has created, I mean I know this because in 2001 there was supposed to be a resolution on AMR in the World Health Assembly at that time, and at the same time 9/11 happened and that is that, because nothing got done. WHO had no personnel AMR and one person struggling with AMR, we know personally in Geneva for many, many years with no money. Then by 2012/13, there was a series of events. I don't want to go into the details and that opened up with the global action planning etc. etc. [inaudible] to term. Okay, and we don't know when this window will close. So why I am mentioning this is in this discussion, easy and hard, but it also is time dependent.<br><br>R: Yea. That is a good point.<br><br>P: So unless we [act] very quickly, I mean the world, If we show some success points somewhere, okay. It will be a lost cause among, I don't know the media or the population or policy makers. I don't know how to label it. So for me it is an urgent thing and of course we are from another side of it also, it is urgent, because [inaudible] itself in my hospital…

LEVERAGE POINT: Good farm practices

**Good Farm Practices:**<br><br>Foster multisector collaboration to improve good farm practices (e.g., via finding ways to implement international regulations, animal welfare, biosecurity and other measures).	Day 1 workshop:<br><br>P: Involve the government extension service officers. Sometimes farmers afraid with the officer. [R: ah] Ask if the officer… say to the farmers that should be in different farm, they sometimes will … will following the suggestion<br><br>R: So if there is a government officer, telling them that they need to change.<br><br>P: Yes.<br><br>R: They will follow it.<br><br>P: Yea.<br><br>R: But that doesn't always happen.<br><br>P: Yes of course.<br><br>R: Meaning the government officer is not always present. So are these government officers, I don't understand the context. So they go onto the farm?<br><br>P: Yea. For veterinary service.<br><br>P: Interesting also how to inform veterinary service [inaudible] other companies.<br><br>R: So relating to this?<br><br>P: Yes.<br><br>R: So it is informing the industry, the private companies?<br><br>P: Especially the veterinary service, to how we can work together to solve the problem.<br>…<br>P: Kind of inform this kind of thing based on diagnosis and all of these things.<br><br>R: And these companies, or the producers of the antibiotics, so pharmaceutical companies. So working together with them. So would that involve farmers and other like everybody working together? Who would be working together, this collaborative piece?

	P: The farmers, the state sectors, plus industry.<br><br>R: State?<br><br>P: It is predominantly government.<br><br>R: And industry.<br><br>P: Because actually most of the farmers [inaudible] companies, they have their own diagnostic services also.<br><br>R: Okay.<br><br>P: They are using those diagnostic services for their own purpose to use surveillance to have information for them to… expand or spectrum of [inaudible] or something like that. So this [inaudible] the farmer actually.<br>R: This is the companies.<br><br>P: Yea company.<br><br>P: One of the intervention also working together with the farming association and livestock association team working with the association.<br><br>R: With the livestock association?<br><br>P: Yes and the last one, with the university of course.<br><br>R: Working with the university?<br><br>P: Yes. Because they sometimes… they can tell .to the farmers, why… they choose.<br><br>R: So evidence may help produce the evidence?<br><br>P: Yes, farmers will be more willing with them… solving [inaudible]<br><br>R: So they have credibility.<br><br>P: Yes of course.<br><br>R: Academic research<br><br>P: That is what we give to them.<br><br>R: Has credibility, in the eyes of farmers. Okay and that might, they will pay attention.

	P: Yes.<br><br>R: Any challenges to this?<br><br>P: Basically it involves international standards and everything into one. It is not easy…I mean… actually I work in the [name of organization], these [inaudible] international standards include public rules.<br><br>R: ….So Integrate international standards. [name of organization] examples into local regulations? There is a Challenge to making this happen?<br><br>P: Actually I think is the farmers, the farmer sector, the private sector together, it is not difficult to implement those things, they can [inaudible], it is quite easy to implement but they have conflict of interest.<br><br>R: So the government sector, farmers.<br><br>P: And industry.
	Interview A:<br><br>P: … [implement] biosecurity 'and' animal welfare are separate but interrelated solutions.
	Day 2 workshop:<br><br>P: Yea selection. This is conventional selection.<br><br>P: Selection is not like an alternative.<br><br>R: So it is selection.<br>…<br>P: These conventional selections. Yea.<br><br>P: So it is like a trade selection only. No intervening.<br><br>R: No genetic intervention.<br><br>P: It becomes totally new okay. It is selecting the best of produce in the next generation, so it is in the next generation<br><br>P: Creates, yea. It does.<br><br>P: It is still genetic improvement.<br><br>P: Genetic improvement. Right.<br>…

	P: Not genetic modifying.<br><br>P: Not genetic modification, through genetic manipulation.<br><br>P: Genetic improvement.<br><br>P: Better traits. Conventional.
	Day 2 workshop:<br><br>[conventional selection] and crops [too].

LEVERAGE POINT: Development, access, and availability of alternatives to antimicrobials

**Development, access, and availability of alternatives to antimicrobials:**<br><br>Find alternatives	Interview A:<br><br>P: and the development of alternatives to antimicrobials of course
	Day 2 workshop:<br><br>P: No but I think the idea is as much as you do regulation, you must also have a plan for alternatives. That is the whole idea.
	Day 2 workshop:<br><br>P: And then I think back-to-back I think one of the things that you brought up about developing alternatives. If you are taking out a particular solution, then you have to find alternatives, you know viable alternatives.<br><br>P: Because if you got alternatives to some of these problems you know to solving these problems, then I think people will not want to resort to this.<br><br>P: Yea.<br><br>P: To reduce that situation of desperation you know.<br><br>P: So perhaps better technologies for the farms could be disseminated<br>P: Exactly.<br>…<br>R: Thank you. So do you want to explain that one for me a little more, technology dissemination?<br><br>P: So like for example like aquaculture, like improved tilapia strains that can make people, fish more resistant to disease.<br><br>P: Tilapia improved kinetic.<br><br>P: Artificial tilapia.<br><br>P: Genetically improved tilapia fish or something like that.<br><br>P: Yea.<br><br>R: It is basically genetic enhancement.<br><br>P: Right.<br><br>P: I mean, yea and you can apply that to any crop. Yea.

	R: … What are some potential consequences? Anything. Unintended from this stuff.<br><br>P: Gene. What do you call it? Spread.<br><br>P: Yea. Right. Right.<br><br>P: Potential consequence of genetic pool… gene transfer.
	Day 2 workshop:<br><br>P: I have one more research and development because in time of food science, we are wasting other than antibiotics what we can do. Okay, to treat this kind of problems. Is it only antibiotics, but actually in the past few years, okay actually we quite emphasize on develop some bacteriophage or vaccine or even Nano samples okay, instead of antibiotics. Okay, to kill the bacteria and perhaps this is one way, okay to reduce that antibiotic resistance in the future.
	P: Second thing hard to do. It can be significant [inaudible – too much background chattering] research and development mainly focus on vaccines [R: vaccines, regarding] for the key business, humans and maybe [inaudible]<br><br>R: Okay, humans, animals, livestock<br><br>P: That would be a significant impact on the usage…
**Development, access, and availability of alternatives to antimicrobials:**<br><br>Technologies to deal with food waste (e.g., black soldier flies)	Day 2 workshop<br><br>P: The new thing is if I am not wrong is called black soldiers.<br><br>P: Black what? soldiers? No.<br><br>P: Have you heard? Black soldiers. I have seen on the media is very big in [name of Asian country]. It is like a larva. They actually brought in big numbers and the food waste comes in, which is sorted out, and this larva will pick everything up like a [inaudible]. And they commit this is our future food, but currently they are using it as a nutrient for livestock and so on. It is something coming. There are two small farms in [name of city in SEA country] now. I am scheduled to pay them a visit, how we rescue food waste, those we need to discard [are] given to them, and those that are still good, we will channel to the border. So it is up and coming.<br><br>P: Can you use the antibiotics.<br><br>P: I think you can click into the video. I have seen it on the web site. Black soldiers. You can see all the videos. It is very big. Yea. People are demanding this new trend now.

	P: It would be interesting to see if they are using antibiotic.<br><br>P: So now later they do that.<br><br>P: Because the idea is to eat, humans eat those, fish eat those and all the antibiotics are using them, the difference.
**Development, access, and availability of alternatives to antimicrobials:**<br><br>Research on probiotics	Day 2 workshop:<br>Okay. Probiotics actually if we usually consume probiotics, this will affect our gut microbial …<br><br>R: So we use probiotics.<br><br>P: Yes, actually, nutrition and composition.<br><br>P: A lot of what we consume is prebiotics. So it is like fruits and vegetables and high fibre foods that then feed the gut microbials.<br><br>P: Yea, prebiotic is to feed the probiotic. Probiotic is actually the leading microbial, [Another P: bacteria], yeah, because if we consume the probiotic, yoghurt okay or cultured milk, okay, because the probiotics inside the cultured drinks or cultured milk, okay. Actually they have only have specific strengths okay for the [inaudible] okay. Since they have a limited species of the probiotics, if we always drink the probiotic drinks, okay, so this will limit our species in our gut microbial. So actually the purpose of the probiotics in our tract, okay. The trick is to fight them .. so if we have rare species of the probiotics, definitely we can always win over the food borne pathogens. However if we always consume the same type of probiotic food, this will limit our species of the probiotics.<br>,,,<br><br>P: Do you mean that it's a problem that we are consuming the same type of probiotic?<br><br>P: Yes.<br><br>P: But it is better than not consuming probiotics at all.<br><br>P: This one actually …<br><br>P: And not all of them …<br><br>P: This one, depends on our health conditions. Okay, sometimes probiotics actually are not necessary. Okay, but it depends on our gut health.<br><br>P: Yea you have an innate immune system in your gut already.

	P: Yes.<br><br>P: It is there, and fully functioning.<br><br>P: Yes.<br><br>P: But it is sensitive to disturbances, whether that is positive or negative.<br><br>P: Yes.<br><br>P: But I don't think we can say with like a blanket statement like eating yogurt, the same yoghurt every day.<br><br>P: Actually probiotics, actually the side effect because we use the same species. Yea, because we use limited species of the probiotics, this must raise one of the issue, okay, the cost are limited, or reduced diversity in our gut, and subsequently affects resistance of the [inaudible]<br><br>R: So we connect to the microflora to their affinity to fight pathogens, right.<br><br>P: Yes.<br><br>R: So that would go to… That would help reduce your burden of illness.<br><br>R: And resistance directly, right.<br><br>R: Yea.<br><br>P: Actually, this is still a controversy because some of the scientists say, this will limit the diversity, or reduce diversity of the gut microbiome okay, but some of course, some of course they are pros of the probiotics they will say no matter how probiotics do good for health, okay, but actually we still need more research to prove whether there is, yes.<br><br>P: It's an early field, and I think yea…there is less research for like taking it in pill form, because it is like, questionable what stays in your gut.<br><br>P: Yes.<br><br>P: But there is more research on like …<br><br>P: The same thing with the effective microorganisms, you know in agriculture, that one of the other issues that we were examining in our committee about the effective microorganisms, because they want to increase the soil microflora, you know so then there is an issue, because

	a lot of people got excited about this, you know because the soil is the problem where everything starts with the soil, as much as for human beings and animals, it starts with the gut. Therefore gut microflora for cattle, gut is very important because of the whole micro processes going on in the gut, and therefore eating the right kind of plants and changing the microflora can impact on what comes out you know at the end of the day.<br><br>P: But the environment also influences the gut microflora, so where communities where people raise chickens and cows in their houses. Like those bad bacteria tend to get in the way of healthy gut.<br>P: Right. Right.<br><br>P: So then that has an impact.<br><br>P: But our contention about using probiotics is, when your gut system gets affected because of some infection, you know, especially diarrhea or whatever, then you sort of recompose, you know.<br><br>P: Sometimes, but if you are constantly in this environment, where you are exposed to fecal pathogens, and you develop environmental enteric dysfunction, and your gut microbiome is not healthy and your intestine is not healthy, and then you are not absorbing nutrients that you eat.<br><br>P: Exactly. Sort of has a cascading effect on a whole range of other things.

LEVERAGE POINT: Research, development, and innovation

**Research, development and innovation:**<br><br>**Data on appropriate dose of antibiotic per body weight of animal**	Day 2:<br>…<br>P: Yea…Like for example we need to, in the case of humans, we have complete data of, you know, how it should be, what should be the dose, depending on the body weight, not for the human, considering the human body weight, average weight, what will be the plasma concentration that the drug will achieve in order, and what is the particular infection beyond the, it should be about a minimum to a particular concentration. About a particular concentration, then all you can get the effect. If it is below, then you have a chance to self-producing resistance strain of the infections. So it is very important to have that data, so we don't, I don't know whether we have enough data you know in the aquaculture or in the case of livestock to have what is the [inaudibe] concentration you know.<br>…<br><br>P: Yea, we need to have complete understanding. For example, a drug is given to a human being, human consumption, there for a treatment. Then we know that for a particular drug the dilution in the body, let's say 3 litres or 4 litres, depending upon the volume of distribution. So we know that it is available for volume of distribution, but do we know the data in the case of let's say livestock. What is the volume of distribution?<br>…<br>P: What is the volume of distribution in the case of fish or something like that, you know? So whether it is probably we might not have a lot of GI effect, but it might have a system where it gets you know spreading to the body tissues or body fluids, and then [inaudible].
**Research, development and innovation:**<br><br>AMR in the environment	P: I am just thinking, environmental. It is an area that we are starting to focus more and more on and I know globally there is increasing research on there, and I think there is definitely a need for more research, so I am thinking about the research innovation hub there. Having said that, it is really sort of to document the pathways and the management of effluence.
**Research, development and innovation:**<br><br>Animal welfare	Interview A:<br><br>P: So I don't see any connection between animal welfare and conventional production systems…so I think there needs to be definitely more work…and thinking about how low welfare impact, influences this system in relation to a number of existing nodes [in causal loop diagram/visual model of AMR in the South East Asian food system].
**Research, development and innovation:**	Day 2 workshop:<br><br>P: Right, basically plant injection. We inject. [laughed] It is like a syringe going in. We do a plant injection, but spraying has got some reports

Understanding crop ecosystems (e.g., microflora) and how impacted by AMU and need for narrow antibiotics	coming from Florida says that it is good, but they say less target, you know, less effect on non-targets. We know that non-targets are all the other beneficial bacteria that is there. The whole microorganism of bacteria, which are very, very beneficial. You remove everything by using some of these antibiotics and that sort of triggers a lot of non-targeted, non-primary, secondary level non-target.<br><br>R: Unintended effect.<br><br>P: Exactly. Unintended effects. Yea. So we are to understand that your microflora is just not just one particular bacteria you know. There are so many antagonists. So it creates a lot of problems in the ecosystem. Yea.<br><br>R: Okay, so then, crop resistance. It did go into the soil you found. Right?<br><br>P: Right. Right. Yea. We tried to analyze but there is not much going in basically because it is a lot of distributed cell, and also we don't have the technology to analyze some of these things as what was pointed out. A lot of these things are focused on human interests, but rather the premium is put on human health rather than on animal and plant health.<br><br>R: Okay. So on the animal and human side, we have narrow spectrum antibiotics more specifically target an organism. It sounds like there is a need for having more on the crop side of things though.<br><br>P: Right.<br><br>R: Now, it's similar situation when you give an antibiotic for example for e-coli and it is affecting the salmonella, it affects the system of microbes.<br><br>P: Right.<br><br>R: So could we summarize this and put it on the map as being, if we have more antibiotics that are targeted to a pathogen, the development of more of those would be decreased use and decreased resistance.<br><br>P: Right.<br><br>R: Because we are honing it to, but it is product development, because the antibiotic is very specific to one. Imagine if they went in your gut and only hit campylobacter and didn't touch the e-coli for example, instead of wiping them all out. So I thought that point is very good and it sounds like there even less knowledge about what is happening in the crop ecosystem than the human or the animal gut.

	R: Less resistance, not less use right?<br><br>R: Do we need to link this to bacteria? That might be gut microflora.<br><br>P: Yea. Microflora from the human context. It is three levels you know. You are looking at the animal, the human and plant.<br><br>R: That is right.<br><br>P: And the environment itself, yea? So much a complexity, and I am not so sure whether one is [inaudible] organism. How this whole thing moves you know. So there is a lot of gaps in this.<br><br>R: Yea.<br><br>P: That is the reason why I was asking about the third generation thing, because if you are going to manage resistance, you have got to understand the mechanism.<br><br>R: Oh yea.<br><br>P: How it moves around.<br><br>R: So if they are very targeted you would decrease the change.<br><br>P: At least the selection pressure will be much more reduced, or maybe you will reduce the population that most of the next generation. Hopefully you get the target right, yea? Otherwise it is very broad spectrum.<br><br>R: Yea.<br><br>P: And higher chances of reducing your selection pressure.<br><br>R: Yea, and it is back to your point, that if you don't, if you are sub-therapeutically dosing, because of the route of administration, mechanism of administration, your chance of resistance ….<br><br>P: And those are on non-targets.<br><br>R: Non-targets.<br><br>P: We always tend to forget that.<br><br>R: Yea.<br><br>P: There are so many bacterial… beneficial bacteria.

	…<br>R: …if we target bacteria, we will not change the gut flora. So…<br>…<br>P: Are there targeted antimicrobials that will… will not influence the gut microbiome, like they are that specific that it can just target one bacteria?<br><br>R: Not one probably, but a narrow one.<br><br>P: Like a class?<br><br>R: So some are very broad spectrum, not all gram positive and all gram negatives, but some of it is all gram negatives, but it is really not very home free. [laughed]<br><br>P: That is right, yea.
**Research, development and innovation:**<br><br>Technologies to preserve food, facilitate food safety and food security.	Day 2 workshop:<br><br>P: I think agree. You see technology in all expect from a mobile phone apps, you know GPS technology, transportation, non-invasive surgery, we are still in the dark age as far as preservation of food. Something needs to be emphasized on that. It is not just the antibiotic. We are still very far behind. Everything is on it too. It change the way we live, you know. Food preservation is still the same thing, soft preservation, additive, nitrates and things like that. Nothing like a [3-D] scanner, you can scan everything. It can last like that. Then we may not need antibiotics. We will process the meat using that system, you know. You can extend three years.
**Research, development and innovation:**<br><br>Working with social scientists to understand how to change the supply, demand and use of antimicrobials	P: Yea, and then that we discuss there is a need to maybe better understand the whole behavior around AM use, because that could give<br>…<br>P: What really triggers behavior change?<br><br>P: Exactly. How can we address that? Yea It is like I am not quite sure. There are probably are lots of social scientists in looking at those issues, but I haven't seen much coming out around behaviour change theories.
	Day 2 workshop:<br><br>P: I think the key thing to sort of point, is what are the factors that drive antimicrobial resistance. There are major driving forces, you know. Pull and push factors, yeah? , markets, security, food, affluence, you know, changes in lifestyles, but I think at source, you really look at the front end, you know how do you manage that, because if you could manage that, it is like I think one of the slides you showed about how you could reduce you know the first year, the first two years of your work with salmonella, we are able to reduce the selection pressure, and therefore reduce the resistance. It is so simple, scientifically actually. If you don't have selection pressure, then you don't have resistance pressure.

	So if you could cut that trail out, you know, then you can easily make the other things work, you know, because the issue is about antibiotics, the use of antibiotics, probably indiscriminate use of antibiotics indiscriminate in any form, yea, either dosages, too much, whatever. How do you solve that? If you could cut that in the front end, then everything else is smooth. [Another P: reversing] It is basically it all goes back, that is why the same thing with pesticides, you know. I usually show this when I talk about pesticide resistance. I tell these chemical companies very simple. If you want your chemical to stay for a longer period of time, you just need to reduce, you know, just integrated with other measures, and the moment you stop that, the selection pressure within two, three generations. I am not talking about many generations. It is so fast, it drops, and then you can go back trying to recycle certain things in a different way. So it is just a simple matter of technicality, you know in terms of use, that shakes the whole project in a different pathway totally.<br><br>P: And so how do we manage that front end part of it, so that you… Because all these other things about food distribution, food wastage, everything, it is the bigger picture, you know, that pulls it, but if we stop this, then resistance is reduced and therefore minimized, and then you could easily sort of come to some solutions.
	Interview A:<br><br>P: Research needs – would ideally include more to address the demand / supply / use side of the system, rather than replacements. And ultimately – hone in on the feedback loops.
**Research, development and innovation:**<br><br>Understanding the system and where to intervene by identifying feedback loops	Interview A:<br><br>System feedback loops – [need to]…distilled in some way initially to help focus on effective solutions in SE Asia context.
**Research, development and innovation:**<br><br>Collaboration between high-income and low and middle income countries to learn about diseases affecting low-middle income countries and findings ways to intervene	Day 2 workshop:<br><br>P: Our horticultural products you know. We lost the papaya industry. We are losing the papaya industry. We lost the citrus industry, and we were very happy when Florida got the citrus greening problem, because we thought that would be some… [group laughed], happy in the sense that I am sorry, but not happy in the, not in the wrong sense, but happy in the sense that we know that there is going to be a lot of fundamental research going into it. America will support this. USA is very far ahead, but still they have not resolved the issue.<br>…<br>R: That is a really good point though that you raised that I don't think we have in our model, and it is improving knowledge in the high-income countries that the disease pressures in the low income countries to help find solutions. Because when you said, you know, it was good that

	Florida experienced it because there is research and there is simulation on how to, in an area where there are financial resources. I actually thought that was a point that, I haven't seen on any of the maps before.

LEVERAGE POINT: Underlying intent

**Underlying intent driving the system:**<br><br>Need for global collaboration	Day 2 workshop:<br>…<br>P: You need some global thing, you know that everybody has got to sit together. Same as the climate change you know. Alone you can't do it. It has to be educated, globally or not, whatever our system is failing us, and it is going to kill us by the year 2050. Again, scientists are warning us. We will start to have food insecurity for crisis. With the climate change, agriculture, livestock, and so on. The climate change, the farmer has got to find ways, you know. It is not an immediate fix you know. Right. They are not wiping off one acre, two acre. It can be an entire thing, and the worst is coming. The last few years you can see that the climate is so severe you know.<br><br>P: I think we are in a very consumptive economy.<br><br>P: Yea.<br><br>P: Everybody wants different things, huh. So it is a total mindset change. The economy is a very consumptive base, but it is big business. You cannot run this world without a business, because the business guys are the third force yea. The invisible force.<br><br>P: You become like oil, you will be in control.<br><br>P: Certainly these two approaches are like complementary, so what drives the intensive use of pesticides but also the microbials is the demand for lower food, for certain types of food, like more meat. So if we act on the drivers, our food consumption patterns, and so on, we can also reduce the amount of meat that needs to be produced, and therefore the amount of antibiotics that are used to produce this meat, and of course meat will be produced anyway. It is a matter of producing it with the minimum or the most cost effective or effective better use of antimicrobials. So this prevention is used.
**Underlying intent driving the system:**<br><br>Need for systems thinking to find and address root causes	Day 2 workshop:<br><br>P: What about this, basically I am looking at it from a more evidenced-based sort of you know, we are, I am not so sure, because you know we are not, I don't know much about, I don't read much about these things, you know, but whether this is very much evidence based, you know in terms of really work that is done. This whole complexity of things you know moving in the system and how it sort of affects, because there are so many conflicting sort of views.<br><br>R: Yea, do you mean this sort of process?<br>….<br>P: No. No. I think we, it is a step forward. Yea. I would say.

	R: Oh in terms of antimicrobial resistance.<br><br>P: Right. Right.<br><br>R: …this complexity.<br><br>P: At least you map out the system first and then you see which are the ones that will be the major …<br><br>R: Yea the major drivers through simulation modelling.
	Interview A:<br><br>P: …and any high end aquatic use or antimicrobial use is a big red flag really for the sustainability of farm animal and livestock systems, because ultimately where you know 60-70% of resistance to tetracyclines and they want to maybe you know for live in, or shift to probiotics or something else. It is just shifting the problem and so that is why I was delighted to see you are taking a systems' approach to this, because my concern is if you just increase the hygiene or you just increase the alternatives, then you are just potentially shifting the problem down another decade to somewhere else.<br><br>So hygiene you have resistance to disinfections. You have resistance to probiotics. You have all of them. So the underlying causes of the system are not addressed, it is ultimately, it is really just shifting the problem<br>…<br>So I guess that is the aspect, and then of course the high risk to farm and people, workers, actors and the food chain themselves, and obviously the wide spread even transitional use in relation to plasmids being found in not only in environment but in genomes and past down generations and through things like genetic engineering potentially as well. So I think there is all these unexpected as you say, things that we have to in some way try and anticipate but that is why we strongly refer to I guess a root cause analysis, and really looking at the systems for the root causes of livestock illness and disease in the first place…
Need for global consensus on regulations on AMU for metaphylactic use	Interview A:<br><br>P: So perhaps you know having two that, the prevention of you know clinical and confirmed infections and useful metaphylaxis, and we know that that is happening in a wide scale in many industries. So I definitely think that that needs a separate node.<br>…<br>P: So I think the enforcement and the regulation is generally lax and the observance of it, and then it is mostly focused on from a global push on growth promotion, not his metaphylaxis, and that is where I think there is a real, there is still a tension and that came up at the global [name of meeting] last year, a year ago, and also when I went to

	[name], which is a large industry event for Southeast Asia, that tension still exists between I think certainly Europe and US, but also probably in the middle of that of course Southeast Asia and other low and middle income countries. So yes, that is still a significant area that is a gap I would say.
