# Supplementary material for: Factors impacting antimicrobial resistance in the South East Asian food system and potential places to intervene: A participatory, one health study
Source: Front Microbiol. 2023 Jan 5;13:992507. doi: 10.3389/fmicb.2022.992507 (PMC9849958; doi:10.3389/fmicb.2022.992507)
Supplement: Supplementary file 1 [file Data_Sheet_1.zip › Supplementary File E.PDF]

Supplementary Material E:
98 factors influencing AMR in the Southeast Asian system grouped into 8 One Health sub-areas

CATEGORY THAT FACTORS CLUSTERED AROUND	NAME OF FACTORS INFLUENCING AMR	DEFINITION OF FACTOR	OVERLAPS WITH OTHER CATEGORIES (the factor applies to more than one category)
**Individual, Social & Cultural Factors**			
	AM use in companion animals	Use of antimicrobials in companion animals (e.g. dogs, cats, reptiles, rodents, horses) for all purposes (preventative, control, and treatment)	
	AROs in companion animals	Resistant organisms in all companion animals	
	AROs in humans	Resistant organisms in all humans	
	Chronic, non-communicable diseases	Chronic illnesses in humans	
	Companion animal wellness	Disease in companion animals (infectious and chronic)	
	Consumer choice, demand, and behaviour	Individual level human behaviour, choice, and demand for products	
	Consumption of other (non-meat/egg)	Human consumption of all non-animal-based food products	
	Death		
	Human illness	Diseases in humans	
	Human vaccinations	The human population which have been vaccinated against common pathogens	
	Meat/Egg consumption	The amount of animal-based food products consumed by the general population	
	Traditional home cooking	The amount of food consumed through cooking at home with traditional preparation and fresh foods (compared to eating out at other avenues)	

CATEGORY THAT FACTORS CLUSTERED AROUND	NAME OF FACTORS INFLUENCING AMR	DEFINITION OF FACTOR	OVERLAPS WITH OTHER CATEGORIES (the factor applies to more than one category)
	Non-AM infection prevention and control by the public	All other forms of disease prevention and control done by the general public on a day-to-day basis	
	Food and water security (personal, national)	The state of having reliable access to a sufficient quantity of affordable, nutritious food and clean, potable water	Government
	Nutritional quality of diet	The value of the product for the consumer's physical health, growth, development, reproduction and psychological or emotional well-being	Government
	Population vulnerabilities	Groups and communities at a higher risk for poor health because of the barriers they experience to social, economic, political and environmental resources, as well as limitations due to illness or disability.	Government
	Diverse experiences, opinions, training and culture	The varying experiences and backgrounds of the population that affect how they act and make decisions	Public health, healthcare and social care; Agriculture
	Understanding and awareness	The varying experiences and backgrounds of the population that affect how they act and make decisions	Public health, Healthcare and social care; Agriculture
	Human AM use	Antimicrobials used in humans for all purposes (treatment, prevention, control)	Public health, healthcare and social care
	Use for controlling spread of illness in humans	Antimicrobial used in humans to control the spread of an infection and prevent getting an infection from nearby infected human	
	Use for prevention in humans	Antimicrobial used in non-infected humans to prevent getting an infection	
	Use for treatment in humans	Antimicrobial used in humans to treat an infection	

CATEGORY THAT FACTORS CLUSTERED AROUND	NAME OF FACTORS INFLUENCING AMR	DEFINITION OF FACTOR	OVERLAPS WITH OTHER CATEGORIES (the factor applies to more than one category)
	Host microbiome	All organisms living in and on a host	Environment; Agriculture
**Public Health, Health Care, and Social Care Factors**			
	Access to healthcare	The availability of adequate healthcare services to an individual physically and financially and the right to seek, receive and impart information and ideas concerning health issues	
	Existing healthcare infrastructure	The current physical infrastructure of a given hospital that has the ability to affect the day-to-day operations	
	Healthcare costs	The actual costs of providing services related to the delivery of health care, including the costs of procedures, therapies, and medications	
	Non-AM infection prevention and control in health and social care settings	All other forms of disease prevention and control done in healthcare and social care settings	
	Non-AM infection prevention and control in other social institutional settings	All other forms of disease prevention and control done in social settings that is regulated and not under the control of the general public	
	Prescribing, diagnosing, treatment practices	The practices of a prescriber (physician and veterinarian) in terms of how they diagnose, plan to treat, and prescribe medication	
	Healthcare resources	The physical, monetary, and time resources the healthcare system has to be able to accomplish their work	Government
**Agricultural Factors**			

CATEGORY THAT FACTORS CLUSTERED AROUND	NAME OF FACTORS INFLUENCING AMR	DEFINITION OF FACTOR	OVERLAPS WITH OTHER CATEGORIES (the factor applies to more than one category)
	(Terrestrial) On-farm AM use	Use of antimicrobials in terrestrial food-producing animals for all purposes (preventative, control, and treatment)	
	AM use in plant agriculture	Use of antimicrobials in agricultural plants for all purposes (preventative, control, and treatment)	
	Animal density	The number of animals in a given space	
	Aquaculture AM use	Use of antimicrobials in aquatic food-producing animals for all purposes (preventative, control, and treatment)	
	Animal welfare/stress	How well an animal is coping with the conditions in which it lives both physically and mentally	
	AROs in food-producing animals	Resistant organisms in all food-producing animals	
	AROs in plant agriculture	Resistant organisms in all plants used for agriculture	
	Disease in plant agriculture	Diseases in plants used for agriculture	
	Existing farm infrastructure	The current physical infrastructure of a given farm that has the ability to affect the day-to-day operations	
	Feed inefficiency	The amount of food-product produced relative to the amount of food consumed by the animal	
	Feed quality	The nutritional composition and quality of the feed	
	Food-producing animal illness	Diseases in animals (incl. poultry, livestock, aquatic animals) raised in agriculture	
	Good farm practices	A collection of principles to apply for on-farm, resulting in healthy animals, and safe and healthy food and non-food agricultural products, while considering economic, social, and environmental sustainability	

CATEGORY THAT FACTORS CLUSTERED AROUND	NAME OF FACTORS INFLUENCING AMR	DEFINITION OF FACTOR	OVERLAPS WITH OTHER CATEGORIES (the factor applies to more than one category)
	Non-AM disease prevention and control in plant agriculture	All other forms of disease prevention and control done in plant agriculture	
	Non-AM infection control on farms of food-producing animals	All other forms of disease prevention and control done in the farming of food-producing animals	
	Producer profitability	The producer's ability to use their resources to generate revenues in excess of their expenses	
	Production costs	The costs related to production of food-products	
	Production systems	The main operations, processes, and products used by the producer to generate food products	
	Resistance at the abattoir/processor	The amount of resistant organisms and genes at the level of slaughter and food processing	
	Restocking with animals/eggs at higher risk for infection	Bringing in of animals or eggs from a different farm which can carry new diseases that can be introduced to the herd	
	Disease in plant agriculture	Diseases in plants used for agriculture	
	Use of growth promotion	Antimicrobials used to increase animal's ability to gain weight	
	Use for metaphylaxis/control	Antimicrobial used in animals to control the of an infection and prevent getting an infection from nearby infected animal	
	Use for preventative purposes	Antimicrobial used in healthy animals to prevent an infection	
	Use for treatment	Antimicrobials used in animals to treat infections	
	What is being farmed	The food products being produced on a given farm	

CATEGORY THAT FACTORS CLUSTERED AROUND	NAME OF FACTORS INFLUENCING AMR	DEFINITION OF FACTOR	OVERLAPS WITH OTHER CATEGORIES (the factor applies to more than one category)
	On-farm production level (e.g., kg, L)	The amount of food product (animal-based and plant-based) that is produced for sale by a given farm	Trade, sales, and distribution
**Trade, Sales and Distribution Factors**			
	Amount of imported product	The total amount of food products available for sale that have been imported from a different country	
	Amount of product in the domestic market	The total amount of food products available for sale in the county's domestic market	
	AROs in food products	Resistant organisms in all food products	
	Domestic and international trade	All trade of food-products within and between countries	
	Retail cost of food	The relative cost of food in retail stores	
	Cost per unit (e.g., kg, L) set by quota	Cost per unit of animal-based food products set by quota	Agriculture
	Animals/eggs arriving from a distance	Amount of animals or eggs transported or imported.	Agriculture
	Market price per production unit	Price the producer receives when selling products to the market	Agriculture
	Number of units (e.g., kg, L) set by quota	The number of units that can be produced which is set by the national quota	Agriculture
	Retail availability of meat/eggs in domestic market	The amount of animal-based food products that are produced to the standards for sale in retail stores	Agriculture
	Time to market weight	Time it takes for an animal to grow from farm to market	Agriculture
	Viability of domestic meat production	The ability of the domestic meat production to survive without imports	Agriculture
	Exposure to AROs in imported products	Exposure to resistant organisms through contact or ingestion of a contaminated imported food product	Individual, social, cultural factors

CATEGORY THAT FACTORS CLUSTERED AROUND	NAME OF FACTORS INFLUENCING AMR	DEFINITION OF FACTOR	OVERLAPS WITH OTHER CATEGORIES (the factor applies to more than one category)
	Purchase of food at places at other than grocery store	The purchasing of food at institutions other than chain restaurants and grocery stores (short-chain, small scale, local)	Individual, social, cultural factors
**Environment Factors**			
	AROs in wildlife	Resistant organisms in all wildlife animals	
	AM use in wildlife	Use of antimicrobials in wildlife animals (e.g., racoons, rodents, coyotes, birds)	
	Resistance in the wider environment	The amount of resistant organisms and genes in the surrounding environment	
	Wider environment microbiome	All organisms in the environment	
	Treatment of waste and wastewater	The treatment of waste (human and animal) and wastewater to remove harmful pathogens and	Government
	Air quality and pollution	The quality of the air in terms of pollutants and other irritants	Agriculture; Government; Public health, healthcare and social care; Individual, social and cultural factors
	Disposal of AMs	The amount of unused or unmetabolized AMs that are disposed of in waste or wastewater, or are excreted by the host	Agriculture; Public health, health care and social care; Individual, social, cultural factors; Research, surveillance, development and innovation
	Food waste	All unused or partially used food products that end up in the garbage	Agriculture; Individual, social and cultural factors
	Suitability of commodity to environment	Suitability of food product to the environment in which it is grown	Agriculture
	Urbanization and population growth		Government; Public health, healthcare and social care

CATEGORY THAT FACTORS CLUSTERED AROUND	NAME OF FACTORS INFLUENCING AMR	DEFINITION OF FACTOR	OVERLAPS WITH OTHER CATEGORIES (the factor applies to more than one category)
**Government Factors**			
	National budgets, money, funding, and subsidies	The money or funding available (e.g., relating to public health and health care; research, surveillance, development and innovation; agricultural practices; etc).	
	Access to AMs outside of the system	Obtaining antimicrobials from alternative sources that are outside of the regulations of the healthcare system (e.g., without a prescription from a physician or veterinarian)	
	Healthcare resources	The physical, monetary, and time resources the healthcare system has to be able to accomplish their work	Public Health, Health Care and Social Care
	Private healthcare facilities	Healthcare facilities that are not run or funded by the government	Public Health, Health Care and Social Care
	Quality and access to veterinary care	The availability of adequate veterinary services to a producer physically and financially and the right to seek, receive and impart information and ideas concerning animal/plant health issues	Public health, healthcare and social care; Research; Trade, sales, and distribution
**International Factors**			
	AMU in other countries	Amount of antimicrobials used in surrounding countries and countries that have been connected through travel	Individual, social and cultural factors; Trade, sales and distribution
	International retailers	The retailers (grocery stores, fast food chains) based in other countries that sell food products within the country and may have separate agreements and regulations	Trade, sales and distribution

CATEGORY THAT FACTORS CLUSTERED AROUND	NAME OF FACTORS INFLUENCING AMR	DEFINITION OF FACTOR	OVERLAPS WITH OTHER CATEGORIES (the factor applies to more than one category)
	Movement of people	The physical movement of humans from one location to another (domestic and international)	Individual, social and cultural factors; Public health, healthcare and social care
	Movement of animals	The physical movement of wild and food-producing animals from one location to another (domestic and international)	Environment; Trade, sales and distribution
	Level of resistance in other countries	The estimate of the level of resistant organism or pathogens with resistant genes in other countries which can be connected through trade, travel, or geography	
**Research, Surveillance, Development and Innovation Factors**			
	Corporate profits from AMs	Pharmaceutical industry profits from selling antimicrobials	
	Development of new AMs	The creation, development, and production of antimicrobials	
	Development, access, and availability of alternatives to AMs	The creation, development, and production of any product that can be used instead of antimicrobials	
	New and emerging foods	New types of food which will enhance or replace current food practices	
	Pharmaceutical market, sales, and PR	The marketing and reputation of pharmaceutical companies and pharmaceutical representatives.	
	Diagnostics	All resources used to diagnose a disease in humans and animals	
	Research development and innovation	Work directed toward the innovation, introduction, and improvement of products and processes	
	Science and academia	The research and scientific evidence done in the scientific and academic communities	
