# Supplementary material for: Factors impacting antimicrobial resistance in the South East Asian food system and potential places to intervene: A participatory, one health study
Source: Front Microbiol. 2023 Jan 5;13:992507. doi: 10.3389/fmicb.2022.992507 (PMC9849958; doi:10.3389/fmicb.2022.992507)
Supplement: Supplementary file 1 [file Data_Sheet_1.zip › Supplementary File F.PDF]

Supplementary Material F: Leverage points and suggested associated actions: Types, shallow or deep, and rationale

SHALLOW LEVERAGE POINTS:
Places for intervention that have less potential to change the entire system's behavior to mitigate AMR

**Leverage point for intervention identified by participants**	**Suggested intervention or action identified by participants**	**Type of 'shallow' or 'deep' leverage point for intervention as per Abson et al. (2017) [34] and leverage point targeted as per Meadows (1999) [29].**	**Research team's classification of leverage points as shallow or deep and rationale**
**1. National budget, money, funding, and subsidies** ***(CLD factor)***	***Reduce AMU and AMR by investing in:***<br>• research and development (e.g., developing alternatives to antimicrobials, such as vaccines).<br>• increasing the number of trained knowledge brokers (e.g., crop experts) to provide producers with evidence-based advice and technical assistance on appropriate AMU.<br>• training of health professionals (e.g., physicians, veterinarians, and other prescribers) on antimicrobial stewardship.	Type: Parameters [34].<br><br>Specific leverage point targeted: *Changing constants, parameters, numbers in the system, such as subsidies, standards, personnel* [29].<br><br>Shallow or deep:<br>A 'shallow' leverage point [34] because changing constants, parameters or numbers in the system are unlikely to change the behavior of the whole system [29].	Shallow or deep:<br>Considered 'shallow' leverage point as per Abson et al., (2017) [34].<br><br>Rationale: Investments in the action areas participants identified in column 2 are important to addressing AMR, and yet may be less likely to affect broad system change. For instance, investing in training of health professionals and other prescriber on antimicrobial stewardship is essential but funding may be time limited and thus impacts learning among future prescribers. Investing in the development of alternatives to antimicrobials is necessary yet they may not be adopted by end users (e.g., vaccine hesitancy). Moreover, these investments

			are less likely to evoke broad system changes because the system itself has not changed (e.g., the mindset and corresponding goals and rules that drives how the system operates remain the same) [29].
2. Resistance in the wider environment (e.g., water, soil manure, run-off, wastewater) (CLD factor)	***Control or reduce AMR by:***<br>• installing green buffers (vegetation areas) around farms or water bodies to reduce diffuse pollution, accumulation of antimicrobial residues, and AMR spread.	Type: Feedback [34].<br><br>Specific leverage point targeted: *Negative feedbacks* [29].<br><br>Shallow or deep:<br>A 'shallow' leverage point [34]. Negative feedbacks are self-correcting actions that aim to weaken the feedback power of market or other signals in the system that are undesirable and maintain balance in parts of the system. However, each negative feedback has its own goal that addresses a specific part of the system, making it less likely to change the behavior of the whole system [29].	Shallow or deep:<br>Considered a 'shallow' leverage point as per Abson et al., (2017) [34].<br><br>Rationale: Installing green buffers around farms or water bodies aim to keep antimicrobial residue accumulation and AMR contained in that particular aspect of a system and is less likely to change how the entire SEA system behaves to help mitigate AMR.

DEEP LEVERAGE POINTS: ***Places for intervention that may be harder to implement yet have potential to change system behavior to mitigate AMR***			
**Leverage point for intervention identified by participants**	**Suggested intervention or action identified by participants**	**Type of 'shallow' or 'deep' leverage point for intervention as per Abson et al. (2017) [34] and leverage point targeted as per Meadows (1999) [29].**	**Research team's classification of leverage points as shallow or deep and rationale**
**3. Governance, regulations and enforcement** ***(Overarching factor)***	**Intervene to control the production, distribution, sale and use of antimicrobials, including counterfeit and fake products, to reduce AMU and AMR by:**<br>• strengthening and enforcing the regulatory controls and requirements for facilities to produce and prove that antibiotics for animals and fisheries dissolve, absorb, reach required concentrations and are efficacious and thus less likely to contribute to resistance when used, making them equivalent to human standards if not already.<br>• setting and enforcing regulations to control the production and distribution of different antibiotic categories, and what is available for whom.<br>• registering antimicrobials that are legal to use in agriculture and enforcing compliance to limit black market sales of banned antibiotics and pesticides.	<u>Type:</u> Feedback [34].<br><br><u>Specific leverage point targeted:</u> *Negative feedbacks* [29].<br><br><u>Shallow or deep:</u><br>Considered a 'shallow' leverage point [34]. Negative feedbacks are self-correcting actions that aim to weaken the feedback power of market or other signals in the system that are undesirable and maintain balance in parts of the system. However, each negative feedback has its own goal that addresses a specific part of the system, making it less likely to change the behavior of the whole system [29].	<u>Shallow or deep:</u><br>Considered a potentially 'deep' leverage point in contrast to Abson et al., (2017) 'shallow' classification [34].<br><br><u>Rationale:</u> Participants noted that setting and enforcing a regulation that applies across the One Health spectrum (e.g., livestock, aquaculture, human), such as banning over the counter antimicrobial sales while ensuring equitable access to antimicrobials and medical care by those who need them, could impact the entire system. Additionally, if each of the suggested actions that participants identified in column 2 are implemented, this would impact multiple parts of the system and could yield broad system impacts.

	• banning and enforcing over the counter and online sales of antibiotics while ensuring access to alternatives and equitable access to antibiotics and medical care among people who need them and cannot afford them (particularly in rural areas). If applied across the One Health spectrum, participants noted these measures could be "*high impact*" (Day 1 workshop). The challenge is how to enforce such measures.<br>• instituting and enforcing regulations that require antibiotics to be accessed via veterinary prescriptions so that veterinarians can make decisions about AMU for preventative use and not farmers.<br>• instituting and enforcing regulations that decouple prescription from sales, prohibiting a culture of "*kickbacks*" (Interview B) where health providers (e.g., pharmacists) receive money from suppliers for prescribing or selling antibiotics.<br>• enforcing policies and guidance (e.g., on infection prevention and control measures).<br>• establishing limits on the volume of antibiotics (i.e., how much they can put) in a wider range of foods (animal meat, vegetables) and water that extend through the food chain (e.g., to feed/food processors), and		

	meeting these limits and food regulations.<br>• using country specific data collected on AMR levels and AMU usage in human and animal sectors to develop or redefine policies.<br>• developing clear AMU and AMR benchmarks, measurements and implementation and monitoring plans for industry to understand and use to demonstrate how their actions meet AMU reduction targets and how these actions in turn contribute to successful or unsuccessful achievement of AMR benchmarks.<br>• instituting and enforcing regulations to promote higher animal welfare to reduce AMU and humane animal slaughtering at wet markets and other settings to reduce AMR spread.<br>• requiring people who produce food for the masses to be certified to promote good food production practices and food safety.<br>• implementing traceability systems for food domestically (not just internationally) that are enforced. This requires increasing enforcement capacity which is insufficient in some SEA countries.<br>• calculating the cost of inappropriate antibiotic use and incorporating that cost as a tax or price increase specifically in high value productions		

	(e.g., certain crops or livestock) to reinforce AMU limits on foods through the food chain and enable producers to implement biosecurity and animal welfare.<br>• taxing or increasing the price of animal meat to deter consumption and support a shift to sustainable farm practices that reduce AMU while ensuring access to food among population groups that cannot afford tax increases.<br>• taxing those that irresponsibly sell or use antimicrobials. For instance, taxing production actors that are unwilling to comply with appropriate AMU and taxing commercial feed companies that produce or sell feed that contributes to poor animal diets (e.g., supplying fish meal to fish farms). Food lobbying would be a challenge to overcome.<br>• taxing farmers that monoculture to motivate them to diversify what type of species (e.g., fish species) they grow to improve production of food with greater micronutrients that can improve human health.		
4. Prescribing, diagnosing, treatment practices (incl. prescribing habits, appropriateness of	***Improve prescribing practices by:***<br>• creating and enforcing guidelines and policies in hospitals and national care associations (human and veterinary) for prescribing antibiotics, including providing a list	<u>Type:</u> Feedback [34].<br><br><u>Specific leverage point targeted:</u> *Negative feedbacks* [34].<br><br><u>Shallow or deep:</u>	<u>Shallow or deep:</u><br>Considered a potentially 'deep' leverage point in contrast to Abson et al., (2017) 'shallow' classification [34].

AM, dose, duration and route of administration (CLD factor)	of antibiotics for human versus veterinary use classified into first, second and third tiers. First tier includes antibiotics that can be prescribed by the prescriber. Second tier includes antibiotics prescribed for good reasons (e.g., to treat urinary tract infections in humans). Third tier includes a list of antibiotics that can only be prescribed with authorization by a senior specialist (e.g., senior pharmacist, infection specialist or microbiologist in hospital).<br>• implementing antimicrobial stewardship policies and trainings for clinical and health professionals with reviews or audits of hospital practices to ensure quality care, including examining antibiotic prescriptions.	Considered a 'shallow' leverage point [34]. Negative feedbacks are self-correcting actions that aim to weaken the feedback power of market or other signals in the system that are undesirable and maintain balance in parts of the system. However, each negative feedback has its own goal that addresses a specific part of the system, making it less likely to change the behavior of the whole system [29].	Rationale: Improving and monitoring antimicrobial stewardship policies in human and animal health settings, could improve antimicrobial prescribing and use practices that are embedded in organizational / institutional processes. These changes, implemented in combination with the participant identified actions under the leverage point "Governance, regulations and enforcement" above could contribute to system wide changes that help to reduce AMU and limit AMR.
**5. Treatment of waste and wastewater (e.g., sewage, manure, sludge)** ***(CLD factor)***	***Reduce AMR accumulation and spread by:***<br>• instituting and improving standards of wastewater treatment plants to address antimicrobials/antibiotics that accumulate in the city wastewater.<br>• treating manure before being reused in agriculture to prevent spread of antibiotics and resistant organisms.<br>• instituting and improving drinking water treatment plants to prevent	Type: Parameters [34].<br><br>Specific leverage point targeted: *Changing the structure of material stocks and flows (e.g., plumbing structures, transportation networks, infrastructure)* [29].<br><br>Shallow or deep:<br>Considered 'shallow' leverage point [34]. Unless designing new physical structures, this is a	Shallow or deep:<br>Considered a potentially 'deep' leverage point in contrast to Abson et al., (2017) 'shallow' classification [34].<br><br>Rationale: Improving water, wastewater and waste treatment can improve drinking water, sanitation, and hygiene, which are imperative to health and well-being because they can significantly reduce infectious

	accumulation and spread of resistant pathogens.	shallow leverage point because changing already built structures is slow, expensive, and less common to do, and often only allows an understanding of its limitations and bottlenecks and how to limit factors that strain the physical structure's capacity [29].	disease (e.g., diarrheal disease) and are deemed important to addressing AMR and the Sustainable Development Goals (SDGs) [60-63]. The challenge may be the cost and time it takes to bring water and sanitation treatment service to needed levels including areas where access to adequate clean water is lacking.
**6. Understanding and awareness (incl. surveillance, scientific evidence, knowledge translation, communication)** ***(CLD factor)***	**Although participants recognized that knowledge alone will not change behaviors, they also noted low levels of awareness and knowledge on AMR-related issues in SEA and "*whatever you do in terms of educating people, making people understand…makes an impact*" (Day 2 workshop). Thus participants identified different actors and associated actions where AMR-relevant information should be delivered, including:**<br><br>Media:<br>• engaging and training media to disseminate evidence-based messages about AMR to create a sense of urgency about the problem and need for immediate action; align messaging with national and international AMR initiatives (e.g., World AMR Awareness week).<br><br>Consumer choice, demand and behavior:	Type: Design [34]<br><br>Specific leverage point targeted: *The structure of information flows* [29].<br><br>Shallow or deep:<br>Considered a 'deep' leverage point [34]. A common cause of system malfunction is a lack of information which prevents people from being accountable for their decisions. Thus, changing the information structure in the system to deliver information to places where it is missing or needed can, in turn, enable actors to recognize the consequences of their actions and make better decisions [29].	Shallow or deep:<br>Considered a 'deep' leverage point as per Abson et al., (2017) [34].<br><br>Rationale: Adding, restoring, or making use of existing channels (e.g., the media, government officers) to deliver information can allow actors and sectors to understand issues relevant to the AMR issue, learn how their actions contribute to the problem and motivate them to make better choices.

	• delivering information to consumers (e.g., via campaigns) to increasing awareness of what is involved in food production, including what practices are used to produce food that meet their demands for inexpensive and attractive food and the impacts on AMR. Also, to increase consumer understanding about food consumption patterns and the impact it has on health and food waste to improve willingness to pay more for foods, change market demand and food production practices and systems.<br>• developing and disseminating campaigns to create a sense of social responsibility among the public regarding AMU; explore potential utility of messaging that communicates antibiotics are "*bad*" (day 1 workshop) unless it is used to save lives.<br><br>Youth/future generations:<br>• working with schools at the local level to incorporate into school curriculum education about issues relevant to AMR (e.g. good farm practices, healthy living, impacts of food waste) to create a bottom-up shift in consumer demand and behaviors over time that help reduce AMR.		

	Healthcare sector:<br>• delivering ongoing education and training to front-line health care providers on antimicrobial stewardship and best practices/guidelines for appropriate AMU.<br>• having trained health care providers educate patients about appropriate antibiotic use to maintain health and well-being.<br>• training prescribers (e.g., clinicians, veterinarians) on how to diagnose the type of infection and determine antimicrobial susceptibility.<br><br>Food chain actors:<br>• delivering education and training to knowledge brokers (e.g., government officers) on how to use them appropriately, and then utilize them as a channel to educate and train producers on stewardship (e.g., reasons to no over-use antimicrobials or banned antimicrobials), and on healthy farm systems and good food practices to help change producers perceptions that AMU is the best way to protect health of their animals and help build their capacity to implement good farm practices that can in turn		

	improve AMU practices and shift their management systems.<br>• delivering education to food chain actors (including consumers) on food safety practices.<br>• delivering educating to food chain actors (e.g., producers and feed companies) on AMU for prophylactic, metaphylactic or growth promotion purposes; use of critically important antibiotics in animals, and how antibiotics used for decades now have high levels of resistance (e.g., tetracyclines, penicillin related groups, etc.), and the impact these practices have on the sustainability of aquatic and livestock systems and the need for appropriate use.<br>• delivering education and training to food chain actors on the benefits of higher animal welfare systems and biosecurity measures, including how they are separate but inter-related solutions to reducing AMU and how to implement high animal welfare standards to decrease animal, stress, and disease in farm animals and ensure the sustainability of farming and food systems broadly.<br>• identifying and broadly sharing good practice case studies from relevant places in the world where animal welfare is part of the solution to reduce AMU and meeting targets.		

	• delivering education to food chain actors (e.g., producers) about the growing use of genetic companies to accelerate growth of animals (fast growth genetics) and implications for animal welfare, AMU and AMR.<br>• delivering education and training to relevant food chain actors who are exposed to AMR via work (e.g., farmers, slaughterhouse and wet market employees) on humane animal slaughter and how it can reduce AMR spread to workers and people.<br>• increasing awareness and education (e.g., via campaigns) among high-risk groups and associated stakeholders about AMR and AMR spread, such as veterinarians, farm workers, slaughterhouse employees, corporations to address exposure to resistance on-farm, at the slaughterhouse/processor and in the wider environment.<br><br>Policy makers:<br>• educating decision makers and policy makers that vaccines (e.g., produced using local pathogens for animals) and vaccines for humans, particularly children, are safe to enhance their approval for use.		

	All sectors, particularly leadership at all levels (village, city, nation):<br>• building relationships with policy influencers (individuals that leaders / decision-makers trust and listen to) and equipping them with evidence-based messages to persuade leadership about the urgency of the AMR problem and garner their commitment to address AMR.<br>• sharing stories where there has been success in addressing AMR National Action Plans for AMR and improvements in people's lives from AMR mitigation efforts with policy makers to demonstrate progress and reinforce the need to continue to act quickly and urgently address AMR.<br>• sharing success stories with the media to ensure there is public recognition of leadership's support or role in success stories that have had positive impacts of AMR mitigation efforts to bolster and maintain leadership's commitment.		
**7. Good farm practices (e.g., hygiene, biosecurity, confinement, housing, location/ environment** ***(CLD factor)***	• ***Improve ability of the system to evolve actions to transform AMU and address AMR by:***<br>• fostering collaboration among decision makers and practitioners, including producers, government officers, pharmaceutical and producer industries, government sectors, and	<u>Type:</u> Design [34].<br><br><u>Specific leverage point targeted:</u> *The power to add, change, evolve, or self-organize system structure* [29].<br><br><u>Shallow or deep:</u>	<u>Shallow or deep:</u><br>Considered a 'deeper' leverage point as per Abson et al., (2017) [34].<br><br><u>Rationale:</u> Participants recognized the importance of bringing together different sectors to share and integrate

	academic scientists to discuss and share knowledge and resources, and find ways to improve good farm practices (e.g., by improving implementation of international standards and regulations, implementing animal welfare and biosecurity measures; applying conventional selection to improve traits in next generation of species) and reduce AMU in food production, especially in livestock.	Considered a 'deeper' leverage point than previously identified 'deep' leverage points because sharing and experimenting with knowledge that has been accumulated over time provides opportunities to develop new ways to address a problem, which contributes to the ability of a system to self-organize, a feature that is key to building resilient systems [29].	their diverse knowledge, ideas and resources and identify new ways that help build the SEA system's capacity to improve and transform good farm practices that could have impacts throughout the food supply chain and, in turn, help limit or improve AMU and reduce AMR.
**8. Development, access, and availability of alternatives to antimicrobials (e.g., pre- and probiotics *(CLD Factor)***	***Innovate to address AMU and AMR by:***<br>• disseminating technologies (e.g., that can genetically improve fish) from high-income countries to low-middle income countries to prevent on-farm infections and reduce need for AMU.<br>• increasing the development, dissemination and use of autogenous vaccines that are developed using local pathogens.<br>• developing alternatives to antibiotics such as bacteriophage to kill bacteria.<br>• researching and developing non-antimicrobial alternatives that may reduce AMR and help with climate change. For example, black soldier fly larvae which are increasingly being used as nutrients for livestock and to decompose food waste and organic matter (e.g., food waste, mold) that	Type: Design [34].<br><br>Specific leverage point targeted: *The power to add, change, evolve, or self-organize system structure* [29].<br><br>Shallow or deep:<br>Considered a 'deeper' leverage point because sharing and experimenting with knowledge that has been accumulated over time provides opportunities to develop new ways to address a problem, which contributes to the ability of system to self-organize, a feature that is key to building resilient systems [29].	Shallow or deep:<br>Considered a 'deeper' leverage point as per Abson et al., (2017) [34].<br><br>Rationale: Participant-identified actions, such as disseminating technologies and approaches from high income countries to low-middle income contexts, experimenting to develop alternatives to antimicrobials (e.g., the role of pre- and probiotics and development of vaccines made with local pathogens), requires building on, and evolving, a body of accumulated scientific knowledge and products. Building on this body of

	may contribute to AMR accumulation and spread and methane production.<br>• exploring how pre- and probiotics impact gut microflora and immunity in humans and animals as a means to reduce infections and need for AMU.		knowledge to develop non-antimicrobial alternatives can help improve the capacity of the SEA system to limit the need for AMU.
**9. Research development and innovation (e.g., new technologies and approaches)**<br>***(CLD factor)***	***Explore, experiment and innovate to address AMU and AMR by:***<br>• understanding how to design effective antibiotics for use in animals - what the appropriate dose per weight of an animal (type of aquaculture or livestock) is to ensure optimal concentration levels that do not promote the development of resistance when used.<br>• researching crop ecosystems and their microflora and develop narrow spectrum antibiotics that target specific pathogens in crops.<br>• understanding AMR in the environment by documenting the pathways and management of effluence.<br>• examining the interconnections between animal welfare and conventional food production systems.<br>• exploring and implementing technologies (e.g., 3-D scanners) to promote food safety, preserve food longer, and facilitate food security.<br>• conducting research to better understand the reasons that underlie	Type: Design [34].<br><br>Specific leverage point targeted: *The power to add, change, evolve, or self-organize system structure* [29].<br><br>Shallow or deep:<br>Considered a 'deeper' leverage point because sharing and experimenting with knowledge that has been accumulated over time provides opportunities to develop new ways to address a problem, which contributes to the ability of system to self-organize and is a key feature to building resilient systems [29].	Shallow or deep:<br>Considered a 'deeper' leverage point as per Abson et al., (2017) [34].<br><br>Rationale: Participant-identified actions focused on collaboration with scientists from different disciplines and regions and ongoing research into different areas, such as AMR in the environment and integrating social sciences into AMR mitigation efforts. Building on a long history of accumulated scientific knowledge and exchanging and integrating diverse sources of knowledge and ideas, can produce new ideas, products and approaches that build the SEA system's capacity to address AMR.

	AMU and working with social scientists develop interventions that reduce demand and supply of antimicrobials and change behaviors that drive AMU and AMR.<br>• researching how to address the supply/demand and use of antimicrobials side of the system rather than replacements.<br>• researching feedback loops in the system to determine effective actions in the SEA system.<br>• increasing collaboration between high-income and low- and middle-income countries to enable learning about diseases affecting low- and middle- income contexts, sharing knowledge and technologies and working together to find solutions to address these problems.		
**10. Underlying intent driving the system** ***(Overarching factor)***	Effectively tackling AMR, climate change and associated negative impacts by:<br>• Ensuring global collaboration where "*everybody…sits together*" (day 2 workshop) to finds solutions to change the underlying "*consumptive economy*" (day 2 workshop) that drives system behavior and impacts AMR.<br>• Gaining global consensus on regulations to limit AMU for metaphylactic purposes in addition to work that has been done to regulate AMU for growth promotion.	Type: Intent [34].<br><br>Specific leverage point targeted: *The mindset or paradigm out of which the system arises* [29].<br><br>Shallow or deep:<br>Considered one of the 'deepest' leverage points and most challenging to change because it requires changing the underlying paradigm or mindset that drives how the whole system behaves and defines	Shallow or deep:<br>Considered one of the 'deepest' leverage points as per Abson et al., (2017) [34].<br><br>Rationale: Paradigms/mindsets give rise to systems and social agreements about how the world works, which in turn drives system goals and other aspects of the system (e.g., ability to self-organize; the structure of information flows; negative feedbacks, constants,

	• Applying systems approaches and looking at systems to understand root causes of disease and AMR and use that information to identify interventions that effectively and sustainably reduce AMR. Focusing on specific actions, such as just increasing the use of disinfectants for hygiene or use of alternatives like probiotics that can contribute to resistance may be short-sighted because they "*just potentially shift the problem down another decade to somewhere else*" (Interview A).	what all preceding 'deep' (e.g., system design) and 'shallow' (e.g., parameters) leverage points will entail [29, 34].	parameters and numbers) and how they operate. Participants noted that by fostering global collaboration and applying systems thinking, paradigms/mindsets and associated goals can be exposed, challenged and potentially changed to address the root causes of AMR. Thus, changing the paradigm/mindset and associated goals can create a total transformation in the behavior of the whole system.
