# Supplementary material for: Factors impacting antimicrobial resistance in the South East Asian food system and potential places to intervene: A participatory, one health study
Source: Front Microbiol. 2023 Jan 5;13:992507. doi: 10.3389/fmicb.2022.992507 (PMC9849958; doi:10.3389/fmicb.2022.992507)
Supplement: Supplementary file 1 [file Supplementary File D.PDF › Supplementary File D.PDF]

SEA Model

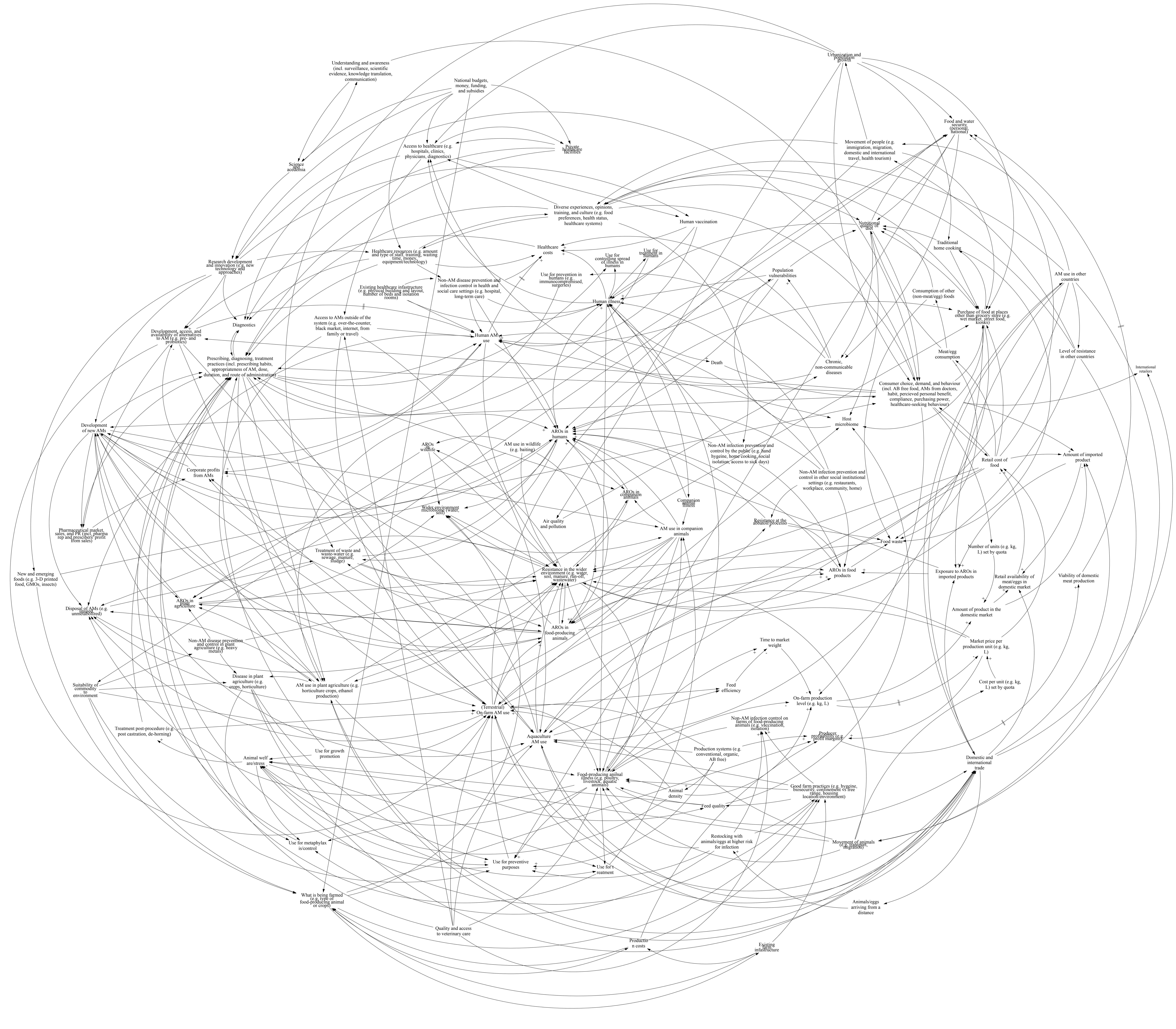

Understanding and awareness (incl. surveillance, scientific evidence, knowledge translation, communication)
National budgets, money, funding, and subsidies
Urbanization and population growth
Science and academia
Access to healthcare (e.g. hospitals, clinics, physicians, diagnostics)
Private healthcare facilities
Food and water security (personal, national)
Movement of people (e.g. immigration, migration, domestic and international travel, health tourism)
Diverse experiences, opinions, training, and culture (e.g. food preferences, health status, healthcare systems)
Human vaccination
Nutritional quality of diet
Traditional home cooking
Healthcare costs
Healthcare resources (e.g. amount and type of staff, training, waiting time, money, equipment/technology)
Use for controlling spread of illness in humans
Use for treatment in humans
Research development and innovation (e.g. new technology and approaches)
Use for prevention in humans (e.g. immunocompromised, surgeries)
Non-AM disease prevention and infection control in health and social care settings (e.g. hospital, long-term care)
Population vulnerabilities
AM use in other countries
Existing healthcare infrastructure (e.g. physical building and layout, number of beds and isolation rooms)
Human illness
Consumption of other (non-meat/egg) foods
Purchase of food at places other than grocery store (e.g. wet market, street food, kiosks)
Diagnostics
Access to AMs outside of the system (e.g. over-the-counter, black market, internet, from family or travel)
Human AM use
Development, access, and availability of alternatives to AM (e.g. pre- and probiotics)
Meat/egg consumption
Level of resistance in other countries
Prescribing, diagnosing, treatment practices (incl. prescribing habits, appropriateness of AM, dose, duration, and route of administration)
Death
Chronic, non-communicable diseases
International retailers
Consumer choice, demand, and behaviour (incl. AB free food, AMs from doctors, habit, percieved personal benefit, compliance, purchasing power, healthcare-seeking behaviour)
Development of new AMs
Host microbiome
AROs in humans
AROs in wildlife
AM use in wildlife (e.g. baiting)
Non-AM infection prevention and control by the public (e.g. hand hygeine, home cooking, social isolation, access to sick days)
Retail cost of food
Amount of imported product
Corporate profits from AMs
Non-AM infection prevention and control in other social institutional settings (e.g. restaurants, workplace, community, home)
AROs in companion animals
Companion animal illness
Wider environment microbiome (water, soil)
Air quality and pollution
AM use in companion animals
Resistance at the abattoir/processor
Pharmaceutical market, sales, and PR (incl. pharma rep and prescribers' profit from sales)
Treatment of waste and waste-water (e.g. sewage, manure, sludge)
Food waste
Number of units (e.g. kg, L) set by quota
Resistance in the wider environment (e.g. water, soil, manure, run-off, wastewater)
AROs in food products
Exposure to AROs in imported products
Retail availability of meat/eggs in domestic market
Viability of domestic meat production
New and emerging foods (e.g. 3-D printed food, GMOs, insects)
Disposal of AMs (e.g. unused, unmetabolized)
AROs in plant agriculture
AROs in food-producing animals
Amount of product in the domestic market
Market price per production unit (e.g. kg, L)
Non-AM disease prevention and control in plant agriculture (e.g. heavy metals)
Time to market weight
Suitability of commodity to environment
Disease in plant agriculture (e.g. crops, horticulture)
AM use in plant agriculture (e.g. horticulture crops, ethanol production)
Feed efficiency
Cost per unit (e.g. kg, L) set by quota
(Terrestrial) On-farm AM use
On-farm production level (e.g. kg, L)
Non-AM infection control on farms of food-producing animals (e.g. vaccination, isolation)
Treatment post-procedure (e.g. post castration, de-horning)
Aquaculture AM use
Producer profits (e.g. profit margins)
Use for growth promotion
Production systems (e.g. conventional, organic, AB free)
Domestic and international trade
Animal welfare/stress
Food-producing animal illness (e.g. poultry, livestock, aquatic animals)
Animal density
Good farm practices (e.g. hygeine, biosecurity, confinement vs free range, housing location/environment)
Feed quality
Use for metaphylaxis/control
Restocking with animals/eggs at higher risk for infection
Movement of animals (e.g. transport, migration)
Use for preventive purposes
Use for treatment
What is being farmed (e.g. type of food-producing animal or crops)
Animals/eggs arriving from a distance
Quality and access to veterinary care
Production costs
Existing farm infrastructure
